# Comparing the Fate and Transport of MS2 Bacteriophage and Sodium Fluorescein in a Karstic Chalk Aquifer

**DOI:** 10.3390/pathogens13020168

**Published:** 2024-02-13

**Authors:** Daniel Matthews, Simon Bottrell, Landis Jared West, Louise Maurice, Andrew Farrant, Sarah Purnell, Danny Coffey

**Affiliations:** 1 School of Earth and Environment, University of Leeds, Woodhouse Lane, Leeds LS2 9JT, UK; s.bottrell@leeds.ac.uk (S.B.); l.j.west@leeds.ac.uk (L.J.W.); 2 British Geological Survey, Maclean Building, Benson Lane, Crowmarsh Gifford, Wallingford OX10 8BB, UK; 3 British Geological Survey, Environmental Science Centre, Nicker Hill, Keyworth, Nottingham NG12 5GG, UK; 4 Environment and Public Health Research and Enterprise Group, University of Brighton, Mithras House, Lewes Road, Brighton BN2 4AT, UK; s.e.purnell@brighton.ac.uk; 5 Affinity Water Limited, Tamblin Way, Hatfield AL10 9EZ, UK

**Keywords:** chalk, karst, hydrologic tracing, MS2 bacteriophage

## Abstract

Groundwater flow and contaminant migration tracing is a vital method of identifying and characterising pollutant source-pathway-receptor linkages in karst aquifers. Bacteriophages are an attractive alternative tracer to non-reactive fluorescent dye tracers, as high titres (>10^12^ pfu mL^−1^) can be safely released into the aquifer, offering improved tracer detectability. However, the interpretation of bacteriophage tracer breakthrough curves is complicated as their fate and transport are impacted by aquifer physicochemical conditions. A comparative tracer migration experiment was conducted in a peri-urban catchment in southeast England to characterise the behaviour of MS2 bacteriophage relative to sodium fluorescein dye in a karstic chalk aquifer. Tracers were released into a stream sink and detected at two abstraction boreholes located 3 km and 10 km away. At both sites, the loss of MS2 phage greatly exceeded that of the solute tracer. In contrast, the qualitative shape of the dye and phage breakthrough curves were visually very similar, suggesting that the bacteriophage arriving at each site was governed by comparable transport parameters to the non-reactive dye tracer. The colloid filtration theory was applied to explain the apparent contradiction of comparable tracer breakthrough patterns despite massive phage losses in the subsurface. One-dimensional transport models were also fitted to each breakthrough curve to facilitate a quantitative comparison of the transport parameter values. The model results suggest that the bacteriophage migrates through the conduit system slightly faster than the fluorescent dye, but that the former is significantly less dispersed. These results suggest that whilst the bacteriophage tracer cannot be used to predict receptor concentrations from transport via karstic flow paths, it can provide estimates for groundwater flow and solute contaminant transit times. This study also provides insight into the attenuation and transport of pathogenic viruses in karstic chalk aquifers.

## 1. Introduction

Karst aquifers are estimated to supply drinking water to 9.2% of the global population [1]. The hydrogeology of these systems is characterised by rapid groundwater flow through fissures, conduits and caves [2,3,4,5]. Karstic flow pathways are often well-connected to critical receptors, such as public water supply springs and abstractions. However, the attenuation of contaminants migrating along these flow pathways can be severely limited. The management of this intrinsic vulnerability requires the delineation of karst flow pathways, and a robust understanding of the processes governing contaminant transport. The value of incorporating contaminant-specific properties into karst aquifer vulnerability assessments has also been demonstrated [6,7].

Hydrologic tracing is a vital method of identifying and characterising pollutant source-pathway-receptor linkages in karst aquifers [8,9]. In densely populated catchments, the effectiveness of fluorescent dye tracers is limited as the visible colouration of the water supplies is generally deemed unacceptable by water providers and regulatory bodies. For example, 10 ppb is generally adopted as the water quality limit for sodium fluorescein dye in England [10]. Employing the precautionary principle and based on the difficulty in predicting receptor concentrations in karst [11], this severely restricts the amount of fluorescent dye tracer that can be responsibly used in experiments.

Bacteriophage tracers are an attractive alternative as high titres (>10^12^ plaque-forming units [pfu] mL^−1^) can be safely released into groundwater environments, offering improved tracer detectability with no risk of visible colouration. In the environment, bacteriophage populations are present at high concentrations (>10^6^ pfu mL^−1^) in both aquatic [12] and soil [13] environments. However, for use as a tracer, bacteriophages are selected based on their absence from the environmental setting of the experiment, e.g., a marine bacteriophage/host bacterium system in freshwater conditions [14]. As microorganisms, the transport and attenuation of bacteriophage tracers are governed by the physicochemical conditions encountered along a flow path [15]. They are therefore classed as a reactive tracer. Contrastingly, non-reactive tracers do not, in theory, interact with the traced system, and therefore a change in concentration is only controlled by dilution and/or dispersion [16]. The term ‘breakthrough curve’ (BTC) is used to describe tracer concentration-time data. Non-reactive tracer BTC data are evaluated and interpreted to gain information about aquifer transport properties, such as transit time, dilution and dispersivity [9,17]. However, the reactive nature of microbial tracers complicates the interpretation of BTC data beyond the establishment of a hydrologic connection and tracer-specific transit times.

Comparative tracer tests allow for the fate and transport of a reactive tracer to be analysed with respect to a non-reactive tracer. This type of test allows for an estimation of the specific attenuation of the reactive tracer at a field scale [16], and for a comparison of advective velocity and dispersion with the non-reactive tracer. This information is critical to assess the use of bacteriophage tracers in karst aquifer vulnerability assessment and management, e.g., source protection zone delineation [18]. 

To the authors’ knowledge, there have been five published field tests comparing the migration of a bacteriophage tracer against a non-reactive solute (NRS) tracer in karst aquifers (Table 1) [14,19,20,21,22]. Other tests in karst terrain have compared non-microbial colloid tracers, such as fluorescent microspheres, with NRS tracers [23,24]. Comparative studies have also been conducted in porous media [25,26,27,28]. With respect to NRS tracers, key observations from these field studies are as follows: (i) in general, massive bacteriophage loss, and (ii) fast colloid migration relative to NRS tracers. Several studies also noted a visual similarity in colloid and NRS tracer BTC shape [23,24]. 

Bacteriophage loss relative to an NRS tracer is driven by one-way kinetic processes, such as decay or irreversible adsorption to immobile or deposited particles. Colloid filtration theory dictates that these processes will impact tracer recovery but not tracer transport [29]. The theory can explain comparable BTC shapes, despite an attenuation disparity. Virus decay is positively correlated with groundwater temperature, and, to a lesser extent, with calcium hardness [30,31]. The strong agitation of samples has also been shown to drive rapid bacteriophage decay [32]. In certain cases, adsorption to surfaces can cause inactivation [32,33]. However, adsorption can also protect viruses from inactivation, and a high proportion of infectious viruses in natural waters are bound to particles in suspension [34]. 

Exclusion processes explain why colloids migrate faster than the average pore water velocity in porous systems [35]. Colloids are excluded from slower velocity streamlines adjacent to pore walls and are preferentially transported through pore space centres; they are also excluded from slower transport via small pore spaces [36]. In karst systems, colloids are hypothesised to be preferentially transported in high velocity streamlines in conduit centres [37].

The primary research question of this study is: Are bacteriophage tracers an effective tool in karst aquifer vulnerability assessment and management? The objectives are to: (i) compare the fate and transport of MS2 bacteriophage and fluorescein dye in a karstic chalk aquifer by conducting a comparative tracer test; (ii) fit one-dimensional transport models to each breakthrough curve to facilitate a quantitative comparison of transport parameter values; and (iii) evaluate the use of MS2 bacteriophage as a tracer in karstic chalk aquifers. The study also has significant implications for the understanding of virus fate and transport in karstic chalk systems.

## 2. Materials and Methods

**Figure 1:**
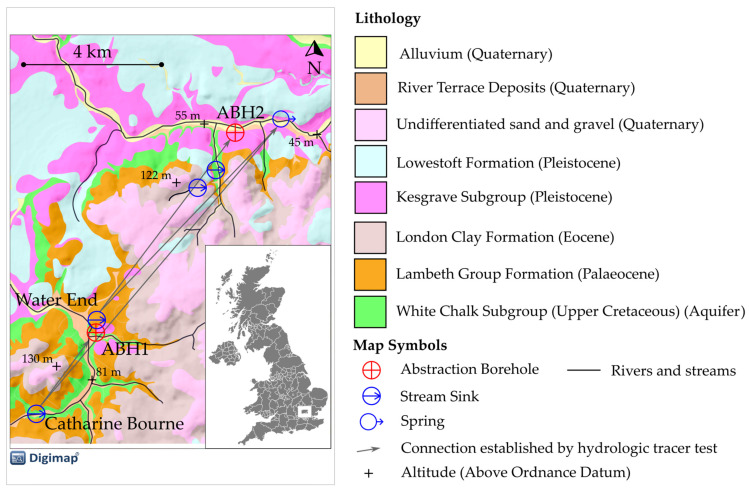
Geological map of field area. The field area is located within the red rectangle on the locator map of the United Kingdom. Geological map data BGS ©.

This study compares the behaviour of MS2 bacteriophage and fluorescein dye in a karstic chalk aquifer in Hertfordshire, England (Figure 1). Tracers were released into a stream sink and detected at two abstraction boreholes located 2.8 km and 10.2 km away, respectively. The release of theMS2 bacteriophage tracer was immediately followed by the release of the sodium fluorescein dye. 

### 2.1. Field Area Description

The Chalk in the field area functions as a karstic aquifer with groundwater flowing predominantly along fissures and conduits [38,39]. The term ‘fissure’ describes a solutionally enlarged fracture with a generally planar cross-sectional shape, and the term ‘conduit’ is a linear solutional void with a circular or elliptical shape in cross-section [40]. Unlike many karst aquifers, chalk porosity is very high (~35%), but small pore-throats severely limit matrix permeability (~6.3 × 10^−4^ m d^−1^) [41]. 

The topography consists of a gently undulating plain that is dissected by numerous river valleys. Altitudes range from approximately 130 m above ordnance datum (AOD) to less than 30 m AOD, and the average annual precipitation between 1991 and 2020 was 714 mm [42]. 

Stream sinks, or ponors, are natural openings in stream beds where surface water drains into subsurface karst features. They are very common in the field area and are generally concentrated on or near to the geological contact between the Late Cretaceous Chalk and the overlying Paleogene sand and clay deposits [43,44]. Sediment eroded from these deposits is transported into the aquifer via stream sink features, impacting conduit development and the hydrogeochemical functioning of the aquifer [45]. Catchments where arable land use is predominant can experience enhanced sediment influxes due to poor soil health and high sediment loads in run-off events. 

The mean tracer velocity recorded in hydrologic tracing experiments from stream sinks to springs in the Chalk was 4600 m d^−1^ [40]. The transport of contaminants from the surface to receptors via karst features is therefore rapid. Contamination attenuation along these pathways via dispersive and diffusive processes can be severely limited relative to attenuation during transport through porous or fractured media.

The land use is mixed but predominantly arable agricultural, residential and industrial. The area is also intersected by major road and rail transport infrastructure. Potential sources of both diffuse- and point-type pollution are numerous, and public water supply wells abstracting from the Chalk aquifer are routinely impacted by contaminants, such as pesticides and microorganisms [44].

A previous hydrologic tracing experiment established rapid flow pathways from the Catharine Bourne stream sink to ABH1 and to a spring approximately 1.5 km east of ABH2 (Figure 1). A more recent study demonstrated rapid flow pathways from the Water End stream sink, another prominent surface karst feature in the area, to ABH2 and the spring [39]. Both studies also established flow pathways to springs and abstraction boreholes to the east of the field area shown in Figure 1. The findings indicate that the Catharine Bourne and Water End stream sink features are connected to the same karst network, and that these networks are hydrologically connected to abstraction boreholes at ABH1 and ABH2.

In this study, tracers were released into the Catharine Bourne stream sink, which has a catchment of 4.92 km^2^, over 50% of which is arable farmland [44]. The catchment is underlain by impermeable Palaeogene sands and clays. The feature is within an oval-shaped depression lined with muddy, silty, allogenic deposits that are pockmarked with small cylindrical holes through which water drains. Point-source recharge via the stream sink is estimated to range from approximately 1 to 10 L s^−1^ [44]. At tracer release, the recharge was estimated to be 2 L s^−1^. The unsaturated zone proximal to the stream sink consists of approximately 15 m of gravelly, sandy, stiff clay. Weathered chalk is presented at about 15 m below ground level (BGL) and intact chalk at 25 m BGL.

ABH1 and ABH2 were monitored for both tracers over a two-week period and were pumped throughout at an average of 39 L s^−1^ (SD = 3.1) and 43.4 L s^−1^ (SD = 1.49), respectively. Water samples were collected every hour from 0 to 72 h after tracer release, every two hours between day 3 and 6, every three hours from day 6 to 7 and then every four hours from day 7 to 14. 

### 2.2. Materials

#### 2.2.1. MS2 Bacteriophage Propagation

In total, 1 to 10 mL of host bacterial culture was inoculated in up to 5 L of a liquid broth that was suitable for host growth. This was incubated at 37 °C and stirred with a magnetic stirrer for approximately 4 to 10 h, dependant on the rate of host growth. The optical density (OD) of the host was measured until an OD of 0.450 was reached (measured at 520 nm for C-3000, ATCC EC15597). Then, 1 to 5 mL of high titre phage suspension (>10^8^ PFU mL^−1^) was added to the culture, mixed and then incubated at 37 °C for 15 min under stationary conditions to allow for phage adsorption to the bacterial cells. The culture was then incubated for 24 h at 37 °C whilst being stirred gently with a magnetic stirrer. After 24 h, the broth containing the propagated phage was then filtered using Thermo Fischer Scientific™ Nalgene™ Rapid-Flow™ (Waltham, MA, USA) Sterile Disposable Filter Units with PES Membranes. The resulting titre of the filtrate was then assessed using the bacteriophage enumeration method described below.

#### 2.2.2. Bacteriophage Enumeration

MS2 bacteriophages (ATCC 15597-B1), from the family *Leviviridae*, have an isoelectric point between 3.5 and 3.9 [46] and are relatively small viruses at 20 to 25 nm in diameter. They were enumerated using the ISO standardised double-agar-layer method (ISO 10705-2; [46]), and the results were expressed as plaque-forming units (PFU) per 10 mL of the sample. For MS2 bacteriophages, the host *Escherichia coli* strain C-3000 (ATCC EC15597) was used for enumeration. Tryptone Soya Broth (Thermo Fischer Scientific™) was used for host strain growth and Tryptone Soya Agar (Thermo Fischer Scientific™) was used for solid media. The concentrations of agar in the top and bottom layers used were the same as those for standardised methods reported elsewhere (ISO 10705/2). Samples were filtered through 0.22 μm polyvinylidene difluoride membrane syringe filter units (Millipore™ (Burlington, VT, USA)/Thermo Fischer Scientific™). For a single assay, up to 5 mL of the sample was added to 1 mL of the exponentially growing host strain and 2.5 mL of semi-solid agar. The resulting suspension was mixed briefly and poured onto previously prepared bottom agar layers in 90 mm diameter Petri plates. Once solidified, the plates were inverted and incubated at 37 °C for 18 to 24 h. Clearly visible circular ’zones of lysis’ in a confluent lawn of host were recorded as PFU [47].

#### 2.2.3. Fluorescent Dye

Sodium fluorescein dye was sourced from Town End Leeds PLC as a solution containing 40% fluorescein free acid, and 87.5 g of this solution was released into the aquifer, equating to 35 g of the fluorescein dye. The mass was calculated using a regression equation (r^2^ = 0.9) based on previous tracer tests in karst [11]. A fluorescence analysis was conducted using a Varian^TM^ (Palo Alto, CS, USA) Cary Eclipse spectrometer. Water samples were measured within a quartz vial and with a path length of 1 cm. Excitation and emission wavelengths were measured from at least 400–500 nm and 450–550 nm, respectively, both with a slit width set at 5 nm. Data were recorded every 2 nm at a scan rate of 3200 nm per minute. Raw fluorescence values for fluorescein were obtained by manual peak picking of an excitation-emission wavelength pair at 490 nm and 511.94 nm, respectively. Fluorescein standards were prepared with ultrapure water and the same batch of fluorescein 40% solution used in the field study. Background fluorescence was characterised in samples prior to tracer release and subtracted from the fluorescein fluorescence peaks. No significant rainfall events were recorded during the sampling period, and therefore background fluorescence was expected to remain effectively constant over the period of tracer recovery. 

### 2.3. Comparative Techniques

Normalised concentration-time plots were used to qualitatively compare tracer recovery, advection and dispersion. Tracer recovery was estimated by multiplying the area under a concentration-time plot (i.e., a tracer breakthrough curve) with discharge (i.e., the flow rate from the abstraction borehole). 

#### 2.3.1. Transport Model Description

A quantitative description of tracer transport can be obtained by fitting an advection-dispersion model to the measured breakthrough curve (BTC) data. In this study, the transport parameter values were calculated for the solute and colloid tracer based on BTC data at ABH1 and ABH2 (Figure 1). The primary purpose of the modelling exercise was to quantitively compare MS2 phage and fluorescein dye transport, rather than to achieve realistic parameter values for a karstic chalk aquifer. 

The parameter values were calculated by fitting an analytical solution of the two-region non-equilibrium (2RNE) model [48], based on the advection-dispersion, to the tracer breakthrough curves. The 2RNE model allowed for first-order mass exchange between mobile and immobile zones [48]. A multi-dispersion model [49] that does not allow first-order mass exchange was also tested. Better model fits were achieved for each BTC using the 2RNE model due to the incorporation of the first-order mass exchange: a process that is known to impact solute and colloid transport in the Chalk [50,51]. Only transport parameters estimated via the 2RNE model are presented and discussed.

Analytical modelling was performed using the MFIT 1.0.0 package [52]. The package applies a multi-flow approach where multiple, independent, one-dimensional (1-D) channels can be incorporated to simulate tracer breakthrough curves (BTC) exhibiting multiple peaks and/or extensive backward tailing [52]. Importantly, the multi-flow approach assumes that mass recovered is equal to mass released, i.e., it does not consider loss via flow divergence or attenuation [52]. This assumption allows for a comparison of solute and bacteriophage transport based purely on BTC shape. 

The transport parameters included in the 2RNE model are discharge (l^3^ t^−1^), mass (M), pathway length (l), mean transit time (t), Péclet number (-), fraction of mobile water (-) and the omega coefficient (l^−1^). The mean transit time, *t*, is:

(1) $$t=\frac{L}{v},$$

where L (l) is pathway length and v (l t^−1^) is advection velocity. 

The Péclet number, *Pe* (-), represents a dimensionless coefficient related to the longitudinal dispersion coefficient:

(2) $$Pe=\frac{Lv}{D},$$

and D (l^2^ t^−1^), the dispersion coefficient, is:

(3) D=δv,

and δ (l), is dispersivity.

The fraction of mobile water, *ψ*, is:

(4) $$\psi =\frac{\theta }{\theta +\theta_{im}},$$

where θ (-) and $\theta_{im}$ (-) are volumetric water contents in the mobile and immobile zone, respectively.

The omega coefficient ω (l^−1^) is:

(5) $$\omega =\frac{\alpha }{\theta v},$$

where α (l^−1^)is a first-order mass transfer coefficient representing partitioning of the tracer to the immobile region via physical (e.g., flow into discontinuous fractures) and chemical (e.g., matrix diffusion) processes.

#### 2.3.2. Transport Model Conditions

The values for discharge, mass and pathway length were known and fixed. t, Pe, ψ and ω values were all fitted. The number of transport channels used in the models was defined by the peak structure of the NRS tracer BTCs. A single channel was used at ABH1, and three channels were applied at ABH2. The initial mean transport times were set based on visual estimations from peaks in the observed data. The initial value for the Péclet number, 250, was based on values for chalk karst from the literature [17,51]. The fraction of mobile water was set to 0.9 in accordance with findings from studies in other karst aquifers [23,24]. The omega coefficient was initially set to 1 × 10^−5^ m^−1^, based on model-derived mass transfer coefficient values for the Chalk [50]. Pe, *ψ* and *ω* values were adjusted manually to achieve a better agreement between the simulated and observed BTCs, prior to model inversion [52]. Wide parameter bound ranges were set for the fitted parameters to reflect the purpose of the modelling study to compare the transport of MS2 phage and fluorescein dye, rather than to estimate realistic parameter values for a karstic chalk aquifer. 

Non-uniqueness is an inherent issue with solutions to the inverse problem. MFIT 1.0.0 utilises a non-linear analysis to assess post-calibration uncertainty [52]. The results of post-calibration analyses based on the recalibration of 500 calibration-constrained parameter sets are included in the Supplementary Materials.

## 3. Results

### 3.1. Tracer Attenuation

The calculations suggest that 1.9 times the injected mass of fluorescein dye is recovered at ABH1 (Table 2). Equipment calibrations have been verified and are not the source of error. The most likely source of error is a discharge overestimation. Discharge values for both sites were provided by the water company. Overestimation of discharge values should impact the recovery calculation for both tracers equally. Overestimation of the discharge rate and thus recovered mass does not impact transport parameter estimation, as explained below [52].

**Figure 2:**
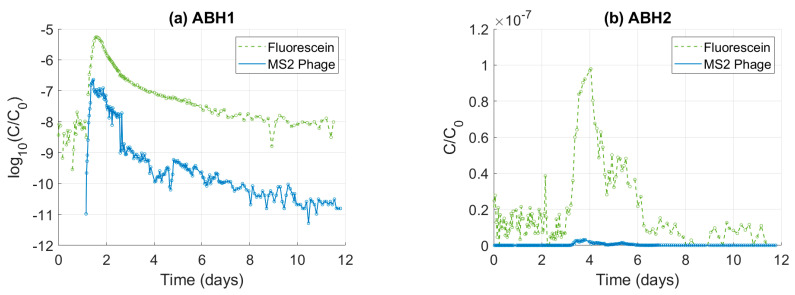
Tracer breakthrough plots normalised to the injection concentration. Plot (**a**) is data from ABH1, and plot (**b**) is data from ABH2.Data from ABH1 are presented as log10(C/C_0_) to make a comparison clearer as the phage BTC is not visible when plotted as C/C_0_.

The relative attenuation of MS2 phage and fluorescein dye can be analysed by normalising the tracer concentration data to the input concentration (C/C_0_). At both ABH1 and ABH2, fluoresceine dye is attenuated significantly less than MS2 phage (Figure 2). For example, peak C/C_0_ is 24 times greater for fluoresceine dye than MS2 phage at ABH1, and 32 times greater at ABH2. 

**Figure 3:**
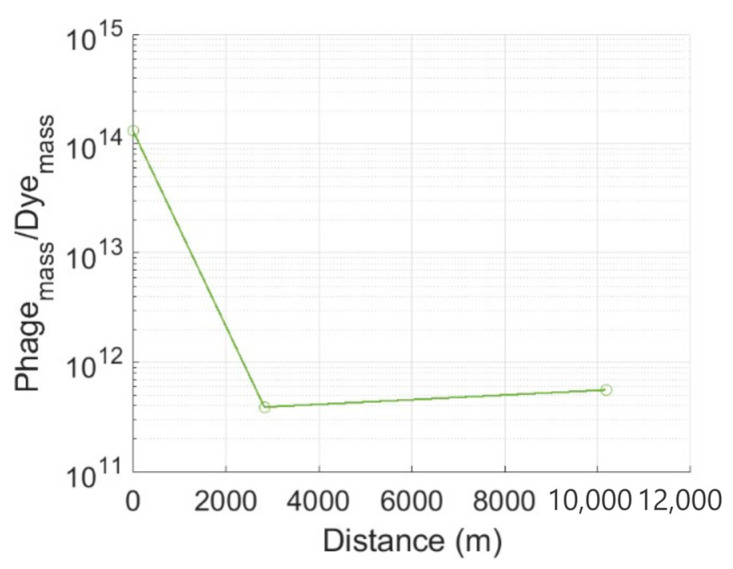
P_i_/FD_i_ for the injection site and P_r_/FD_r_ values for ABH1 (*L* = 2830 m) and ABH2 (*L* = 10,180 m).

Relative attenuation can also be investigated using the ratio of phage tracer to dye tracer masses (Figure 3). At the injection site, the ratio is that of the tracer masses released into the aquifer (P_i_/FD_i,_ where P is phage and FD is fluorescent dye). At ABH1 and ABH2, the value is the ratio of tracer masses recovered (P_r_/FD_r_). The P_i_/FD_i_ value is 337× greater than P_r_/FD_r_ at ABH1, demonstrating a very significant loss of the phage tracer along this flow path. In contrast, the P_r_/FD_r_ value at ABH2 is 1.7 times greater than at ABH1, despite the former being 7 km further from the injection site. This suggests that the attenuation of both tracers is very limited between ABH1 and ABH2, or that the rate of attenuation along this section of the flow path is highly comparable.

### 3.2. Tracer Transport

#### 3.2.1. Normalised Tracer Breakthrough Curves

**Figure 4:**
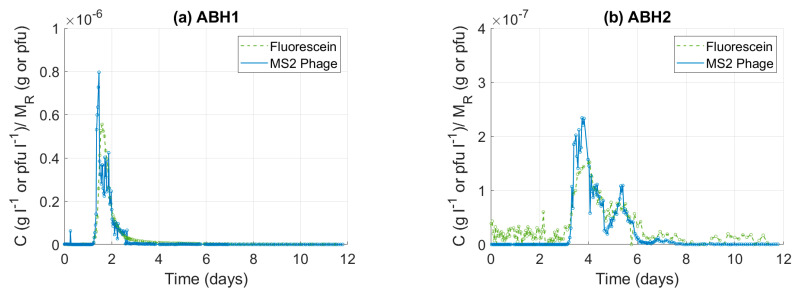
C/M_R_ tracer breakthrough plots. Plot (**a**) presents data from ABH1, and plot (**b**) presents data from ABH2.

When tracer concentrations are plotted relative to mass recovered (C/M_R_), the MS2 phage and fluoresceine BTCs are visually similar (Figure 4). At ABH1, the phage and dye breakthrough curves appear almost identical during the very early stage and late stage of recovery. At ABH2, the similarity is good throughout the period of recovery despite the complex multi-peak structure. At both sites, the phage C/M_R_ peaks exceed that of the dye, by 1.43× at ABH1 and 1.53× for the primary peak at ABH2. When compared to the peak disparity in the C/C_0_ plots, the differences are small. 

#### 3.2.2. Transport Parameter Estimations

**Figure 5:**
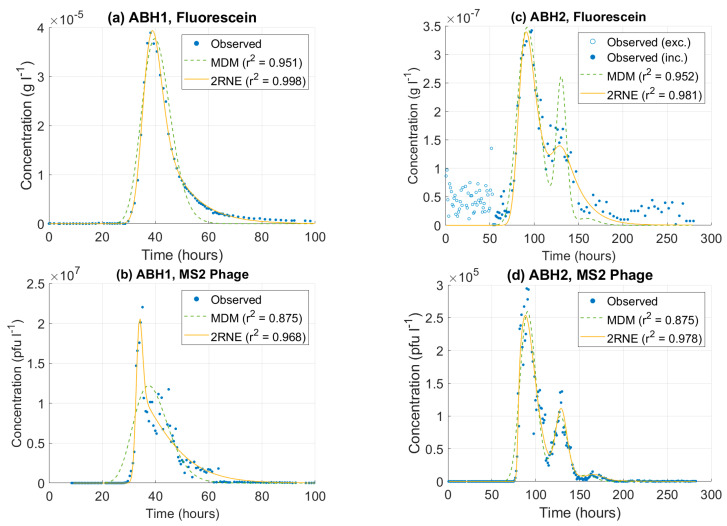
Transport model fits for BTC data at ABH1 and ABH2. Plot (**a**) is sodium fluorescein data from ABH1, plot (**b**) is sodium fluorescein data from ABH2, plot (**c**) is MS2 phage data from ABH1, and plot (**d**) is MS2 phage data from ABH2.

At ABH1, the 2RNE model is able to produce a good fit for both the fluorescein BTC (r^2^ = 0.998) and MS2 phage BTC (r^2^ = 0.968) (Figure 5). However, the model is unable to capture the MS2 phage peak and still produce a good fit for the rest of the BTC. At ABH2, fluorescein data collected prior to 55 h after tracer release are excluded from the analysis as they are interpreted to represent background fluorescence and will therefore interfere with the model fit. The 2RNE model (r^2^ = 0.981) produces a very good fit for the primary peak dye peak but is unable to capture the height of the second or third peaks. The model produces a good fit for the MS2 phage breakthrough at ABH2 (r^2^ = 0.977), but it is unable to capture the highest three concentration data points in both the primary and secondary peaks.

**Figure 6:**
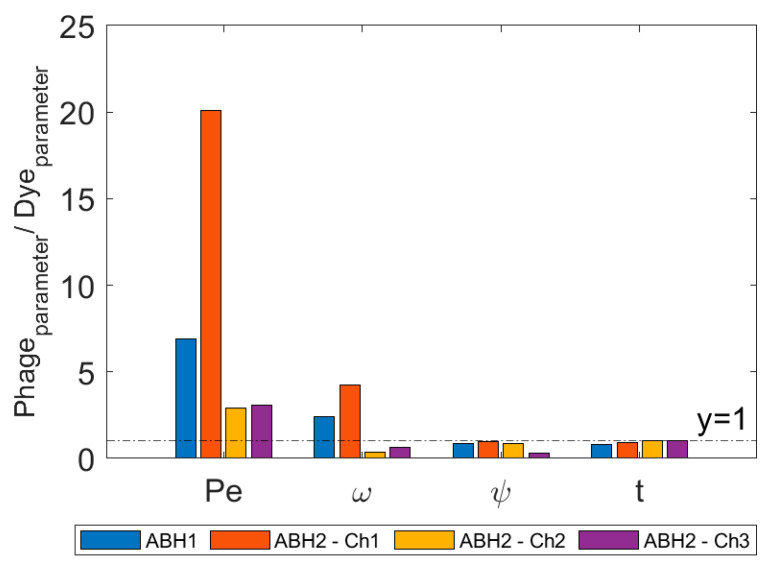
Parameter estimates for MS2 phage tracer divided by corresponding estimates for fluorescein dye tracer.

The transport parameter estimations from the 2RNE model are listed in Table 3. The mean transit time (t) for the MS2 phage is less than the fluoresceine dye value by 15% at ABH1 and 10% at ABH2 (Figure 6). For the second and third peaks at ABH2, the difference in t is insignificant. The Péclet number (Pe) for the MS2 phage exceeds the corresponding dye values by a factor of 7 and 20, at ABH1 and ABH2 (channel 1), respectively (Figure 6). In transport channels 2 and 3 at ABH2, Pe based on phage data is 3× greater than the dye values. The model produces similar estimates for the fraction of flow in the mobile zone (ψ) for the primary peaks at both sites (Figure 6). For the second and third transport channels at ABH2, the ψ values based on phage BTC data are less than the dye values. For the primary transport channels at ABH1 and ABH2, the omega coefficient (ω) estimated using the MS2 phage are 2.4× and 4.2× greater than the estimates produced by the fluorescein dye, respectively (Figure 6). In contrast, phage ω in transport channels 2 and 3 at ABH2 are 0.37× and 0.63× the dye value, respectively. 

## 4. Discussion

### 4.1. Tracer Attenuation

Low bacteriophage recovery, relative to a non-reactive tracer, has been repeatedly observed in comparative studies (Table 2), in limestone karst [14] and in chalk karst [22]. The same relationship has also been observed in experiments comparing the transport of fluorescent microspheres and fluorescein dye [23,24]. The findings of this study support these previous observations.

The unimodal fluorescent dye breakthrough curve (BTC) shape and high C/C_0_ peak strongly indicate a very well-connected flow path from the stream sink to ABH1, despite the uncertainty regarding absolute recovery values. Previous experiments from the Catherine Bourne stream sink do indicate divergent flow to multiple outlets [38,39], and hence the absolute recovery of the fluorescein tracer at ABH1 is expected to be less than 100%. The very large reduction in the tracer mass ratio value from the injection site to ABH1 suggests that the magnitude of phage loss relative to the solute tracer along this flow path is highly significant.

In contrast, the tracer mass ratio values at ABH1 and ABH2 are very similar, suggesting that the bulk of MS2 loss takes place between the stream sink and ABH1. It also suggests that between ABH1 and ABH2, the attenuation of each tracer is either very limited or that attenuation proceeded at a similar rate for each tracer. The pathway from the stream sink to ABH1 includes a 15 m unsaturated zone consisting of gravelly, sandy, stiff clay, and 10 m of weathered chalk in the saturated zone before intact chalk is recorded. Preferential pathways must be present to conduct bypass flow through this low permeability zone, driving dissolution in the chalk bedrock [53].

However, the mud and silt laden condition of the stream sink suggests that any preferential pathway is likely to be backfilled with allogenic sediment. The land use in the swallow hole catchment is predominantly arable agricultural [44], and allogenic backfill sediment will be primarily composed of slightly acidic (pH 5.5–7), argillaceous soils eroded during run-off events [54,55]. The pH of stream water flowing into the stream sink is also expected to be slightly acidic [56]. Conditions in the unsaturated zone will remain slightly acidic as the backfill sediments and surrounding deposits will have a low carbonate content. MS2 phage has been shown to readily and rapidly adsorb to clay particles in similar, slightly acidic conditions [57,58]. The potential for MS2 adsorption to particles in the unsaturated zone is therefore very high (Table 4).

For example, Walshe et al. [58] reported that the mass recovery ofMS2 phage passing through a 2 m column packed with gravel aquifer material dropped from 91% to 31% in the presence of 300 mg L^−1^ of kaolinite, and from 31% to 22% when the pH was reduced from 7.5 to 6. In a trench-trench tracer migration experiment through fractured clay-rich till, the peak concentration of MS2 detected was a 10^5^ reduction of the input concentration, which was injected 4 m up-hydraulic gradient [26]. That equates to approximately a 1.25 log reduction each metre. In this study, the peak MS2 concentration recorded at ABH1 is a 10^7^ reduction of the input concentration. If the rate of MS2 phage attenuation was similar to that reported in McKay et al. [26], the loss of MS2 phage in this study could be wholly accounted for within the 15 m of clay-rich backfill. It should be noted that the advective velocity recorded in both of these studies is significantly slower than observed from the stream sink to ABH1 or ABH2; virus removal is inversely related to flow velocity [59]. Regarding the potential for desorption, chemical conditions in this zone are expected to be fairly constant, and therefore desorption events, generally driven by chemical perturbations such as an increase in pH [57], may not be significant.

The impact of karstic migration rates (>1000 m day^−1^) on tracer adsorption must be considered. The average velocity for fluorescein dye to ABH1 and ABH2 is 72 m h^−1^ and 111 m h^−1^, respectively. The greater average velocity at ABH2 is interpreted to be a function of the additional distance (~7 km) that the tracer was being transported through a conduit network, rather than through the unsaturated zone. Colloid filtration theory dictates that virus removal by adsorption is inversely related to advective velocity [29]. This may be due to a lower collision efficiency at greater flow rates [60]. The mass recovery of bacteriophage tracers has been shown to increase with the flow velocity in column experiments through fractured [61] and porous media [58]. It is significant that rate-limited adsorption is observed in experiments where the maximum advective velocity tested is 44 m day^−1^ [58], as flow through karst aquifers is generally in excess of 1000 m day^−1^. 

The impact of karstic migration velocities on virus adsorption is not well understood. However, it is noticeable that some of the largest bacteriophage tracer recoveries relative to solute tracer recoveries are in migration experiments through extremely well-developed karst terrain in the Swiss Jura and Alps where transport is rapid and predominantly via large conduit and cave systems (Table 2) [14]. The kinetically limited adsorption of MS2 phage to the substrate in chalk conduit networks is a likely explanation for the very limited attenuation of the phage tracer relative to the non-reactive solute tracer between ABH1 and ABH2. 

Temperature has been found to be a strong predictor of virus decay in groundwater [30]. Higher temperatures increase microbial activity, which has been postulated as the primary driver of the increased decay rate [31]. However, in this study, the contribution of decay to total phage loss may be limited by the ultra-short tracer residence times. In a separate study, a 50% loss of MS2 phage was observed in 20 days at 12 °C [62]. Groundwater in the unconfined chalk aquifer of the Lee and Colne catchments varies between 9 and 13°C [63]. Temperature-mediated decay may therefore have contributed to MS2 phage removal, but its significance will be limited, as 98% of the recovered MS2 phage is detected within 3 days at ABH1 and within 7 days at ABH2 (Table 4).

### 4.2. Tracer Transport

Bacteriophage tracers migrating faster than solute tracers through karst aquifers is a common observation in comparative tests [14,20,22]. This relationship has also been observed with other particle tracers in karst [23,24]. Again, the findings of this study support these observations (Figure 6). More specifically, they demonstrate fast MS2 transport relative to a non-reactive solute in a chalk karst system where the potential for phage attenuation via adsorption is very high. Adsorption to large, suspended particles may cause faster phage transport by enhancing the influence of exclusion processes [37] (Table 4).

The difference between the average phage and dye transit velocity is greater along the flow path to ABH1 than ABH2. This indicates that the difference in migration rates between MS2 phage and fluorescein dye is greater in the preferential flow pathways through the unsaturated zone, than in the conduit network. The allogenic infill of preferential pathways renders this zone analogous to a porous system where exclusion processes may be more influential than in the conduit network. 

The mean transit time for MS2 phage is 11% and 16% less than the corresponding values for fluorescein dye at ABH1 and ABH2, respectively. In both cases, the disparity relates to several hours and is not readily visible on a comparative time-concentration plot covering the period of tracer recovery. In fact, the visual similarity between MS2 phage and fluorescein dye BTC shapes is considerable (Figure 4) and suggests that, despite the massive difference in attenuation, the transport of the microorganic and non-reactive solute tracer is governed by similar processes. A similar observation is reported in a comparison of non-reactive solute and particulate tracer transport in karst [24]. This apparent contradiction can be explained using colloid filtration theory [29], where MS2 phage loss is driven by: (i) irreversible adsorption to immobile and deposited substrate and (ii) decay. 

This hypothesis is not strongly supported by the transport modelling exercise. The Péclet number (Pe) values suggest that the MS2 phage is significantly less dispersed in transit than fluorescein dye. An explanation may be that the faster phage transport limits dispersion to a large extent [37] (Table 4). However, it is deemed likely that other processes will contribute to this disparity. The larger difference in Pe values at ABH2 may suggest that MS2 phage dispersion is especially limited during rapid transport via the conduit system. Interestingly, the Pe values estimated based on the primary sodium fluorescein peaks are similar at ABH1 and ABH2, and with other Pe values estimated for transport in chalk karst [17].

Mass transfer (ω) is estimated to be significantly greater for MS2 phage than fluorescein dye at ABH1 and in the primary transport channel to ABH2 (Figure 6). Critically, the mass transfer term in the 2RNE model includes partitioning via physical and chemical processes. Reversible adsorption of the phage to immobile particles [57] and temporary sedimentation of particle-associated phage in slow-flow zones [64,65] may therefore drive the greater mass transfer value. At karstic migration rates through large conduits, solute mass transfer via matrix diffusion is not expected to be a significant process [66]. Hydrodynamic processes, such as flow into a discontinuous fissure, are thought to drive solute mass transfer to immobile zones in karst [17]. Another study applying the 2RNE model to BTC data in a karst aquifer reported high Pe and ω values for a colloid tracer relative to a solute tracer [23]. 

A detailed comparison of the transport parameters estimated for channels 2 and 3 at ABH2 is not conducted due to the poor model fit for fluorescein BTC data across these peaks.

### 4.3. MS2 Phage as a Tool for Karst Aquifer Vulnerability Assessments

The results of this study demonstrate that MS2 phage is a suitable tracer for characterising colloid, solute and groundwater transit times in karstic chalk aquifers. The attenuation of MS2 phage tracer can also provide qualitative information regarding aquifer vulnerability to virus and colloid contamination. MS2 phage is especially suited to this task as it is an established surrogate for pathogenic viruses [67,68] with a considerable body of work investigating its fate and transport in the subsurface, e.g., [25,58,59]. A significant limitation to the application of MS2 phage as a hydrologic tracer is that it does not provide information about solute attenuation or flow divergence, i.e., a low recovery cannot be attributed solely to the existence of a separate flow pathway. 

## 5. Conclusions

1. The attenuation of MS2 phage tracer is massive relative to the fluorescein dye tracer. The disparity is predominantly attributed to irreversible adsorption to immobile particles and decay. Phage attenuation is highly sensitive to the physicochemical conditions encountered along the flow path, and therefore, when applied as a tracer, the phage cannot provide quantitative information about aquifer vulnerability to solute contaminants.
2. Transit times for MS2 phage tracer are consistently less than for fluorescein dye. This is attributed to the influence of exclusion processes in both the unsaturated zone and saturated zone. However, the transit time disparities are sufficiently small to render MS2 bacteriophage suitable for the characterisation of solute and groundwater transit times, and therefore for effective catchment delineation (e.g., source protection zone mapping).
3. A comparison of tracer attenuation and transit times suggests the following: i) MS2 bacteriophage attenuation is concentrated in the unsaturated zone and limited when in transit at karstic-scale migration rates in the conduit network, and ii) phage migrates faster through the unsaturated zone than the saturated conduit network, relative to fluorescein dye.
4. The application of transport models to tracer BTCs in comparative tests can provide a quantitative basis for comparison. In this study, parameter estimates suggest that the phage tracer experiences significantly less dispersion along the flow paths than the non-reactive solute tracer.
5. Future field investigations could focus on improving understanding of the potential for bacteriophage attenuation in the unsaturated zone and along karst flow pathways in the saturated zone. Comparative tracer migration experiments (i) from karst-impacted observation boreholes to karst-impacted abstraction boreholes or springs, and (ii) from stream sinks to monitoring sites at the base of the unsaturated zone (e.g., a shallow observation borehole) would help to isolate relative attenuation information to each zone. These tests should be accompanied by a detailed characterisation of physicochemical conditions to help identify the dominant factors controlling virus and solute attenuation in karst. Associated laboratory studies could isolate the impact of site-specific conditions on tracer attenuation (e.g., particle composition, type and size, pH, organic matter quantity and quality and advective velocity).

## Figures and Tables

**Table 1:** Tracer recovery data for comparative experiments in karstic or fissured terrain.

Bedrock Geology	Test Type	l ^4^ (m)	Q ^5^ (l s^−1^)	Phage	R_P_^6^ (%)	Solute	R_S_^7^ (%)	Publication
Limestone	S-S ^1^	6250	1180	H40/1	21	SR-G ^8^	80	Rossi [19]
Limestone	S-S	6250	1180	H6/1	1	SR-G	80	Rossi [19]
Limestone	S-S	6250	1180	f1	0.15	SR-G	80	Rossi [19]
Limestone	S-S	6500	11,000	H40/1	51	Uranine	56	Rossi et al. [14]
Limestone	S-S	6500	11,000	H6/1	25	Uranine	56	Rossi et al. [14]
Chalk	S-S	5100	200	ϕX174	0.87	Uranine	25	Maurice et al. [22]
Limestone	T-S ^2^	1250	32	H4	2.5	Uranine	23	Auckenthaler et al. [20]
Limestone	T-S	1250	32	H40/1	2.5	Uranine	23	Auckenthaler et al. [20]
Limestone (Epikarst)	Su-Tu ^3^	12	Seepage	H40/1	84	Iodide	59	Flynn and Sinreich [21]
Limestone (Epikarst)	Su-Tu	12	Seepage	T7	17	Iodide	59	Flynn and Sinreich [21]

^1^ S-S—Stream-sink to spring; ^2^ T-S—trench to spring; ^3^ Su-Tu—surface to tunnel; ^4^ l—distance between tracer input and output; ^5^ Q—discharge at tracer output site; ^6^ R_P_—phage tracer recovery, ^7^ R_s_—solute tracer recovery, ^8^ SR-G—sulforhodamine G.

**Table 2:** Tracer recovery data; FD: fluorescent dye tracer.

Parameter	Dye_ABH1_	Phage_ABH1_	Dye_ABH2_	Phage_ABH2_
t_a_^1^ (h)	29.8	27.7	79.3	75.7
t_m_^2^ (h)	38.2	35	97.2	89.7
r ^3^ (g/pfu ^4^)	66.2	2.77 × 10^13^	2.25	1.26 × 10^12^
r (%)	189 *	0.29	6.43	0.013

^1^ t_a_: transit time to tracer arrival; ^2^ t_m_: transit time to tracer peak; ^3^ r: tracer recovery; ^4^ pfu: plaque-forming unit; * high fluoresceine recovery value at ABH1 is discussed in both Section 2.1 and Section 4.1.

**Table 3:** Parameter estimates from 2RNE transport model fits to tracer data at ABH1 and ABH2.

Parameter	Channel	Dye_ABH1_	Phage_ABH1_	Dye_ABH2_	Phage_ABH2_
t (h)	1	38.8	33.7	90	80.9
Pe (-)	1	250.1	1681.4	247.7	4974.3
ψ (-)	1	0.89	0.79	0.89	0.85
ω (m^−1^)	1	2.34 × 10^−4^	5.37 × 10^−4^	8.05 × 10^−5^	3.29 × 10^−4^
t (h)	2	-	-	130	129.6
Pe (-)	2	-	-	260	743.4
ψ (-)	2	-	-	0.9	0.76
ω (m^−1^)	2	-	-	3.99 × 10^−5^	1.6 × 10^−5^
t (h)	3	-	-	165	167.7
Pe (-)	3	-	-	25,803	799.6
ψ (-)	3	-	-	0.9	0.3
ω (m^−1^)	3	-	-	5.14 × 10^−5^	3.41 × 10^−5^
r^2^	-	0.998	0.97	0.98	0.98

**Table 4:** Summary of observations relating to the fate and transport of MS2 phage and fluoresceine dye.

Parameter	Phage vs. Dye	Possible Related Processes
Recovery	↓↓↓ *	Irreversible adsorption Temperature- or adsorption-driven decay
Transit time	↓	Exclusion processes
Péclet number	↑↑	Fast transport limiting dispersion
First-order mass transfer	↑↑	Reversible adsorption Temporary settling in slow-flow zones

* Arrow direction indicates whether the parameter value is observed as greater for MS2 bacteriophage or fluoresceine dye in the study; the number of arrows indicates the magnitude of the observed difference.

## Data Availability

The datasets presented in this article are not readily available because the data include sensitive information about assets belonging to Affinity Water Ltd. Requests to access the datasets should be directed to the lead author.

## Supplementary Materials

The following supporting information can be downloaded at: https://www.mdpi.com/article/10.3390/pathogens13020168/s1. File S1: 2RNE transport model run records and post-calibration uncertainty analyses for the bacteriophage and fluorescent dye tracers at ABH1 and ABH2.
