# Supplementary material for: Comparing the Fate and Transport of MS2 Bacteriophage and Sodium Fluorescein in a Karstic Chalk Aquifer
Source: Pathogens. 2024 Feb 13;13(2):168. doi: 10.3390/pathogens13020168 (PMC10891704; doi:10.3390/pathogens13020168)
Supplement: Supplementary file 1 [file pathogens-13-00168-s001.zip › ABH1_dye_mfit.pdf]

```
                         PEST RUN RECORD: CASE mfit


PEST Version: 16.1


PEST run mode:-

   Parameter estimation mode


Case dimensions:-

   Number of parameters                           :     4
   Number of adjustable parameters                :     4
   Number of parameter groups                     :     1
   Number of observations                         :   500
   Number of prior estimates                      :     0


Model command line(s):-

   MDP_2RNE


Jacobian command line:-

   MDP_2RNE /d
   Jacobian read from file Deriv.txt
   This is an ASCII file.


Model interface files:-

   Templates:
      MFIT.tpl
   for model input files:
      Input.txt

   (Parameter values written using single precision protocol.)
   (Decimal point always included.)

   Instruction files:
      MFIT.ins
   for reading model output files:
      Output.txt


PEST-to-model message file:-

   na


Singular value decomposition:-

   Perform SVD on XtQX or Q^(1/2)X               : Q^(1/2)X
   Max. number of singular values to employ      : 4
   Ratio of lowest/highest singular value        :  5.000000E-07
   Record eigenvectors in SVD file               : yes


Derivatives calculation:-

Param        Increment   Increment   Increment   Forward or   Multiplier  Method
group        type                    low bound   central      (central)   (central)
pgnam        relative    1.0000E-02   none         switch       1.500     parabolic


Parameter definitions:-

Name         Trans-            Change       Initial        Lower          Upper
             formation         limit        value          bound          bound
```

```
h1           log               factor      40.0000        30.0000        50.0000
s1           log               factor      250.000        1.00000        5000.00
p1           log               factor     0.900000       0.100000       0.999000
o1           log               factor     1.000000E-05   1.000000E-10   1.000000E-02

Name         Group          Scale         Offset        Model command number
h1           pgnam         1.00000        0.00000            1
s1           pgnam         1.00000        0.00000            0
p1           pgnam         1.00000        0.00000            1
o1           pgnam         1.00000        0.00000            0


Prior information:-

   No prior information supplied


Observations:-

Observation name        Observation       Weight       Group
 o1                     2.590000E-08      1.000       conc
 o2                     6.041790E-08      1.000       conc
 o3                     7.045510E-08      1.000       conc
 o4                     6.061850E-08      1.000       conc
 o5                     4.035480E-08      1.000       conc
 o6                     1.840630E-08      1.000       conc
 o7                     2.862080E-09      1.000       conc
 o8                     1.398620E-09      1.000       conc
 o9                     1.242340E-08      1.000       conc
 o10                    2.511130E-08      1.000       conc
 o11                    2.840230E-08      1.000       conc
 o12                    1.623870E-08      1.000       conc
 o13                    1.391590E-09      1.000       conc
 o14                     0.00000          1.000       conc
 o15                    8.042150E-09      1.000       conc
 o16                    2.323690E-08      1.000       conc
 o17                    3.486020E-08      1.000       conc
 o18                    3.221440E-08      1.000       conc
 o19                    2.012160E-08      1.000       conc
 o20                    1.820490E-08      1.000       conc
 o21                    2.995640E-08      1.000       conc
 o22                    3.351660E-08      1.000       conc
 o23                    1.735370E-08      1.000       conc
 o24                     0.00000          1.000       conc
 o25                     0.00000          1.000       conc
 o26                    1.827050E-09      1.000       conc
 o27                     0.00000          1.000       conc
 o28                    2.878970E-09      1.000       conc
 o29                    2.847040E-08      1.000       conc
 o30                    5.884060E-08      1.000       conc
 o31                    5.537600E-08      1.000       conc
 o32                    2.838700E-08      1.000       conc
 o33                    3.754250E-08      1.000       conc
 o34                    9.696690E-08      1.000       conc
 o35                    1.449780E-07      1.000       conc
 o36                    1.388220E-07      1.000       conc
 o37                    1.008400E-07      1.000       conc
 o38                    6.353650E-08      1.000       conc
 o39                    5.595610E-08      1.000       conc
 o40                    6.928060E-08      1.000       conc
 o41                    7.249730E-08      1.000       conc
 o42                    5.923150E-08      1.000       conc
 o43                    4.597250E-08      1.000       conc
 o44                    4.046490E-08      1.000       conc
 o45                    4.299730E-08      1.000       conc
 o46                    4.403430E-08      1.000       conc
 o47                    4.280100E-08      1.000       conc
 o48                    5.510130E-08      1.000       conc
 o49                    7.098810E-08      1.000       conc
 o50                    6.257670E-08      1.000       conc
 o51                    3.340120E-08      1.000       conc
 o52                    2.130400E-09      1.000       conc
 o53                    1.315980E-08      1.000       conc
```

o54	1.742090E-07	1.000	conc
o55	6.601930E-07	1.000	conc
o56	1.501590E-06	1.000	conc
o57	2.390120E-06	1.000	conc
o58	3.116340E-06	1.000	conc
o59	4.010250E-06	1.000	conc
o60	5.504160E-06	1.000	conc
o61	7.889550E-06	1.000	conc
o62	1.136910E-05	1.000	conc
o63	1.576620E-05	1.000	conc
o64	2.067220E-05	1.000	conc
o65	2.567570E-05	1.000	conc
o66	3.036650E-05	1.000	conc
o67	3.434180E-05	1.000	conc
o68	3.721140E-05	1.000	conc
o69	3.871450E-05	1.000	conc
o70	3.889650E-05	1.000	conc
o71	3.799760E-05	1.000	conc
o72	3.684470E-05	1.000	conc
o73	3.620540E-05	1.000	conc
o74	3.552260E-05	1.000	conc
o75	3.381830E-05	1.000	conc
o76	3.141060E-05	1.000	conc
o77	2.941670E-05	1.000	conc
o78	2.819400E-05	1.000	conc
o79	2.731840E-05	1.000	conc
o80	2.593800E-05	1.000	conc
o81	2.274050E-05	1.000	conc
o82	1.876040E-05	1.000	conc
o83	1.644770E-05	1.000	conc
o84	1.546030E-05	1.000	conc
o85	1.447720E-05	1.000	conc
o86	1.325630E-05	1.000	conc
o87	1.213110E-05	1.000	conc
o88	1.106710E-05	1.000	conc
o89	1.003450E-05	1.000	conc
o90	9.242660E-06	1.000	conc
o91	8.848360E-06	1.000	conc
o92	8.809330E-06	1.000	conc
o93	8.093760E-06	1.000	conc
o94	7.046590E-06	1.000	conc
o95	7.096300E-06	1.000	conc
o96	6.642990E-06	1.000	conc
o97	6.279300E-06	1.000	conc
o98	6.008520E-06	1.000	conc
o99	5.694840E-06	1.000	conc
o100	5.320280E-06	1.000	conc
o101	4.943720E-06	1.000	conc
o102	4.579720E-06	1.000	conc
o103	4.272880E-06	1.000	conc
o104	4.106740E-06	1.000	conc
o105	3.955550E-06	1.000	conc
o106	3.762510E-06	1.000	conc
o107	3.487950E-06	1.000	conc
o108	3.157850E-06	1.000	conc
o109	3.073580E-06	1.000	conc
o110	3.123350E-06	1.000	conc
o111	2.972620E-06	1.000	conc
o112	2.396930E-06	1.000	conc
o113	2.161300E-06	1.000	conc
o114	2.331570E-06	1.000	conc
o115	2.195760E-06	1.000	conc
o116	2.013480E-06	1.000	conc
o117	1.992600E-06	1.000	conc
o118	2.067110E-06	1.000	conc
o119	2.024050E-06	1.000	conc
o120	1.793380E-06	1.000	conc
o121	1.735330E-06	1.000	conc
o122	1.782960E-06	1.000	conc
o123	1.637310E-06	1.000	conc
o124	1.542970E-06	1.000	conc
o125	1.594510E-06	1.000	conc
o126	1.661020E-06	1.000	conc

o127	1.640720E-06	1.000	conc
o128	1.554100E-06	1.000	conc
o129	1.449310E-06	1.000	conc
o130	1.372540E-06	1.000	conc
o131	1.335330E-06	1.000	conc
o132	1.319800E-06	1.000	conc
o133	1.307100E-06	1.000	conc
o134	1.279690E-06	1.000	conc
o135	1.235570E-06	1.000	conc
o136	1.183070E-06	1.000	conc
o137	1.130690E-06	1.000	conc
o138	1.085970E-06	1.000	conc
o139	1.054120E-06	1.000	conc
o140	1.040060E-06	1.000	conc
o141	1.047440E-06	1.000	conc
o142	1.067610E-06	1.000	conc
o143	1.084930E-06	1.000	conc
o144	1.083670E-06	1.000	conc
o145	1.051100E-06	1.000	conc
o146	9.960480E-07	1.000	conc
o147	9.367260E-07	1.000	conc
o148	8.912290E-07	1.000	conc
o149	8.673420E-07	1.000	conc
o150	8.570490E-07	1.000	conc
o151	8.509940E-07	1.000	conc
o152	8.410760E-07	1.000	conc
o153	8.267230E-07	1.000	conc
o154	8.101600E-07	1.000	conc
o155	7.936160E-07	1.000	conc
o156	7.787460E-07	1.000	conc
o157	7.646170E-07	1.000	conc
o158	7.495700E-07	1.000	conc
o159	7.320050E-07	1.000	conc
o160	7.117650E-07	1.000	conc
o161	6.901710E-07	1.000	conc
o162	6.686150E-07	1.000	conc
o163	6.488390E-07	1.000	conc
o164	6.339230E-07	1.000	conc
o165	6.272630E-07	1.000	conc
o166	6.322520E-07	1.000	conc
o167	6.483740E-07	1.000	conc
o168	6.640750E-07	1.000	conc
o169	6.717990E-07	1.000	conc
o170	6.722490E-07	1.000	conc
o171	6.667270E-07	1.000	conc
o172	6.565360E-07	1.000	conc
o173	6.429800E-07	1.000	conc
o174	6.273620E-07	1.000	conc
o175	6.109860E-07	1.000	conc
o176	5.948490E-07	1.000	conc
o177	5.787370E-07	1.000	conc
o178	5.621310E-07	1.000	conc
o179	5.445970E-07	1.000	conc
o180	5.274260E-07	1.000	conc
o181	5.135020E-07	1.000	conc
o182	5.057720E-07	1.000	conc
o183	5.056420E-07	1.000	conc
o184	5.093550E-07	1.000	conc
o185	5.120720E-07	1.000	conc
o186	5.089570E-07	1.000	conc
o187	4.979250E-07	1.000	conc
o188	4.838970E-07	1.000	conc
o189	4.728920E-07	1.000	conc
o190	4.702190E-07	1.000	conc
o191	4.733380E-07	1.000	conc
o192	4.748510E-07	1.000	conc
o193	4.673070E-07	1.000	conc
o194	4.478510E-07	1.000	conc
o195	4.235710E-07	1.000	conc
o196	4.028560E-07	1.000	conc
o197	3.940200E-07	1.000	conc
o198	3.997040E-07	1.000	conc
o199	4.130600E-07	1.000	conc

o200	4.263430E-07	1.000	conc
o201	4.326460E-07	1.000	conc
o202	4.306550E-07	1.000	conc
o203	4.213290E-07	1.000	conc
o204	4.056960E-07	1.000	conc
o205	3.878020E-07	1.000	conc
o206	3.760210E-07	1.000	conc
o207	3.790600E-07	1.000	conc
o208	4.048110E-07	1.000	conc
o209	4.380700E-07	1.000	conc
o210	4.505410E-07	1.000	conc
o211	4.448250E-07	1.000	conc
o212	4.285460E-07	1.000	conc
o213	4.093090E-07	1.000	conc
o214	3.923830E-07	1.000	conc
o215	3.785030E-07	1.000	conc
o216	3.678840E-07	1.000	conc
o217	3.606130E-07	1.000	conc
o218	3.557500E-07	1.000	conc
o219	3.518660E-07	1.000	conc
o220	3.475430E-07	1.000	conc
o221	3.421740E-07	1.000	conc
o222	3.365050E-07	1.000	conc
o223	3.314100E-07	1.000	conc
o224	3.277660E-07	1.000	conc
o225	3.266420E-07	1.000	conc
o226	3.293540E-07	1.000	conc
o227	3.372350E-07	1.000	conc
o228	3.503730E-07	1.000	conc
o229	3.627350E-07	1.000	conc
o230	3.664120E-07	1.000	conc
o231	3.538240E-07	1.000	conc
o232	3.267250E-07	1.000	conc
o233	2.972220E-07	1.000	conc
o234	2.779620E-07	1.000	conc
o235	2.807770E-07	1.000	conc
o236	3.027670E-07	1.000	conc
o237	3.282720E-07	1.000	conc
o238	3.412080E-07	1.000	conc
o239	3.313560E-07	1.000	conc
o240	3.067770E-07	1.000	conc
o241	2.790880E-07	1.000	conc
o242	2.593120E-07	1.000	conc
o243	2.500270E-07	1.000	conc
o244	2.475120E-07	1.000	conc
o245	2.478980E-07	1.000	conc
o246	2.476030E-07	1.000	conc
o247	2.459700E-07	1.000	conc
o248	2.440310E-07	1.000	conc
o249	2.428400E-07	1.000	conc
o250	2.429470E-07	1.000	conc
o251	2.438980E-07	1.000	conc
o252	2.451130E-07	1.000	conc
o253	2.460160E-07	1.000	conc
o254	2.460300E-07	1.000	conc
o255	2.445760E-07	1.000	conc
o256	2.410770E-07	1.000	conc
o257	2.349560E-07	1.000	conc
o258	2.256350E-07	1.000	conc
o259	2.127040E-07	1.000	conc
o260	1.975550E-07	1.000	conc
o261	1.826670E-07	1.000	conc
o262	1.705390E-07	1.000	conc
o263	1.636690E-07	1.000	conc
o264	1.644490E-07	1.000	conc
o265	1.726930E-07	1.000	conc
o266	1.855120E-07	1.000	conc
o267	1.998920E-07	1.000	conc
o268	2.128170E-07	1.000	conc
o269	2.212710E-07	1.000	conc
o270	2.227080E-07	1.000	conc
o271	2.176220E-07	1.000	conc
o272	2.077180E-07	1.000	conc

o273	1.947020E-07	1.000	conc
o274	1.802810E-07	1.000	conc
o275	1.661270E-07	1.000	conc
o276	1.534240E-07	1.000	conc
o277	1.430190E-07	1.000	conc
o278	1.357500E-07	1.000	conc
o279	1.324550E-07	1.000	conc
o280	1.339740E-07	1.000	conc
o281	1.405780E-07	1.000	conc
o282	1.502300E-07	1.000	conc
o283	1.603050E-07	1.000	conc
o284	1.681780E-07	1.000	conc
o285	1.712250E-07	1.000	conc
o286	1.672560E-07	1.000	conc
o287	1.574450E-07	1.000	conc
o288	1.445270E-07	1.000	conc
o289	1.312480E-07	1.000	conc
o290	1.203520E-07	1.000	conc
o291	1.145800E-07	1.000	conc
o292	1.155330E-07	1.000	conc
o293	1.218370E-07	1.000	conc
o294	1.316430E-07	1.000	conc
o295	1.431010E-07	1.000	conc
o296	1.543620E-07	1.000	conc
o297	1.637930E-07	1.000	conc
o298	1.708000E-07	1.000	conc
o299	1.751040E-07	1.000	conc
o300	1.764210E-07	1.000	conc
o301	1.744710E-07	1.000	conc
o302	1.689780E-07	1.000	conc
o303	1.601270E-07	1.000	conc
o304	1.488840E-07	1.000	conc
o305	1.362920E-07	1.000	conc
o306	1.233950E-07	1.000	conc
o307	1.112360E-07	1.000	conc
o308	1.008570E-07	1.000	conc
o309	9.328990E-08	1.000	conc
o310	8.893630E-08	1.000	conc
o311	8.728010E-08	1.000	conc
o312	8.773220E-08	1.000	conc
o313	8.970370E-08	1.000	conc
o314	9.260560E-08	1.000	conc
o315	9.584880E-08	1.000	conc
o316	9.884430E-08	1.000	conc
o317	1.011340E-07	1.000	conc
o318	1.028180E-07	1.000	conc
o319	1.041430E-07	1.000	conc
o320	1.053590E-07	1.000	conc
o321	1.067130E-07	1.000	conc
o322	1.084520E-07	1.000	conc
o323	1.108260E-07	1.000	conc
o324	1.140510E-07	1.000	conc
o325	1.178980E-07	1.000	conc
o326	1.217920E-07	1.000	conc
o327	1.251530E-07	1.000	conc
o328	1.273980E-07	1.000	conc
o329	1.279450E-07	1.000	conc
o330	1.262120E-07	1.000	conc
o331	1.216930E-07	1.000	conc
o332	1.147900E-07	1.000	conc
o333	1.064960E-07	1.000	conc
o334	9.781810E-08	1.000	conc
o335	8.976220E-08	1.000	conc
o336	8.333400E-08	1.000	conc
o337	7.953930E-08	1.000	conc
o338	7.937580E-08	1.000	conc
o339	8.310890E-08	1.000	conc
o340	8.970640E-08	1.000	conc
o341	9.800290E-08	1.000	conc
o342	1.068330E-07	1.000	conc
o343	1.150320E-07	1.000	conc
o344	1.214330E-07	1.000	conc
o345	1.248720E-07	1.000	conc

o346	1.244490E-07	1.000	conc
o347	1.206740E-07	1.000	conc
o348	1.145190E-07	1.000	conc
o349	1.069590E-07	1.000	conc
o350	9.896780E-08	1.000	conc
o351	9.151880E-08	1.000	conc
o352	8.558610E-08	1.000	conc
o353	8.210600E-08	1.000	conc
o354	8.126640E-08	1.000	conc
o355	8.256360E-08	1.000	conc
o356	8.546820E-08	1.000	conc
o357	8.945030E-08	1.000	conc
o358	9.398060E-08	1.000	conc
o359	9.852930E-08	1.000	conc
o360	1.025670E-07	1.000	conc
o361	1.056860E-07	1.000	conc
o362	1.077830E-07	1.000	conc
o363	1.088070E-07	1.000	conc
o364	1.087020E-07	1.000	conc
o365	1.074160E-07	1.000	conc
o366	1.048950E-07	1.000	conc
o367	1.010860E-07	1.000	conc
o368	9.611800E-08	1.000	conc
o369	9.050930E-08	1.000	conc
o370	8.482960E-08	1.000	conc
o371	7.964840E-08	1.000	conc
o372	7.553540E-08	1.000	conc
o373	7.306010E-08	1.000	conc
o374	7.279220E-08	1.000	conc
o375	7.514010E-08	1.000	conc
o376	7.944540E-08	1.000	conc
o377	8.461630E-08	1.000	conc
o378	8.955980E-08	1.000	conc
o379	9.318290E-08	1.000	conc
o380	9.439240E-08	1.000	conc
o381	9.209550E-08	1.000	conc
o382	8.524380E-08	1.000	conc
o383	7.405490E-08	1.000	conc
o384	6.015780E-08	1.000	conc
o385	4.525560E-08	1.000	conc
o386	3.105130E-08	1.000	conc
o387	1.924800E-08	1.000	conc
o388	1.154880E-08	1.000	conc
o389	9.269960E-09	1.000	conc
o390	1.192830E-08	1.000	conc
o391	1.851730E-08	1.000	conc
o392	2.803040E-08	1.000	conc
o393	3.946100E-08	1.000	conc
o394	5.180260E-08	1.000	conc
o395	6.404870E-08	1.000	conc
o396	7.522440E-08	1.000	conc
o397	8.488610E-08	1.000	conc
o398	9.302900E-08	1.000	conc
o399	9.966150E-08	1.000	conc
o400	1.047920E-07	1.000	conc
o401	1.084280E-07	1.000	conc
o402	1.105790E-07	1.000	conc
o403	1.112630E-07	1.000	conc
o404	1.106210E-07	1.000	conc
o405	1.088860E-07	1.000	conc
o406	1.062910E-07	1.000	conc
o407	1.030690E-07	1.000	conc
o408	9.945440E-08	1.000	conc
o409	9.568040E-08	1.000	conc
o410	9.197960E-08	1.000	conc
o411	8.849010E-08	1.000	conc
o412	8.516850E-08	1.000	conc
o413	8.195060E-08	1.000	conc
o414	7.877250E-08	1.000	conc
o415	7.557000E-08	1.000	conc
o416	7.227910E-08	1.000	conc
o417	6.883580E-08	1.000	conc
o418	6.520510E-08	1.000	conc

o419	6.152120E-08	1.000	conc
o420	5.797910E-08	1.000	conc
o421	5.477390E-08	1.000	conc
o422	5.210060E-08	1.000	conc
o423	5.015430E-08	1.000	conc
o424	4.913010E-08	1.000	conc
o425	4.914850E-08	1.000	conc
o426	4.996780E-08	1.000	conc
o427	5.123580E-08	1.000	conc
o428	5.260070E-08	1.000	conc
o429	5.372650E-08	1.000	conc
o430	5.452150E-08	1.000	conc
o431	5.508430E-08	1.000	conc
o432	5.551840E-08	1.000	conc
o433	5.592730E-08	1.000	conc
o434	5.641470E-08	1.000	conc
o435	5.708410E-08	1.000	conc
o436	5.803860E-08	1.000	conc
o437	5.931950E-08	1.000	conc
o438	6.083730E-08	1.000	conc
o439	6.248570E-08	1.000	conc
o440	6.415840E-08	1.000	conc
o441	6.574900E-08	1.000	conc
o442	6.715150E-08	1.000	conc
o443	6.825940E-08	1.000	conc
o444	6.897850E-08	1.000	conc
o445	6.929210E-08	1.000	conc
o446	6.921410E-08	1.000	conc
o447	6.875870E-08	1.000	conc
o448	6.793980E-08	1.000	conc
o449	6.677160E-08	1.000	conc
o450	6.526820E-08	1.000	conc
o451	6.346600E-08	1.000	conc
o452	6.152200E-08	1.000	conc
o453	5.963400E-08	1.000	conc
o454	5.799960E-08	1.000	conc
o455	5.681660E-08	1.000	conc
o456	5.628270E-08	1.000	conc
o457	5.659560E-08	1.000	conc
o458	5.793820E-08	1.000	conc
o459	6.018830E-08	1.000	conc
o460	6.293640E-08	1.000	conc
o461	6.576190E-08	1.000	conc
o462	6.824420E-08	1.000	conc
o463	6.996260E-08	1.000	conc
o464	7.049670E-08	1.000	conc
o465	6.942630E-08	1.000	conc
o466	6.658270E-08	1.000	conc
o467	6.244930E-08	1.000	conc
o468	5.761370E-08	1.000	conc
o469	5.266350E-08	1.000	conc
o470	4.818620E-08	1.000	conc
o471	4.476960E-08	1.000	conc
o472	4.300000E-08	1.000	conc
o473	4.321500E-08	1.000	conc
o474	4.520450E-08	1.000	conc
o475	4.868480E-08	1.000	conc
o476	5.337270E-08	1.000	conc
o477	5.898460E-08	1.000	conc
o478	6.523700E-08	1.000	conc
o479	7.184670E-08	1.000	conc
o480	7.851040E-08	1.000	conc
o481	8.479280E-08	1.000	conc
o482	9.020340E-08	1.000	conc
o483	9.425170E-08	1.000	conc
o484	9.644720E-08	1.000	conc
o485	9.629920E-08	1.000	conc
o486	9.331730E-08	1.000	conc
o487	8.703580E-08	1.000	conc
o488	7.771540E-08	1.000	conc
o489	6.644230E-08	1.000	conc
o490	5.434720E-08	1.000	conc
o491	4.256060E-08	1.000	conc

```
 o492                   3.221320E-08      1.000       conc
 o493                   2.443570E-08      1.000       conc
 o494                   2.032130E-08      1.000       conc
 o495                   2.013560E-08      1.000       conc
 o496                   2.333550E-08      1.000       conc
 o497                   2.934400E-08      1.000       conc
 o498                   3.758420E-08      1.000       conc
 o499                   4.747890E-08      1.000       conc
 o500                   5.845140E-08      1.000       conc


Control settings:-

   Initial lambda                                               :  10.000
   Lambda adjustment factor                                     :  2.0000
   Sufficient new/old phi ratio per optimisation iteration      : 0.30000
   Limiting relative phi reduction between lambdas              : 1.00000E-02
   Maximum trial lambdas per iteration                          :   8
   Forgive model run failure during lamda testing               : yes
   Forgive model run failure during Jacobian runs               : yes

   Perform Broyden's update of Jacobian matrix                  : no
   Undertake observation re-referencing                         : no

   Maximum  factor  parameter change (factor-limited changes)   :  10.000
   Maximum relative parameter change (relative-limited changes) : na
   Fraction of initial parameter values used in computing
   change limit for near-zero parameters                        : 1.00000E-03
   Allow bending of parameter upgrade vector                    : no
   Allow parameters to stick to their bounds                    : no

   Relative phi reduction below which to begin use of
   central derivatives                                          : 0.10000
   Iteration at which to first consider derivatives switch      :   1

   Relative phi reduction indicating convergence                : 0.50000E-02
   Number of phi values required within this range              :   4
   Maximum number of consecutive failures to lower phi          :   4
   Minimal relative parameter change indicating convergence     : 0.50000E-02
   Number of consecutive iterations with minimal param change   :   4
   Maximum number of optimisation iterations                    : 200

   Attempt automatic user intervention                          : no

   Attempt reuse of parameter sensitivities                     : no

   Scale parameters by their bounds                             : yes


File saving options: -

   Save best JCO file                                           : yes
   Save multiple JCO files                                      : no
   Save multiple REI files                                      : no
   Save multiple PAR files                                      : yes


                            OPTIMISATION RECORD


INITIAL CONDITIONS:
   Sum of squared weighted residuals (ie phi) =  3.40630E-09

      Current parameter values
      h1                 40.0000
      s1                 250.000
      p1                0.900000
      o1                1.000000E-05


OPTIMISATION ITERATION NO.        : 1
   Model calls so far             : 1
   Derivative model calls so far  : 0
```

```
   Starting phi for this iteration:  3.40630E-09

       Lambda =   10.000     ----->
          Phi =  3.25120E-09  (  0.954 of starting phi)

       Lambda =   5.0000     ----->
          Phi =  3.25120E-09  (  0.954 of starting phi)

   No more lambdas: relative phi reduction between lambdas less than 0.0100
   Lowest phi this iteration:  3.25120E-09
   Relative phi reduction between optimisation iterations less than 0.1000
   Switch to higher order derivatives calculation

      Current parameter values                 Previous parameter values
      h1                39.7726                h1                40.0000
      s1                243.318                s1                250.000
      p1               0.899875                p1               0.900000
      o1               1.004959E-05            o1               1.000000E-05
   Maximum   factor change:  1.027     ["s1"]
   Maximum relative change: 2.6727E-02 ["s1"]


OPTIMISATION ITERATION NO.        : 2
   Model calls so far             : 5
   Derivative model calls so far  : 1
   Starting phi for this iteration:  3.25120E-09

       Lambda =   5.0000     ----->
          Phi =  2.94127E-09  (  0.905 of starting phi)

       Lambda =   2.5000     ----->
          Phi =  2.94127E-09  (  0.905 of starting phi)

   No more lambdas: relative phi reduction between lambdas less than 0.0100
   Lowest phi this iteration:  2.94127E-09

      Current parameter values                 Previous parameter values
      h1                39.4163                h1                39.7726
      s1                222.592                s1                243.318
      p1               0.899693                p1               0.899875
      o1               1.018809E-05            o1               1.004959E-05
   Maximum   factor change:  1.093     ["s1"]
   Maximum relative change: 8.5183E-02 ["s1"]


OPTIMISATION ITERATION NO.        : 3
   Model calls so far             : 11
   Derivative model calls so far  : 2
   Starting phi for this iteration:  2.94127E-09

       Lambda =   2.5000     ----->
          Phi =  2.79694E-09  (  0.951 of starting phi)

       Lambda =   1.2500     ----->
          Phi =  2.79694E-09  (  0.951 of starting phi)

   No more lambdas: relative phi reduction between lambdas less than 0.0100
   Lowest phi this iteration:  2.79694E-09

      Current parameter values                 Previous parameter values
      h1                39.1908                h1                39.4163
      s1                205.050                s1                222.592
      p1               0.899564                p1               0.899693
      o1               1.033842E-05            o1               1.018809E-05
   Maximum   factor change:  1.086     ["s1"]
   Maximum relative change: 7.8805E-02 ["s1"]


OPTIMISATION ITERATION NO.        : 4
   Model calls so far             : 17
   Derivative model calls so far  : 3
   Starting phi for this iteration:  2.79694E-09
```

```
        Lambda =  0.62500     ----->
           Phi =  2.70642E-09  (  0.968 of starting phi)

        Lambda =  0.31250     ----->
           Phi =  2.70642E-09  (  0.968 of starting phi)

        Lambda =   1.2500     ----->
           Phi =  2.70642E-09  (  0.968 of starting phi)

    No more lambdas: phi rising
    Lowest phi this iteration:  2.70642E-09

       Current parameter values                 Previous parameter values
       h1                39.0354                h1                39.1908
       s1                189.361                s1                205.050
       p1               0.899465                p1               0.899564
       o1               1.050122E-05            o1               1.033842E-05
    Maximum   factor change:  1.083     ["s1"]
    Maximum relative change: 7.6514E-02 ["s1"]


 OPTIMISATION ITERATION NO.        : 5
    Model calls so far             : 24
    Derivative model calls so far  : 4
    Starting phi for this iteration:  2.70642E-09

        Lambda =  0.62500     ----->
           Phi =  2.64770E-09  (  0.978 of starting phi)

        Lambda =  0.31250     ----->
           Phi =  2.64770E-09  (  0.978 of starting phi)

    No more lambdas: relative phi reduction between lambdas less than 0.0100
    Lowest phi this iteration:  2.64770E-09

       Current parameter values                 Previous parameter values
       h1                38.9299                h1                39.0354
       s1                176.071                s1                189.361
       p1               0.899390                p1               0.899465
       o1               1.066639E-05            o1               1.050122E-05
    Maximum   factor change:  1.075     ["s1"]
    Maximum relative change: 7.0184E-02 ["s1"]


 OPTIMISATION ITERATION NO.        : 6
    Model calls so far             : 30
    Derivative model calls so far  : 5
    Starting phi for this iteration:  2.64770E-09

        Lambda =  0.15625     ----->
           Phi =  2.61368E-09  (  0.987 of starting phi)

        Lambda =  7.81250E-02 ----->
           Phi =  2.61368E-09  (  0.987 of starting phi)

        Lambda =  0.31250     ----->
           Phi =  2.61368E-09  (  0.987 of starting phi)

    No more lambdas: phi rising
    Lowest phi this iteration:  2.61368E-09

       Current parameter values                 Previous parameter values
       h1                38.8622                h1                38.9299
       s1                165.857                s1                176.071
       p1               0.899335                p1               0.899390
       o1               1.082255E-05            o1               1.066639E-05
    Maximum   factor change:  1.062     ["s1"]
    Maximum relative change: 5.8011E-02 ["s1"]


 OPTIMISATION ITERATION NO.        : 7
    Model calls so far             : 37
    Derivative model calls so far  : 6
```

```
   Starting phi for this iteration:  2.61368E-09

       Lambda =  0.15625     ----->
          Phi =  2.59279E-09  (  0.992 of starting phi)

       Lambda =  7.81250E-02 ----->
          Phi =  2.59279E-09  (  0.992 of starting phi)

   No more lambdas: relative phi reduction between lambdas less than 0.0100
   Lowest phi this iteration:  2.59279E-09

      Current parameter values                 Previous parameter values
      h1               38.8174                 h1                38.8622
      s1               157.901                 s1                165.857
      p1              0.899292                 p1               0.899335
      o1              1.098669E-05             o1               1.082255E-05
   Maximum   factor change:  1.050     ["s1"]
   Maximum relative change: 4.7970E-02 ["s1"]


OPTIMISATION ITERATION NO.        : 8
   Model calls so far             : 43
   Derivative model calls so far  : 7
   Starting phi for this iteration:  2.59279E-09

       Lambda =  3.90625E-02 ----->
          Phi =  2.53940E-09  (  0.979 of starting phi)

       Lambda =  1.95313E-02 ----->
          Phi =  2.53938E-09  (  0.979 of starting phi)

   No more lambdas: relative phi reduction between lambdas less than 0.0100
   Lowest phi this iteration:  2.53938E-09

      Current parameter values                 Previous parameter values
      h1               38.7740                 h1                38.8174
      s1               145.344                 s1                157.901
      p1              0.899212                 p1               0.899292
      o1              1.157940E-05             o1               1.098669E-05
   Maximum   factor change:  1.086     ["s1"]
   Maximum relative change: 7.9522E-02 ["s1"]


OPTIMISATION ITERATION NO.        : 9
   Model calls so far             : 49
   Derivative model calls so far  : 8
   Starting phi for this iteration:  2.53938E-09

       Lambda =  9.76563E-03 ----->
          Phi =  2.52278E-09  (  0.993 of starting phi)

       Lambda =  4.88281E-03 ----->
          Phi =  2.52277E-09  (  0.993 of starting phi)

   No more lambdas: relative phi reduction between lambdas less than 0.0100
   Lowest phi this iteration:  2.52277E-09

      Current parameter values                 Previous parameter values
      h1               38.8043                 h1                38.7740
      s1               150.296                 s1                145.344
      p1              0.899237                 p1               0.899212
      o1              1.156153E-05             o1               1.157940E-05
   Maximum   factor change:  1.034     ["s1"]
   Maximum relative change: 3.4066E-02 ["s1"]


OPTIMISATION ITERATION NO.        : 10
   Model calls so far             : 55
   Derivative model calls so far  : 9
   Starting phi for this iteration:  2.52277E-09

       Lambda =  2.44141E-03 ----->
          Phi =  2.49515E-09  (  0.989 of starting phi)
```

```
        Lambda =  1.22070E-03 ----->
           Phi =  2.49501E-09  (  0.989 of starting phi)

    No more lambdas: relative phi reduction between lambdas less than 0.0100
    Lowest phi this iteration:  2.49501E-09

       Current parameter values                 Previous parameter values
       h1               38.8655                 h1                38.8043
       s1               158.969                 s1                150.296
       p1              0.899274                 p1               0.899237
       o1              1.164314E-05             o1               1.156153E-05
    Maximum   factor change:  1.058     ["s1"]
    Maximum relative change: 5.7707E-02 ["s1"]


 OPTIMISATION ITERATION NO.        : 11
    Model calls so far             : 61
    Derivative model calls so far  : 10
    Starting phi for this iteration:  2.49501E-09

        Lambda =  6.10352E-04 ----->
           Phi =  2.47730E-09  (  0.993 of starting phi)

        Lambda =  3.05176E-04 ----->
           Phi =  2.47707E-09  (  0.993 of starting phi)

    No more lambdas: relative phi reduction between lambdas less than 0.0100
    Lowest phi this iteration:  2.47707E-09

       Current parameter values                 Previous parameter values
       h1               38.8467                 h1                38.8655
       s1               154.572                 s1                158.969
       p1              0.899245                 p1               0.899274
       o1              1.181276E-05             o1               1.164314E-05
    Maximum   factor change:  1.028     ["s1"]
    Maximum relative change: 2.7662E-02 ["s1"]


 OPTIMISATION ITERATION NO.        : 12
    Model calls so far             : 67
    Derivative model calls so far  : 11
    Starting phi for this iteration:  2.47707E-09

        Lambda =  1.52588E-04 ----->
           Phi =  3.61906E-10  (  0.146 of starting phi)

    No more lambdas: phi is less than 0.3000 of starting phi
    Lowest phi this iteration:  3.61906E-10

       Current parameter values                 Previous parameter values
       h1               40.3253                 h1                38.8467
       s1               140.675                 s1                154.572
       p1              0.898122                 p1               0.899245
       o1              8.452134E-05             o1               1.181276E-05
    Maximum   factor change:  7.155     ["o1"]
    Maximum relative change:  6.155     ["o1"]


 OPTIMISATION ITERATION NO.        : 13
    Model calls so far             : 72
    Derivative model calls so far  : 12
    Starting phi for this iteration:  3.61906E-10

        Lambda =  7.62939E-05 ----->
           Phi =  3.38172E-10  (  0.934 of starting phi)

        Lambda =  3.81470E-05 ----->
           Phi =  3.38165E-10  (  0.934 of starting phi)

    No more lambdas: relative phi reduction between lambdas less than 0.0100
    Lowest phi this iteration:  3.38165E-10
```

```
        Current parameter values                 Previous parameter values
        h1               40.3008                 h1                40.3253
        s1               147.929                 s1                140.675
        p1              0.899294                 p1               0.898122
        o1              7.705132E-05             o1               8.452134E-05
     Maximum   factor change:  1.097     ["o1"]
     Maximum relative change: 8.8380E-02 ["o1"]


  OPTIMISATION ITERATION NO.        : 14
     Model calls so far             : 78
     Derivative model calls so far  : 13
     Starting phi for this iteration:  3.38165E-10

         Lambda =  1.90735E-05 ----->
            Phi =  3.37717E-10  (  0.999 of starting phi)

         Lambda =  9.53674E-06 ----->
            Phi =  3.37654E-10  (  0.998 of starting phi)

     No more lambdas: relative phi reduction between lambdas less than 0.0100
     Lowest phi this iteration:  3.37654E-10

        Current parameter values                 Previous parameter values
        h1               40.2981                 h1                40.3008
        s1               149.204                 s1                147.929
        p1              0.899070                 p1               0.899294
        o1              7.614777E-05             o1               7.705132E-05
     Maximum   factor change:  1.012     ["o1"]
     Maximum relative change: 1.1727E-02 ["o1"]


  OPTIMISATION ITERATION NO.        : 15
     Model calls so far             : 84
     Derivative model calls so far  : 14
     Starting phi for this iteration:  3.37654E-10

         Lambda =  4.76837E-06 ----->
            Phi =  3.37204E-10  (  0.999 of starting phi)

         Lambda =  2.38419E-06 ----->
            Phi =  3.37051E-10  (  0.998 of starting phi)

     No more lambdas: relative phi reduction between lambdas less than 0.0100
     Lowest phi this iteration:  3.37051E-10

        Current parameter values                 Previous parameter values
        h1               40.2984                 h1                40.2981
        s1               148.998                 s1                149.204
        p1              0.898303                 p1               0.899070
        o1              7.659821E-05             o1               7.614777E-05
     Maximum   factor change:  1.006     ["o1"]
     Maximum relative change: 5.9154E-03 ["o1"]


  OPTIMISATION ITERATION NO.        : 16
     Model calls so far             : 90
     Derivative model calls so far  : 15
     Starting phi for this iteration:  3.37051E-10

         Lambda =  1.19209E-06 ----->
            Phi =  3.25891E-10  (  0.967 of starting phi)

         Lambda =  5.96046E-07 ----->
            Phi =  3.25698E-10  (  0.966 of starting phi)

     No more lambdas: relative phi reduction between lambdas less than 0.0100
     Lowest phi this iteration:  3.25698E-10

        Current parameter values                 Previous parameter values
        h1               40.2808                 h1                40.2984
        s1               154.458                 s1                148.998
        p1              0.872214                 p1               0.898303
```

```
      o1               8.324239E-05             o1                7.659821E-05
   Maximum   factor change:  1.087     ["o1"]
   Maximum relative change: 8.6741E-02 ["o1"]


OPTIMISATION ITERATION NO.        : 17
   Model calls so far             : 96
   Derivative model calls so far  : 16
   Starting phi for this iteration:  3.25698E-10

       Lambda =  2.98023E-07 ----->
          Phi =  2.75008E-10  (  0.844 of starting phi)

       Lambda =  1.49012E-07 ----->
          Phi =  2.70922E-10  (  0.832 of starting phi)

       Lambda =  7.45058E-08 ----->
          Phi =  2.68367E-10  (  0.824 of starting phi)

   No more lambdas: relative phi reduction between lambdas less than 0.0100
   Lowest phi this iteration:  2.68367E-10

      Current parameter values                 Previous parameter values
      h1                 39.9957                h1                  40.2808
      s1                 176.381                s1                  154.458
      p1                0.844869                p1                 0.872214
      o1               1.200756E-04             o1                8.324239E-05
   Maximum   factor change:  1.442     ["o1"]
   Maximum relative change: 0.4425     ["o1"]


OPTIMISATION ITERATION NO.        : 18
   Model calls so far             : 103
   Derivative model calls so far  : 17
   Starting phi for this iteration:  2.68367E-10

       Lambda =  3.72529E-08 ----->
          Phi =  2.57827E-10  (  0.961 of starting phi)

       Lambda =  1.86265E-08 ----->
          Phi =  2.53019E-10  (  0.943 of starting phi)

       Lambda =  9.31323E-09 ----->
          Phi =  2.42539E-10  (  0.904 of starting phi)

       Lambda =  4.65661E-09 ----->
          Phi =  2.20859E-10  (  0.823 of starting phi)

       Lambda =  2.32831E-09 ----->
          Phi =  1.97463E-10  (  0.736 of starting phi)

       Lambda =  1.16415E-09 ----->
          Phi =  2.07024E-10  (  0.771 of starting phi)

   No more lambdas: phi rising
   Lowest phi this iteration:  1.97463E-10

      Current parameter values                 Previous parameter values
      h1                 39.7282                h1                  39.9957
      s1                 184.139                s1                  176.381
      p1                0.902757                p1                 0.844869
      o1               1.143853E-04             o1                1.200756E-04
   Maximum   factor change:  1.069     ["p1"]
   Maximum relative change: 6.8517E-02 ["p1"]


OPTIMISATION ITERATION NO.        : 19
   Model calls so far             : 113
   Derivative model calls so far  : 18
   Starting phi for this iteration:  1.97463E-10

       Lambda =  1.00000E-08 ----->
          Phi =  1.74044E-10  (  0.881 of starting phi)
```

```
        Lambda =  5.00000E-09 ----->
           Phi =  1.68618E-10  (  0.854 of starting phi)

        Lambda =  2.50000E-09 ----->
           Phi =  1.59917E-10  (  0.810 of starting phi)

        Lambda =  1.25000E-09 ----->
           Phi =  1.49475E-10  (  0.757 of starting phi)

        Lambda =  6.25000E-10 ----->
           Phi =  1.40664E-10  (  0.712 of starting phi)

        Lambda =  3.12500E-10 ----->
           Phi =  1.34087E-10  (  0.679 of starting phi)

        Lambda =  1.56250E-10 ----->
           Phi =  1.29749E-10  (  0.657 of starting phi)

        Lambda =  7.81250E-11 ----->
           Phi =  1.27359E-10  (  0.645 of starting phi)

    No more lambdas: allowed lambdas per iteration =  8
    Lowest phi this iteration:  1.27359E-10

       Current parameter values                 Previous parameter values
       h1               39.2239                 h1               39.7282
       s1               216.009                 s1               184.139
       p1              0.895856                 p1              0.902757
       o1              2.039755E-04             o1              1.143853E-04
    Maximum   factor change:  1.783     ["o1"]
    Maximum relative change: 0.7832     ["o1"]


 OPTIMISATION ITERATION NO.        : 20
    Model calls so far             : 125
    Derivative model calls so far  : 19
    Starting phi for this iteration:  1.27359E-10

        Lambda =  1.00000E-08 ----->
           Phi =  1.05456E-10  (  0.828 of starting phi)

        Lambda =  5.00000E-09 ----->
           Phi =  1.04864E-10  (  0.823 of starting phi)

    No more lambdas: relative phi reduction between lambdas less than 0.0100
    Lowest phi this iteration:  1.04864E-10

       Current parameter values                 Previous parameter values
       h1               39.1801                 h1               39.2239
       s1               220.772                 s1               216.009
       p1              0.892353                 p1              0.895856
       o1              1.888348E-04             o1              2.039755E-04
    Maximum   factor change:  1.080     ["o1"]
    Maximum relative change: 7.4228E-02 ["o1"]


 OPTIMISATION ITERATION NO.        : 21
    Model calls so far             : 131
    Derivative model calls so far  : 20
    Starting phi for this iteration:  1.04864E-10

        Lambda =  1.00000E-08 ----->
           Phi =  1.03656E-10  (  0.988 of starting phi)

        Lambda =  5.00000E-09 ----->
           Phi =  1.02553E-10  (  0.978 of starting phi)

        Lambda =  2.50000E-09 ----->
           Phi =  1.01033E-10  (  0.963 of starting phi)

        Lambda =  1.25000E-09 ----->
           Phi =  1.00472E-10  (  0.958 of starting phi)
```

```
   No more lambdas: relative phi reduction between lambdas less than 0.0100
   Lowest phi this iteration:  1.00472E-10

      Current parameter values                 Previous parameter values
      h1                39.1014                h1                 39.1801
      s1                226.701                s1                 220.772
      p1               0.889807                p1                0.892353
      o1               1.962592E-04            o1                1.888348E-04
   Maximum   factor change:  1.039     ["o1"]
   Maximum relative change: 3.9317E-02 ["o1"]


OPTIMISATION ITERATION NO.        : 22
   Model calls so far             : 139
   Derivative model calls so far  : 21
   Starting phi for this iteration:  1.00472E-10

       Lambda =  1.00000E-08 ----->
          Phi =  9.87595E-11  (  0.983 of starting phi)

       Lambda =  5.00000E-09 ----->
          Phi =  9.71659E-11  (  0.967 of starting phi)

       Lambda =  2.50000E-09 ----->
          Phi =  9.54897E-11  (  0.950 of starting phi)

       Lambda =  1.25000E-09 ----->
          Phi =  9.45401E-11  (  0.941 of starting phi)

   No more lambdas: relative phi reduction between lambdas less than 0.0100
   Lowest phi this iteration:  9.45401E-11

      Current parameter values                 Previous parameter values
      h1                38.9139                h1                 39.1014
      s1                241.801                s1                 226.701
      p1               0.889801                p1                0.889807
      o1               2.213909E-04            o1                1.962592E-04
   Maximum   factor change:  1.128     ["o1"]
   Maximum relative change: 0.1281     ["o1"]


OPTIMISATION ITERATION NO.        : 23
   Model calls so far             : 147
   Derivative model calls so far  : 22
   Starting phi for this iteration:  9.45401E-11

       Lambda =  1.00000E-08 ----->
          Phi =  9.41861E-11  (  0.996 of starting phi)

       Lambda =  5.00000E-09 ----->
          Phi =  9.41302E-11  (  0.996 of starting phi)

   No more lambdas: relative phi reduction between lambdas less than 0.0100
   Lowest phi this iteration:  9.41302E-11

      Current parameter values                 Previous parameter values
      h1                38.9023                h1                 38.9139
      s1                242.067                s1                 241.801
      p1               0.888611                p1                0.889801
      o1               2.205266E-04            o1                2.213909E-04
   Maximum   factor change:  1.004     ["o1"]
   Maximum relative change: 3.9037E-03 ["o1"]


OPTIMISATION ITERATION NO.        : 24
   Model calls so far             : 153
   Derivative model calls so far  : 23
   Starting phi for this iteration:  9.41302E-11

       Lambda =  1.00000E-08 ----->
          Phi =  9.40915E-11  (  1.000 of starting phi)
```

```
         Lambda =  5.00000E-09 ----->
            Phi =  9.40833E-11  (  1.000 of starting phi)

     No more lambdas: relative phi reduction between lambdas less than 0.0100
     Lowest phi this iteration:  9.40833E-11

        Current parameter values                 Previous parameter values
        h1                38.9005                h1                38.9023
        s1                242.330                s1                242.067
        p1               0.888559                p1               0.888611
        o1               2.209583E-04            o1               2.205266E-04
     Maximum   factor change:  1.002     ["o1"]
     Maximum relative change: 1.9576E-03 ["o1"]


  OPTIMISATION ITERATION NO.        : 25
     Model calls so far             : 159
     Derivative model calls so far  : 24
     Starting phi for this iteration:  9.40833E-11

         Lambda =  1.00000E-08 ----->
            Phi =  9.37546E-11  (  0.997 of starting phi)

         Lambda =  5.00000E-09 ----->
            Phi =  9.34593E-11  (  0.993 of starting phi)

     No more lambdas: relative phi reduction between lambdas less than 0.0100
     Lowest phi this iteration:  9.34593E-11

        Current parameter values                 Previous parameter values
        h1                38.8504                h1                38.9005
        s1                247.573                s1                242.330
        p1               0.887755                p1               0.888559
        o1               2.295780E-04            o1               2.209583E-04
     Maximum   factor change:  1.039     ["o1"]
     Maximum relative change: 3.9010E-02 ["o1"]


  OPTIMISATION ITERATION NO.        : 26
     Model calls so far             : 165
     Derivative model calls so far  : 25
     Starting phi for this iteration:  9.34593E-11

         Lambda =  1.00000E-08 ----->
            Phi =  9.33097E-11  (  0.998 of starting phi)

         Lambda =  5.00000E-09 ----->
            Phi =  9.32984E-11  (  0.998 of starting phi)

     No more lambdas: relative phi reduction between lambdas less than 0.0100
     Lowest phi this iteration:  9.32984E-11

        Current parameter values                 Previous parameter values
        h1                38.8403                h1                38.8504
        s1                247.212                s1                247.573
        p1               0.888011                p1               0.887755
        o1               2.287691E-04            o1               2.295780E-04
     Maximum   factor change:  1.004     ["o1"]
     Maximum relative change: 3.5232E-03 ["o1"]


  OPTIMISATION ITERATION NO.        : 27
     Model calls so far             : 171
     Derivative model calls so far  : 26
     Starting phi for this iteration:  9.32984E-11

         Lambda =  1.00000E-08 ----->
            Phi =  9.32700E-11  (  1.000 of starting phi)

         Lambda =  5.00000E-09 ----->
            Phi =  9.32698E-11  (  1.000 of starting phi)

     No more lambdas: relative phi reduction between lambdas less than 0.0100
```

```
   Lowest phi this iteration:  9.32698E-11

      Current parameter values                  Previous parameter values
      h1                38.8376                 h1                  38.8403
      s1                247.549                 s1                  247.212
      p1               0.887958                 p1                 0.888011
      o1               2.291732E-04             o1                 2.287691E-04
   Maximum   factor change:  1.002     ["o1"]
   Maximum relative change: 1.7663E-03 ["o1"]


OPTIMISATION ITERATION NO.        : 28
   Model calls so far             : 177
   Derivative model calls so far  : 27
   Starting phi for this iteration:  9.32698E-11

       Lambda =  1.00000E-08 ----->
          Phi =  9.30904E-11  (  0.998 of starting phi)

       Lambda =  5.00000E-09 ----->
          Phi =  9.30452E-11  (  0.998 of starting phi)

   No more lambdas: relative phi reduction between lambdas less than 0.0100
   Lowest phi this iteration:  9.30452E-11

      Current parameter values                  Previous parameter values
      h1                38.7989                 h1                  38.8376
      s1                250.144                 s1                  247.549
      p1               0.887726                 p1                 0.887958
      o1               2.339331E-04             o1                 2.291732E-04
   Maximum   factor change:  1.021     ["o1"]
   Maximum relative change: 2.0770E-02 ["o1"]

   Optimisation complete: the  4 lowest phi's are within a relative distance
                           of eachother of 5.000E-03
   Total model calls:    183

   The model has been run one final time using best parameters.
   Thus all model input files contain best parameter values, and model
   output files contain model results based on these parameters.


                            OPTIMISATION RESULTS

Covariance matrix and parameter confidence intervals cannot be determined:-
Memory conservation is operative so that covariance matrix is not calculated.


Parameters ----->

Parameter      Estimated value
 h1              38.7989
 s1              250.144
 p1             0.887726
 o1             2.339331E-04

See file mfit.sen for parameter sensitivities.


Observations ----->

Observation             Measured       Calculated     Residual       Weight     Group
                        value          value
o1                     2.590000E-08    0.00000       2.590000E-08    1.000
conc
o2                     6.041790E-08    0.00000       6.041790E-08    1.000
conc
o3                     7.045510E-08    0.00000       7.045510E-08    1.000
conc
o4                     6.061850E-08    0.00000       6.061850E-08    1.000
conc
o5                     4.035480E-08    0.00000       4.035480E-08    1.000
conc
```

1383	o6	1.840630E-08	0.00000	1.840630E-08	1.000
	conc				
1384	o7	2.862080E-09	6.548592-269	2.862080E-09	1.000
	conc				
1385	o8	1.398620E-09	4.838414-224	1.398620E-09	1.000
	conc				
1386	o9	1.242340E-08	1.685134-190	1.242340E-08	1.000
	conc				
1387	o10	2.511130E-08	1.654966-164	2.511130E-08	1.000
	conc				
1388	o11	2.840230E-08	8.453034-144	2.840230E-08	1.000
	conc				
1389	o12	1.623870E-08	6.209271-127	1.623870E-08	1.000
	conc				
1390	o13	1.391590E-09	5.996795-113	1.391590E-09	1.000
	conc				
1391	o14	0.00000	3.522993-101	-3.522993-101	1.000
	conc				
1392	o15	8.042150E-09	3.759690E-91	8.042150E-09	1.000
	conc				
1393	o16	2.323690E-08	1.625667E-82	2.323690E-08	1.000
	conc				
1394	o17	3.486020E-08	5.197733E-75	3.486020E-08	1.000
	conc				
1395	o18	3.221440E-08	1.946555E-68	3.221440E-08	1.000
	conc				
1396	o19	2.012160E-08	1.221087E-62	2.012160E-08	1.000
	conc				
1397	o20	1.820490E-08	1.701727E-57	1.820490E-08	1.000
	conc				
1398	o21	2.995640E-08	6.603809E-53	2.995640E-08	1.000
	conc				
1399	o22	3.351660E-08	8.567701E-49	3.351660E-08	1.000
	conc				
1400	o23	1.735370E-08	4.315774E-45	1.735370E-08	1.000
	conc				
1401	o24	0.00000	9.550668E-42	-9.550668E-42	1.000
	conc				
1402	o25	0.00000	1.029196E-38	-1.029196E-38	1.000
	conc				
1403	o26	1.827050E-09	5.888479E-36	1.827050E-09	1.000
	conc				
1404	o27	0.00000	1.924506E-33	-1.924506E-33	1.000
	conc				
1405	o28	2.878970E-09	3.823907E-31	2.878970E-09	1.000
	conc				
1406	o29	2.847040E-08	4.872594E-29	2.847040E-08	1.000
	conc				
1407	o30	5.884060E-08	4.169364E-27	5.884060E-08	1.000
	conc				
1408	o31	5.537600E-08	2.493219E-25	5.537600E-08	1.000
	conc				
1409	o32	2.838700E-08	1.078747E-23	2.838700E-08	1.000
	conc				
1410	o33	3.754250E-08	3.481354E-22	3.754250E-08	1.000
	conc				
1411	o34	9.696690E-08	8.606872E-21	9.696690E-08	1.000
	conc				
1412	o35	1.449780E-07	1.668957E-19	1.449780E-07	1.000
	conc				
1413	o36	1.388220E-07	2.591861E-18	1.388220E-07	1.000
	conc				
1414	o37	1.008400E-07	3.283983E-17	1.008400E-07	1.000
	conc				
1415	o38	6.353650E-08	3.451421E-16	6.353650E-08	1.000
	conc				
1416	o39	5.595610E-08	3.053735E-15	5.595610E-08	1.000
	conc				
1417	o40	6.928060E-08	2.304996E-14	6.928058E-08	1.000
	conc				
1418	o41	7.249730E-08	1.502128E-13	7.249715E-08	1.000
	conc				
1419	o42	5.923150E-08	8.543327E-13	5.923065E-08	1.000

						conc
1420	o43	4.597250E-08	4.282238E-12	4.596822E-08	1.000	conc
1421	o44	4.046490E-08	1.908465E-11	4.044582E-08	1.000	conc
1422	o45	4.299730E-08	7.623672E-11	4.292106E-08	1.000	conc
1423	o46	4.403430E-08	2.749779E-10	4.375932E-08	1.000	conc
1424	o47	4.280100E-08	9.015621E-10	4.189944E-08	1.000	conc
1425	o48	5.510130E-08	2.703473E-09	5.239783E-08	1.000	conc
1426	o49	7.098810E-08	7.456249E-09	6.353185E-08	1.000	conc
1427	o50	6.257670E-08	1.901238E-08	4.356432E-08	1.000	conc
1428	o51	3.340120E-08	4.503375E-08	-1.163255E-08	1.000	conc
1429	o52	2.130400E-09	9.952524E-08	-9.739484E-08	1.000	conc
1430	o53	1.315980E-08	2.060562E-07	-1.928964E-07	1.000	conc
1431	o54	1.742090E-07	4.011705E-07	-2.269615E-07	1.000	conc
1432	o55	6.601930E-07	7.370206E-07	-7.682758E-08	1.000	conc
1433	o56	1.501590E-06	1.281881E-06	2.197095E-07	1.000	conc
1434	o57	2.390120E-06	2.117128E-06	2.729919E-07	1.000	conc
1435	o58	3.116340E-06	3.329709E-06	-2.133686E-07	1.000	conc
1436	o59	4.010250E-06	5.000056E-06	-9.898055E-07	1.000	conc
1437	o60	5.504160E-06	7.186757E-06	-1.682597E-06	1.000	conc
1438	o61	7.889550E-06	9.910560E-06	-2.021010E-06	1.000	conc
1439	o62	1.136910E-05	1.314113E-05	-1.772029E-06	1.000	conc
1440	o63	1.576620E-05	1.678997E-05	-1.023769E-06	1.000	conc
1441	o64	2.067220E-05	2.071196E-05	-3.976305E-08	1.000	conc
1442	o65	2.567570E-05	2.471626E-05	9.594354E-07	1.000	conc
1443	o66	3.036650E-05	2.858524E-05	1.781259E-06	1.000	conc
1444	o67	3.434180E-05	3.209842E-05	2.243381E-06	1.000	conc
1445	o68	3.721140E-05	3.505732E-05	2.154080E-06	1.000	conc
1446	o69	3.871450E-05	3.730699E-05	1.407509E-06	1.000	conc
1447	o70	3.889650E-05	3.875090E-05	1.456021E-07	1.000	conc
1448	o71	3.799760E-05	3.935732E-05	-1.359722E-06	1.000	conc
1449	o72	3.684470E-05	3.915710E-05	-2.312397E-06	1.000	conc
1450	o73	3.620540E-05	3.823404E-05	-2.028644E-06	1.000	conc
1451	o74	3.552260E-05	3.671048E-05	-1.187880E-06	1.000	conc
1452	o75	3.381830E-05	3.473057E-05	-9.122716E-07	1.000	conc
1453	o76	3.141060E-05	3.244415E-05	-1.033547E-06	1.000	conc
1454	o77	2.941670E-05	2.999296E-05	-5.762602E-07	1.000	conc
1455	o78	2.819400E-05	2.750064E-05	6.933642E-07	1.000	conc

1456	o79 conc	2.731840E-05	2.506668E-05	2.251722E-06	1.000
1457	o80 conc	2.593800E-05	2.276428E-05	3.173717E-06	1.000
1458	o81 conc	2.274050E-05	2.064124E-05	2.099259E-06	1.000
1459	o82 conc	1.876040E-05	1.872298E-05	3.741547E-08	1.000
1460	o83 conc	1.644770E-05	1.701686E-05	-5.691555E-07	1.000
1461	o84 conc	1.546030E-05	1.551677E-05	-5.646812E-08	1.000
1462	o85 conc	1.447720E-05	1.420765E-05	2.695465E-07	1.000
1463	o86 conc	1.325630E-05	1.306930E-05	1.870023E-07	1.000
1464	o87 conc	1.213110E-05	1.207938E-05	5.171879E-08	1.000
1465	o88 conc	1.106710E-05	1.121569E-05	-1.485857E-07	1.000
1466	o89 conc	1.003450E-05	1.045754E-05	-4.230392E-07	1.000
1467	o90 conc	9.242660E-06	9.786630E-06	-5.439704E-07	1.000
1468	o91 conc	8.848360E-06	9.187344E-06	-3.389836E-07	1.000
1469	o92 conc	8.809330E-06	8.646759E-06	1.625709E-07	1.000
1470	o93 conc	8.093760E-06	8.154447E-06	-6.068678E-08	1.000
1471	o94 conc	7.046590E-06	7.702148E-06	-6.555582E-07	1.000
1472	o95 conc	7.096300E-06	7.283421E-06	-1.871211E-07	1.000
1473	o96 conc	6.642990E-06	6.893290E-06	-2.502997E-07	1.000
1474	o97 conc	6.279300E-06	6.527930E-06	-2.486300E-07	1.000
1475	o98 conc	6.008520E-06	6.184401E-06	-1.758808E-07	1.000
1476	o99 conc	5.694840E-06	5.860425E-06	-1.655847E-07	1.000
1477	o100 conc	5.320280E-06	5.554215E-06	-2.339350E-07	1.000
1478	o101 conc	4.943720E-06	5.264344E-06	-3.206235E-07	1.000
1479	o102 conc	4.579720E-06	4.989642E-06	-4.099220E-07	1.000
1480	o103 conc	4.272880E-06	4.729130E-06	-4.562495E-07	1.000
1481	o104 conc	4.106740E-06	4.481961E-06	-3.752213E-07	1.000
1482	o105 conc	3.955550E-06	4.247392E-06	-2.918418E-07	1.000
1483	o106 conc	3.762510E-06	4.024749E-06	-2.622394E-07	1.000
1484	o107 conc	3.487950E-06	3.813419E-06	-3.254692E-07	1.000
1485	o108 conc	3.157850E-06	3.612831E-06	-4.549806E-07	1.000
1486	o109 conc	3.073580E-06	3.422449E-06	-3.488694E-07	1.000
1487	o110 conc	3.123350E-06	3.241772E-06	-1.184221E-07	1.000
1488	o111 conc	2.972620E-06	3.070322E-06	-9.770209E-08	1.000
1489	o112 conc	2.396930E-06	2.907647E-06	-5.107169E-07	1.000
1490	o113 conc	2.161300E-06	2.753316E-06	-5.920160E-07	1.000
1491	o114 conc	2.331570E-06	2.606919E-06	-2.753495E-07	1.000
1492	o115	2.195760E-06	2.468067E-06	-2.723067E-07	1.000

						conc
1493	o116	2.013480E-06	2.336385E-06	-3.229053E-07	1.000	conc
1494	o117	1.992600E-06	2.211520E-06	-2.189202E-07	1.000	conc
1495	o118	2.067110E-06	2.093133E-06	-2.602300E-08	1.000	conc
1496	o119	2.024050E-06	1.980901E-06	4.314887E-08	1.000	conc
1497	o120	1.793380E-06	1.874517E-06	-8.113713E-08	1.000	conc
1498	o121	1.735330E-06	1.773688E-06	-3.835802E-08	1.000	conc
1499	o122	1.782960E-06	1.678135E-06	1.048253E-07	1.000	conc
1500	o123	1.637310E-06	1.587591E-06	4.971872E-08	1.000	conc
1501	o124	1.542970E-06	1.501805E-06	4.116536E-08	1.000	conc
1502	o125	1.594510E-06	1.420534E-06	1.739763E-07	1.000	conc
1503	o126	1.661020E-06	1.343549E-06	3.174710E-07	1.000	conc
1504	o127	1.640720E-06	1.270632E-06	3.700878E-07	1.000	conc
1505	o128	1.554100E-06	1.201576E-06	3.525245E-07	1.000	conc
1506	o129	1.449310E-06	1.136181E-06	3.131287E-07	1.000	conc
1507	o130	1.372540E-06	1.074261E-06	2.982786E-07	1.000	conc
1508	o131	1.335330E-06	1.015637E-06	3.196928E-07	1.000	conc
1509	o132	1.319800E-06	9.601386E-07	3.596614E-07	1.000	conc
1510	o133	1.307100E-06	9.076039E-07	3.994961E-07	1.000	conc
1511	o134	1.279690E-06	8.578797E-07	4.218103E-07	1.000	conc
1512	o135	1.235570E-06	8.108199E-07	4.247501E-07	1.000	conc
1513	o136	1.183070E-06	7.662859E-07	4.167841E-07	1.000	conc
1514	o137	1.130690E-06	7.241458E-07	4.065442E-07	1.000	conc
1515	o138	1.085970E-06	6.842746E-07	4.016954E-07	1.000	conc
1516	o139	1.054120E-06	6.465534E-07	4.075666E-07	1.000	conc
1517	o140	1.040060E-06	6.108693E-07	4.291907E-07	1.000	conc
1518	o141	1.047440E-06	5.771152E-07	4.703248E-07	1.000	conc
1519	o142	1.067610E-06	5.451895E-07	5.224205E-07	1.000	conc
1520	o143	1.084930E-06	5.149954E-07	5.699346E-07	1.000	conc
1521	o144	1.083670E-06	4.864416E-07	5.972284E-07	1.000	conc
1522	o145	1.051100E-06	4.594410E-07	5.916590E-07	1.000	conc
1523	o146	9.960480E-07	4.339112E-07	5.621368E-07	1.000	conc
1524	o147	9.367260E-07	4.097739E-07	5.269521E-07	1.000	conc
1525	o148	8.912290E-07	3.869550E-07	5.042740E-07	1.000	conc
1526	o149	8.673420E-07	3.653841E-07	5.019579E-07	1.000	conc
1527	o150	8.570490E-07	3.449944E-07	5.120546E-07	1.000	conc
1528	o151	8.509940E-07	3.257228E-07	5.252712E-07	1.000	conc

1529	o152	8.410760E-07	3.075093E-07	5.335667E-07	1.000	conc
1530	o153	8.267230E-07	2.902969E-07	5.364261E-07	1.000	conc
1531	o154	8.101600E-07	2.740319E-07	5.361281E-07	1.000	conc
1532	o155	7.936160E-07	2.586632E-07	5.349528E-07	1.000	conc
1533	o156	7.787460E-07	2.441424E-07	5.346036E-07	1.000	conc
1534	o157	7.646170E-07	2.304237E-07	5.341933E-07	1.000	conc
1535	o158	7.495700E-07	2.174636E-07	5.321064E-07	1.000	conc
1536	o159	7.320050E-07	2.052210E-07	5.267840E-07	1.000	conc
1537	o160	7.117650E-07	1.936570E-07	5.181080E-07	1.000	conc
1538	o161	6.901710E-07	1.827347E-07	5.074363E-07	1.000	conc
1539	o162	6.686150E-07	1.724191E-07	4.961959E-07	1.000	conc
1540	o163	6.488390E-07	1.626772E-07	4.861618E-07	1.000	conc
1541	o164	6.339230E-07	1.534776E-07	4.804454E-07	1.000	conc
1542	o165	6.272630E-07	1.447907E-07	4.824723E-07	1.000	conc
1543	o166	6.322520E-07	1.365884E-07	4.956636E-07	1.000	conc
1544	o167	6.483740E-07	1.288442E-07	5.195298E-07	1.000	conc
1545	o168	6.640750E-07	1.215328E-07	5.425422E-07	1.000	conc
1546	o169	6.717990E-07	1.146307E-07	5.571683E-07	1.000	conc
1547	o170	6.722490E-07	1.081151E-07	5.641339E-07	1.000	conc
1548	o171	6.667270E-07	1.019649E-07	5.647621E-07	1.000	conc
1549	o172	6.565360E-07	9.615986E-08	5.603761E-07	1.000	conc
1550	o173	6.429800E-07	9.068094E-08	5.522991E-07	1.000	conc
1551	o174	6.273620E-07	8.551011E-08	5.418519E-07	1.000	conc
1552	o175	6.109860E-07	8.063031E-08	5.303557E-07	1.000	conc
1553	o176	5.948490E-07	7.602543E-08	5.188236E-07	1.000	conc
1554	o177	5.787370E-07	7.168022E-08	5.070568E-07	1.000	conc
1555	o178	5.621310E-07	6.758025E-08	4.945508E-07	1.000	conc
1556	o179	5.445970E-07	6.371188E-08	4.808851E-07	1.000	conc
1557	o180	5.274260E-07	6.006224E-08	4.673638E-07	1.000	conc
1558	o181	5.135020E-07	5.661913E-08	4.568829E-07	1.000	conc
1559	o182	5.057720E-07	5.337104E-08	4.524010E-07	1.000	conc
1560	o183	5.056420E-07	5.030707E-08	4.553349E-07	1.000	conc
1561	o184	5.093550E-07	4.741694E-08	4.619381E-07	1.000	conc
1562	o185	5.120720E-07	4.469093E-08	4.673811E-07	1.000	conc
1563	o186	5.089570E-07	4.211983E-08	4.668372E-07	1.000	conc
1564	o187	4.979250E-07	3.969497E-08	4.582300E-07	1.000	conc
1565	o188	4.838970E-07	3.740814E-08	4.464889E-07	1.000	

						conc
1566	o189	4.728920E-07	3.525159E-08	4.376404E-07	1.000	conc
1567	o190	4.702190E-07	3.321799E-08	4.370010E-07	1.000	conc
1568	o191	4.733380E-07	3.130043E-08	4.420376E-07	1.000	conc
1569	o192	4.748510E-07	2.949237E-08	4.453586E-07	1.000	conc
1570	o193	4.673070E-07	2.778763E-08	4.395194E-07	1.000	conc
1571	o194	4.478510E-07	2.618039E-08	4.216706E-07	1.000	conc
1572	o195	4.235710E-07	2.466514E-08	3.989059E-07	1.000	conc
1573	o196	4.028560E-07	2.323668E-08	3.796193E-07	1.000	conc
1574	o197	3.940200E-07	2.189010E-08	3.721299E-07	1.000	conc
1575	o198	3.997040E-07	2.062076E-08	3.790832E-07	1.000	conc
1576	o199	4.130600E-07	1.942428E-08	3.936357E-07	1.000	conc
1577	o200	4.263430E-07	1.829653E-08	4.080465E-07	1.000	conc
1578	o201	4.326460E-07	1.723361E-08	4.154124E-07	1.000	conc
1579	o202	4.306550E-07	1.623184E-08	4.144232E-07	1.000	conc
1580	o203	4.213290E-07	1.528773E-08	4.060413E-07	1.000	conc
1581	o204	4.056960E-07	1.439801E-08	3.912980E-07	1.000	conc
1582	o205	3.878020E-07	1.355957E-08	3.742424E-07	1.000	conc
1583	o206	3.760210E-07	1.276950E-08	3.632515E-07	1.000	conc
1584	o207	3.790600E-07	1.202504E-08	3.670350E-07	1.000	conc
1585	o208	4.048110E-07	1.132357E-08	3.934874E-07	1.000	conc
1586	o209	4.380700E-07	1.066265E-08	4.274074E-07	1.000	conc
1587	o210	4.505410E-07	1.003995E-08	4.405010E-07	1.000	conc
1588	o211	4.448250E-07	9.453293E-09	4.353717E-07	1.000	conc
1589	o212	4.285460E-07	8.900607E-09	4.196454E-07	1.000	conc
1590	o213	4.093090E-07	8.379949E-09	4.009291E-07	1.000	conc
1591	o214	3.923830E-07	7.889479E-09	3.844935E-07	1.000	conc
1592	o215	3.785030E-07	7.427467E-09	3.710755E-07	1.000	conc
1593	o216	3.678840E-07	6.992277E-09	3.608917E-07	1.000	conc
1594	o217	3.606130E-07	6.582368E-09	3.540306E-07	1.000	conc
1595	o218	3.557500E-07	6.196285E-09	3.495537E-07	1.000	conc
1596	o219	3.518660E-07	5.832658E-09	3.460333E-07	1.000	conc
1597	o220	3.475430E-07	5.490191E-09	3.420528E-07	1.000	conc
1598	o221	3.421740E-07	5.167667E-09	3.370063E-07	1.000	conc
1599	o222	3.365050E-07	4.863935E-09	3.316411E-07	1.000	conc
1600	o223	3.314100E-07	4.577909E-09	3.268321E-07	1.000	conc
1601	o224	3.277660E-07	4.308568E-09	3.234574E-07	1.000	conc

1602	o225	3.266420E-07	4.054947E-09	3.225871E-07	1.000	conc
1603	o226	3.293540E-07	3.816137E-09	3.255379E-07	1.000	conc
1604	o227	3.372350E-07	3.591281E-09	3.336437E-07	1.000	conc
1605	o228	3.503730E-07	3.379570E-09	3.469934E-07	1.000	conc
1606	o229	3.627350E-07	3.180244E-09	3.595548E-07	1.000	conc
1607	o230	3.664120E-07	2.992584E-09	3.634194E-07	1.000	conc
1608	o231	3.538240E-07	2.815913E-09	3.510081E-07	1.000	conc
1609	o232	3.267250E-07	2.649593E-09	3.240754E-07	1.000	conc
1610	o233	2.972220E-07	2.493023E-09	2.947290E-07	1.000	conc
1611	o234	2.779620E-07	2.345637E-09	2.756164E-07	1.000	conc
1612	o235	2.807770E-07	2.206900E-09	2.785701E-07	1.000	conc
1613	o236	3.027670E-07	2.076308E-09	3.006907E-07	1.000	conc
1614	o237	3.282720E-07	1.953388E-09	3.263186E-07	1.000	conc
1615	o238	3.412080E-07	1.837693E-09	3.393703E-07	1.000	conc
1616	o239	3.313560E-07	1.728802E-09	3.296272E-07	1.000	conc
1617	o240	3.067770E-07	1.626317E-09	3.051507E-07	1.000	conc
1618	o241	2.790880E-07	1.529864E-09	2.775581E-07	1.000	conc
1619	o242	2.593120E-07	1.439092E-09	2.578729E-07	1.000	conc
1620	o243	2.500270E-07	1.353669E-09	2.486733E-07	1.000	conc
1621	o244	2.475120E-07	1.273281E-09	2.462387E-07	1.000	conc
1622	o245	2.478980E-07	1.197635E-09	2.467004E-07	1.000	conc
1623	o246	2.476030E-07	1.126452E-09	2.464765E-07	1.000	conc
1624	o247	2.459700E-07	1.059472E-09	2.449105E-07	1.000	conc
1625	o248	2.440310E-07	9.964474E-10	2.430346E-07	1.000	conc
1626	o249	2.428400E-07	9.371475E-10	2.419029E-07	1.000	conc
1627	o250	2.429470E-07	8.813534E-10	2.420656E-07	1.000	conc
1628	o251	2.438980E-07	8.288594E-10	2.430691E-07	1.000	conc
1629	o252	2.451130E-07	7.794717E-10	2.443335E-07	1.000	conc
1630	o253	2.460160E-07	7.330079E-10	2.452830E-07	1.000	conc
1631	o254	2.460300E-07	6.892960E-10	2.453407E-07	1.000	conc
1632	o255	2.445760E-07	6.481743E-10	2.439278E-07	1.000	conc
1633	o256	2.410770E-07	6.094904E-10	2.404675E-07	1.000	conc
1634	o257	2.349560E-07	5.731007E-10	2.343829E-07	1.000	conc
1635	o258	2.256350E-07	5.388702E-10	2.250961E-07	1.000	conc
1636	o259	2.127040E-07	5.066717E-10	2.121973E-07	1.000	conc
1637	o260	1.975550E-07	4.763852E-10	1.970786E-07	1.000	conc
1638	o261	1.826670E-07	4.478982E-10	1.822191E-07	1.000	

	conc				
1639	o262 conc	1.705390E-07	4.211043E-10	1.701179E-07	1.000
1640	o263 conc	1.636690E-07	3.959037E-10	1.632731E-07	1.000
1641	o264 conc	1.644490E-07	3.722022E-10	1.640768E-07	1.000
1642	o265 conc	1.726930E-07	3.499113E-10	1.723431E-07	1.000
1643	o266 conc	1.855120E-07	3.289475E-10	1.851831E-07	1.000
1644	o267 conc	1.998920E-07	3.092323E-10	1.995828E-07	1.000
1645	o268 conc	2.128170E-07	2.906919E-10	2.125263E-07	1.000
1646	o269 conc	2.212710E-07	2.732568E-10	2.209977E-07	1.000
1647	o270 conc	2.227080E-07	2.568613E-10	2.224511E-07	1.000
1648	o271 conc	2.176220E-07	2.414440E-10	2.173806E-07	1.000
1649	o272 conc	2.077180E-07	2.269469E-10	2.074911E-07	1.000
1650	o273 conc	1.947020E-07	2.133154E-10	1.944887E-07	1.000
1651	o274 conc	1.802810E-07	2.004980E-10	1.800805E-07	1.000
1652	o275 conc	1.661270E-07	1.884466E-10	1.659386E-07	1.000
1653	o276 conc	1.534240E-07	1.771155E-10	1.532469E-07	1.000
1654	o277 conc	1.430190E-07	1.664621E-10	1.428525E-07	1.000
1655	o278 conc	1.357500E-07	1.564460E-10	1.355936E-07	1.000
1656	o279 conc	1.324550E-07	1.470293E-10	1.323080E-07	1.000
1657	o280 conc	1.339740E-07	1.381763E-10	1.338358E-07	1.000
1658	o281 conc	1.405780E-07	1.298536E-10	1.404481E-07	1.000
1659	o282 conc	1.502300E-07	1.220296E-10	1.501080E-07	1.000
1660	o283 conc	1.603050E-07	1.146744E-10	1.601903E-07	1.000
1661	o284 conc	1.681780E-07	1.077603E-10	1.680702E-07	1.000
1662	o285 conc	1.712250E-07	1.012609E-10	1.711237E-07	1.000
1663	o286 conc	1.672560E-07	9.515150E-11	1.671608E-07	1.000
1664	o287 conc	1.574450E-07	8.940879E-11	1.573556E-07	1.000
1665	o288 conc	1.445270E-07	8.401091E-11	1.444430E-07	1.000
1666	o289 conc	1.312480E-07	7.893727E-11	1.311691E-07	1.000
1667	o290 conc	1.203520E-07	7.416849E-11	1.202778E-07	1.000
1668	o291 conc	1.145800E-07	6.968637E-11	1.145103E-07	1.000
1669	o292 conc	1.155330E-07	6.547376E-11	1.154675E-07	1.000
1670	o293 conc	1.218370E-07	6.151455E-11	1.217755E-07	1.000
1671	o294 conc	1.316430E-07	5.779358E-11	1.315852E-07	1.000
1672	o295 conc	1.431010E-07	5.429659E-11	1.430467E-07	1.000
1673	o296 conc	1.543620E-07	5.101017E-11	1.543110E-07	1.000
1674	o297 conc	1.637930E-07	4.792171E-11	1.637451E-07	1.000

1675	o298	conc	1.708000E-07	4.501935E-11	1.707550E-07	1.000
1676	o299	conc	1.751040E-07	4.229193E-11	1.750617E-07	1.000
1677	o300	conc	1.764210E-07	3.972897E-11	1.763813E-07	1.000
1678	o301	conc	1.744710E-07	3.732059E-11	1.744337E-07	1.000
1679	o302	conc	1.689780E-07	3.505752E-11	1.689429E-07	1.000
1680	o303	conc	1.601270E-07	3.293105E-11	1.600941E-07	1.000
1681	o304	conc	1.488840E-07	3.093296E-11	1.488531E-07	1.000
1682	o305	conc	1.362920E-07	2.905555E-11	1.362629E-07	1.000
1683	o306	conc	1.233950E-07	2.729157E-11	1.233677E-07	1.000
1684	o307	conc	1.112360E-07	2.563419E-11	1.112104E-07	1.000
1685	o308	conc	1.008570E-07	2.407701E-11	1.008329E-07	1.000
1686	o309	conc	9.328990E-08	2.261399E-11	9.326729E-08	1.000
1687	o310	conc	8.893630E-08	2.123947E-11	8.891506E-08	1.000
1688	o311	conc	8.728010E-08	1.994813E-11	8.726015E-08	1.000
1689	o312	conc	8.773220E-08	1.873496E-11	8.771347E-08	1.000
1690	o313	conc	8.970370E-08	1.759524E-11	8.968610E-08	1.000
1691	o314	conc	9.260560E-08	1.652455E-11	9.258908E-08	1.000
1692	o315	conc	9.584880E-08	1.551873E-11	9.583328E-08	1.000
1693	o316	conc	9.884430E-08	1.457387E-11	9.882973E-08	1.000
1694	o317	conc	1.011340E-07	1.368629E-11	1.011203E-07	1.000
1695	o318	conc	1.028180E-07	1.285254E-11	1.028051E-07	1.000
1696	o319	conc	1.041430E-07	1.206936E-11	1.041309E-07	1.000
1697	o320	conc	1.053590E-07	1.133370E-11	1.053477E-07	1.000
1698	o321	conc	1.067130E-07	1.064269E-11	1.067024E-07	1.000
1699	o322	conc	1.084520E-07	9.993643E-12	1.084420E-07	1.000
1700	o323	conc	1.108260E-07	9.384009E-12	1.108166E-07	1.000
1701	o324	conc	1.140510E-07	8.811410E-12	1.140422E-07	1.000
1702	o325	conc	1.178980E-07	8.273607E-12	1.178897E-07	1.000
1703	o326	conc	1.217920E-07	7.768494E-12	1.217842E-07	1.000
1704	o327	conc	1.251530E-07	7.294094E-12	1.251457E-07	1.000
1705	o328	conc	1.273980E-07	6.848547E-12	1.273912E-07	1.000
1706	o329	conc	1.279450E-07	6.430105E-12	1.279386E-07	1.000
1707	o330	conc	1.262120E-07	6.037128E-12	1.262060E-07	1.000
1708	o331	conc	1.216930E-07	5.668072E-12	1.216873E-07	1.000
1709	o332	conc	1.147900E-07	5.321487E-12	1.147847E-07	1.000
1710	o333	conc	1.064960E-07	4.996011E-12	1.064910E-07	1.000
1711	o334		9.781810E-08	4.690364E-12	9.781341E-08	1.000

	conc				
1712	o335 conc	8.976220E-08	4.403343E-12	8.975780E-08	1.000
1713	o336 conc	8.333400E-08	4.133818E-12	8.332987E-08	1.000
1714	o337 conc	7.953930E-08	3.880727E-12	7.953542E-08	1.000
1715	o338 conc	7.937580E-08	3.643071E-12	7.937216E-08	1.000
1716	o339 conc	8.310890E-08	3.419914E-12	8.310548E-08	1.000
1717	o340 conc	8.970640E-08	3.210375E-12	8.970319E-08	1.000
1718	o341 conc	9.800290E-08	3.013625E-12	9.799989E-08	1.000
1719	o342 conc	1.068330E-07	2.828888E-12	1.068302E-07	1.000
1720	o343 conc	1.150320E-07	2.655433E-12	1.150293E-07	1.000
1721	o344 conc	1.214330E-07	2.492574E-12	1.214305E-07	1.000
1722	o345 conc	1.248720E-07	2.339666E-12	1.248697E-07	1.000
1723	o346 conc	1.244490E-07	2.196104E-12	1.244468E-07	1.000
1724	o347 conc	1.206740E-07	2.061319E-12	1.206719E-07	1.000
1725	o348 conc	1.145190E-07	1.934775E-12	1.145171E-07	1.000
1726	o349 conc	1.069590E-07	1.815972E-12	1.069572E-07	1.000
1727	o350 conc	9.896780E-08	1.704438E-12	9.896610E-08	1.000
1728	o351 conc	9.151880E-08	1.599729E-12	9.151720E-08	1.000
1729	o352 conc	8.558610E-08	1.501430E-12	8.558460E-08	1.000
1730	o353 conc	8.210600E-08	1.409149E-12	8.210459E-08	1.000
1731	o354 conc	8.126640E-08	1.322520E-12	8.126508E-08	1.000
1732	o355 conc	8.256360E-08	1.241198E-12	8.256236E-08	1.000
1733	o356 conc	8.546820E-08	1.164859E-12	8.546704E-08	1.000
1734	o357 conc	8.945030E-08	1.093199E-12	8.944921E-08	1.000
1735	o358 conc	9.398060E-08	1.025931E-12	9.397957E-08	1.000
1736	o359 conc	9.852930E-08	9.627889E-13	9.852834E-08	1.000
1737	o360 conc	1.025670E-07	9.035193E-13	1.025661E-07	1.000
1738	o361 conc	1.056860E-07	8.478858E-13	1.056852E-07	1.000
1739	o362 conc	1.077830E-07	7.956662E-13	1.077822E-07	1.000
1740	o363 conc	1.088070E-07	7.466519E-13	1.088063E-07	1.000
1741	o364 conc	1.087020E-07	7.006466E-13	1.087013E-07	1.000
1742	o365 conc	1.074160E-07	6.574665E-13	1.074153E-07	1.000
1743	o366 conc	1.048950E-07	6.169387E-13	1.048944E-07	1.000
1744	o367 conc	1.010860E-07	5.789007E-13	1.010854E-07	1.000
1745	o368 conc	9.611800E-08	5.432009E-13	9.611746E-08	1.000
1746	o369 conc	9.050930E-08	5.096941E-13	9.050879E-08	1.000
1747	o370 conc	8.482960E-08	4.782480E-13	8.482912E-08	1.000

1748	o371 conc	7.964840E-08	4.487356E-13	7.964795E-08	1.000
1749	o372 conc	7.553540E-08	4.210385E-13	7.553498E-08	1.000
1750	o373 conc	7.306010E-08	3.950454E-13	7.305970E-08	1.000
1751	o374 conc	7.279220E-08	3.706518E-13	7.279183E-08	1.000
1752	o375 conc	7.514010E-08	3.477597E-13	7.513975E-08	1.000
1753	o376 conc	7.944540E-08	3.262769E-13	7.944507E-08	1.000
1754	o377 conc	8.461630E-08	3.061170E-13	8.461599E-08	1.000
1755	o378 conc	8.955980E-08	2.871987E-13	8.955951E-08	1.000
1756	o379 conc	9.318290E-08	2.694460E-13	9.318263E-08	1.000
1757	o380 conc	9.439240E-08	2.527871E-13	9.439215E-08	1.000
1758	o381 conc	9.209550E-08	2.371550E-13	9.209526E-08	1.000
1759	o382 conc	8.524380E-08	2.224866E-13	8.524358E-08	1.000
1760	o383 conc	7.405490E-08	2.087226E-13	7.405469E-08	1.000
1761	o384 conc	6.015780E-08	1.958075E-13	6.015760E-08	1.000
1762	o385 conc	4.525560E-08	1.836891E-13	4.525542E-08	1.000
1763	o386 conc	3.105130E-08	1.723184E-13	3.105113E-08	1.000
1764	o387 conc	1.924800E-08	1.616495E-13	1.924784E-08	1.000
1765	o388 conc	1.154880E-08	1.516391E-13	1.154865E-08	1.000
1766	o389 conc	9.269960E-09	1.422467E-13	9.269818E-09	1.000
1767	o390 conc	1.192830E-08	1.334343E-13	1.192817E-08	1.000
1768	o391 conc	1.851730E-08	1.251663E-13	1.851717E-08	1.000
1769	o392 conc	2.803040E-08	1.174090E-13	2.803028E-08	1.000
1770	o393 conc	3.946100E-08	1.101311E-13	3.946089E-08	1.000
1771	o394 conc	5.180260E-08	1.033030E-13	5.180250E-08	1.000
1772	o395 conc	6.404870E-08	9.689698E-14	6.404860E-08	1.000
1773	o396 conc	7.522440E-08	9.088706E-14	7.522431E-08	1.000
1774	o397 conc	8.488610E-08	8.524880E-14	8.488601E-08	1.000
1775	o398 conc	9.302900E-08	7.995931E-14	9.302892E-08	1.000
1776	o399 conc	9.966150E-08	7.499708E-14	9.966143E-08	1.000
1777	o400 conc	1.047920E-07	7.034191E-14	1.047919E-07	1.000
1778	o401 conc	1.084280E-07	6.597486E-14	1.084279E-07	1.000
1779	o402 conc	1.105790E-07	6.187817E-14	1.105789E-07	1.000
1780	o403 conc	1.112630E-07	5.803513E-14	1.112629E-07	1.000
1781	o404 conc	1.106210E-07	5.443010E-14	1.106209E-07	1.000
1782	o405 conc	1.088860E-07	5.104838E-14	1.088859E-07	1.000
1783	o406 conc	1.062910E-07	4.787617E-14	1.062910E-07	1.000
1784	o407	1.030690E-07	4.490054E-14	1.030690E-07	1.000

	conc				
1785	o408 conc	9.945440E-08	4.210933E-14	9.945436E-08	1.000
1786	o409 conc	9.568040E-08	3.949116E-14	9.568036E-08	1.000
1787	o410 conc	9.197960E-08	3.703533E-14	9.197956E-08	1.000
1788	o411 conc	8.849010E-08	3.473180E-14	8.849007E-08	1.000
1789	o412 conc	8.516850E-08	3.257115E-14	8.516847E-08	1.000
1790	o413 conc	8.195060E-08	3.054455E-14	8.195057E-08	1.000
1791	o414 conc	7.877250E-08	2.864371E-14	7.877247E-08	1.000
1792	o415 conc	7.557000E-08	2.686083E-14	7.556997E-08	1.000
1793	o416 conc	7.227910E-08	2.518863E-14	7.227907E-08	1.000
1794	o417 conc	6.883580E-08	2.362026E-14	6.883578E-08	1.000
1795	o418 conc	6.520510E-08	2.214928E-14	6.520508E-08	1.000
1796	o419 conc	6.152120E-08	2.076966E-14	6.152118E-08	1.000
1797	o420 conc	5.797910E-08	1.947575E-14	5.797908E-08	1.000
1798	o421 conc	5.477390E-08	1.826223E-14	5.477388E-08	1.000
1799	o422 conc	5.210060E-08	1.712413E-14	5.210058E-08	1.000
1800	o423 conc	5.015430E-08	1.605677E-14	5.015428E-08	1.000
1801	o424 conc	4.913010E-08	1.505576E-14	4.913008E-08	1.000
1802	o425 conc	4.914850E-08	1.411700E-14	4.914849E-08	1.000
1803	o426 conc	4.996780E-08	1.323662E-14	4.996779E-08	1.000
1804	o427 conc	5.123580E-08	1.241101E-14	5.123579E-08	1.000
1805	o428 conc	5.260070E-08	1.163675E-14	5.260069E-08	1.000
1806	o429 conc	5.372650E-08	1.091068E-14	5.372649E-08	1.000
1807	o430 conc	5.452150E-08	1.022979E-14	5.452149E-08	1.000
1808	o431 conc	5.508430E-08	9.591290E-15	5.508429E-08	1.000
1809	o432 conc	5.551840E-08	8.992539E-15	5.551839E-08	1.000
1810	o433 conc	5.592730E-08	8.431072E-15	5.592729E-08	1.000
1811	o434 conc	5.641470E-08	7.904574E-15	5.641469E-08	1.000
1812	o435 conc	5.708410E-08	7.410873E-15	5.708409E-08	1.000
1813	o436 conc	5.803860E-08	6.947930E-15	5.803859E-08	1.000
1814	o437 conc	5.931950E-08	6.513835E-15	5.931949E-08	1.000
1815	o438 conc	6.083730E-08	6.106794E-15	6.083729E-08	1.000
1816	o439 conc	6.248570E-08	5.725127E-15	6.248569E-08	1.000
1817	o440 conc	6.415840E-08	5.367255E-15	6.415839E-08	1.000
1818	o441 conc	6.574900E-08	5.031698E-15	6.574899E-08	1.000
1819	o442 conc	6.715150E-08	4.717070E-15	6.715150E-08	1.000
1820	o443 conc	6.825940E-08	4.422067E-15	6.825940E-08	1.000

1821	o444 conc	6.897850E-08	4.145469E-15	6.897850E-08	1.000
1822	o445 conc	6.929210E-08	3.886131E-15	6.929210E-08	1.000
1823	o446 conc	6.921410E-08	3.642978E-15	6.921410E-08	1.000
1824	o447 conc	6.875870E-08	3.415003E-15	6.875870E-08	1.000
1825	o448 conc	6.793980E-08	3.201260E-15	6.793980E-08	1.000
1826	o449 conc	6.677160E-08	3.000864E-15	6.677160E-08	1.000
1827	o450 conc	6.526820E-08	2.812983E-15	6.526820E-08	1.000
1828	o451 conc	6.346600E-08	2.636837E-15	6.346600E-08	1.000
1829	o452 conc	6.152200E-08	2.471696E-15	6.152200E-08	1.000
1830	o453 conc	5.963400E-08	2.316873E-15	5.963400E-08	1.000
1831	o454 conc	5.799960E-08	2.171725E-15	5.799960E-08	1.000
1832	o455 conc	5.681660E-08	2.035650E-15	5.681660E-08	1.000
1833	o456 conc	5.628270E-08	1.908081E-15	5.628270E-08	1.000
1834	o457 conc	5.659560E-08	1.788488E-15	5.659560E-08	1.000
1835	o458 conc	5.793820E-08	1.676374E-15	5.793820E-08	1.000
1836	o459 conc	6.018830E-08	1.571272E-15	6.018830E-08	1.000
1837	o460 conc	6.293640E-08	1.472744E-15	6.293640E-08	1.000
1838	o461 conc	6.576190E-08	1.380381E-15	6.576190E-08	1.000
1839	o462 conc	6.824420E-08	1.293797E-15	6.824420E-08	1.000
1840	o463 conc	6.996260E-08	1.212632E-15	6.996260E-08	1.000
1841	o464 conc	7.049670E-08	1.136547E-15	7.049670E-08	1.000
1842	o465 conc	6.942630E-08	1.065226E-15	6.942630E-08	1.000
1843	o466 conc	6.658270E-08	9.983701E-16	6.658270E-08	1.000
1844	o467 conc	6.244930E-08	9.357010E-16	6.244930E-08	1.000
1845	o468 conc	5.761370E-08	8.769571E-16	5.761370E-08	1.000
1846	o469 conc	5.266350E-08	8.218930E-16	5.266350E-08	1.000
1847	o470 conc	4.818620E-08	7.702788E-16	4.818620E-08	1.000
1848	o471 conc	4.476960E-08	7.218989E-16	4.476960E-08	1.000
1849	o472 conc	4.300000E-08	6.765511E-16	4.300000E-08	1.000
1850	o473 conc	4.321500E-08	6.340457E-16	4.321500E-08	1.000
1851	o474 conc	4.520450E-08	5.942050E-16	4.520450E-08	1.000
1852	o475 conc	4.868480E-08	5.568624E-16	4.868480E-08	1.000
1853	o476 conc	5.337270E-08	5.218615E-16	5.337270E-08	1.000
1854	o477 conc	5.898460E-08	4.890558E-16	5.898460E-08	1.000
1855	o478 conc	6.523700E-08	4.583080E-16	6.523700E-08	1.000
1856	o479 conc	7.184670E-08	4.294893E-16	7.184670E-08	1.000
1857	o480	7.851040E-08	4.024789E-16	7.851040E-08	1.000

```
conc
o481                  8.479280E-08   3.771635E-16   8.479280E-08    1.000
conc
o482                  9.020340E-08   3.534372E-16   9.020340E-08    1.000
conc
o483                  9.425170E-08   3.312003E-16   9.425170E-08    1.000
conc
o484                  9.644720E-08   3.103595E-16   9.644720E-08    1.000
conc
o485                  9.629920E-08   2.908274E-16   9.629920E-08    1.000
conc
o486                  9.331730E-08   2.725220E-16   9.331730E-08    1.000
conc
o487                  8.703580E-08   2.553664E-16   8.703580E-08    1.000
conc
o488                  7.771540E-08   2.392885E-16   7.771540E-08    1.000
conc
o489                  6.644230E-08   2.242208E-16   6.644230E-08    1.000
conc
o490                  5.434720E-08   2.101000E-16   5.434720E-08    1.000
conc
o491                  4.256060E-08   1.968667E-16   4.256060E-08    1.000
conc
o492                  3.221320E-08   1.844652E-16   3.221320E-08    1.000
conc
o493                  2.443570E-08   1.728433E-16   2.443570E-08    1.000
conc
o494                  2.032130E-08   1.619522E-16   2.032130E-08    1.000
conc
o495                  2.013560E-08   1.517459E-16   2.013560E-08    1.000
conc
o496                  2.333550E-08   1.421816E-16   2.333550E-08    1.000
conc
o497                  2.934400E-08   1.332189E-16   2.934400E-08    1.000
conc
o498                  3.758420E-08   1.248201E-16   3.758420E-08    1.000
conc
o499                  4.747890E-08   1.169497E-16   4.747890E-08    1.000
conc
o500                  5.845140E-08   1.095746E-16   5.845140E-08    1.000
conc

See file mfit.res for more details of residuals in graph-ready format.

See file mfit.seo for composite observation sensitivities.


Objective function ----->

  Sum of squared weighted residuals (ie phi)                =  9.3045E-11


Correlation Coefficient ----->

  Correlation coefficient                                   =  0.99790


Analysis of residuals ----->

  All residuals:-
     Number of residuals with non-zero weight                       =   500
     Mean value of non-zero weighted residuals                      =  1.3930E-07
     Maximum weighted residual [observation "o80"]                  =  3.1737E-06
     Minimum weighted residual [observation "o72"]                  = -2.3124E-06
     Standard variance of weighted residuals                        =  1.8759E-13
     Standard error of weighted residuals                           =  4.3312E-07

     Note: the above variance was obtained by dividing the objective
     function by the number of system degrees of freedom (ie. number of
     observations with non-zero weight plus number of prior information
     articles with non-zero weight minus the number of adjustable parameters.)
     If the degrees of freedom is negative the divisor becomes
     the number of observations with non-zero weight plus the number of
```

```
      prior information items with non-zero weight.


Covariance and other statistical matrices cannot be determined:-
Memory conservation is operative so that covariance matrix is not calculated.
```
