# Supplementary material for: Comparing the Fate and Transport of MS2 Bacteriophage and Sodium Fluorescein in a Karstic Chalk Aquifer
Source: Pathogens. 2024 Feb 13;13(2):168. doi: 10.3390/pathogens13020168 (PMC10891704; doi:10.3390/pathogens13020168)
Supplement: Supplementary file 1 [file pathogens-13-00168-s001.zip › ABH1_phage_mfit.pdf]

```
                         PEST RUN RECORD: CASE mfit


PEST Version: 16.1


PEST run mode:-

   Parameter estimation mode


Case dimensions:-

   Number of parameters                           :     4
   Number of adjustable parameters                :     4
   Number of parameter groups                     :     1
   Number of observations                         :   500
   Number of prior estimates                      :     0


Model command line(s):-

   MDP_2RNE


Jacobian command line:-

   MDP_2RNE /d
   Jacobian read from file Deriv.txt
   This is an ASCII file.


Model interface files:-

   Templates:
      MFIT.tpl
   for model input files:
      Input.txt

   (Parameter values written using single precision protocol.)
   (Decimal point always included.)

   Instruction files:
      MFIT.ins
   for reading model output files:
      Output.txt


PEST-to-model message file:-

   na


Singular value decomposition:-

   Perform SVD on XtQX or Q^(1/2)X               : Q^(1/2)X
   Max. number of singular values to employ      : 4
   Ratio of lowest/highest singular value        :  5.000000E-07
   Record eigenvectors in SVD file               : yes


Derivatives calculation:-

Param        Increment   Increment   Increment   Forward or   Multiplier  Method
group        type                    low bound   central      (central)   (central)
pgnam        relative    1.0000E-02   none         switch       1.500     parabolic


Parameter definitions:-

Name         Trans-             Change       Initial        Lower          Upper
             formation          limit        value          bound          bound
```

```
h1          log             factor      33.0000        30.0000        50.0000
s1          log             factor      510.000        1.00000        5000.00
p1          log             factor     0.800000       0.100000       0.999000
o1          log             factor     7.500000E-04   1.000000E-10   1.000000E-02

Name        Group          Scale         Offset        Model command number
h1          pgnam         1.00000        0.00000            1
s1          pgnam         1.00000        0.00000            0
p1          pgnam         1.00000        0.00000            1
o1          pgnam         1.00000        0.00000            0


Prior information:-

   No prior information supplied


Observations:-

Observation name        Observation        Weight       Group
 o1                     5.186150E-16       1.000       conc
 o2                     6.614330E-16       1.000       conc
 o3                     6.755630E-16       1.000       conc
 o4                     2.107200E-14       1.000       conc
 o5                      0.00000           1.000       conc
 o6                     3.543900E-13       1.000       conc
 o7                      0.00000           1.000       conc
 o8                     4.614550E-12       1.000       conc
 o9                      0.00000           1.000       conc
 o10                    4.829760E-11       1.000       conc
 o11                     0.00000           1.000       conc
 o12                    3.682830E-10       1.000       conc
 o13                    2.847080E-10       1.000       conc
 o14                    1.012680E-09       1.000       conc
 o15                    1.234790E-08       1.000       conc
 o16                     0.00000           1.000       conc
 o17                    2.207160E-07       1.000       conc
 o18                     0.00000           1.000       conc
 o19                    2.982970E-06       1.000       conc
 o20                     0.00000           1.000       conc
 o21                    3.844780E-05       1.000       conc
 o22                    1.332810E-04       1.000       conc
 o23                    4.524700E-05       1.000       conc
 o24                     0.00000           1.000       conc
 o25                     0.00000           1.000       conc
 o26                    3.313920E-03       1.000       conc
 o27                    4.709680E-03       1.000       conc
 o28                    3.738630E-03       1.000       conc
 o29                     0.00000           1.000       conc
 o30                    0.795515           1.000       conc
 o31                     0.00000           1.000       conc
 o32                     18.0567           1.000       conc
 o33                     0.00000           1.000       conc
 o34                     158.617           1.000       conc
 o35                     1021.51           1.000       conc
 o36                     25461.2           1.000       conc
 o37                     54889.1           1.000       conc
 o38                     123252.           1.000       conc
 o39                     439629.           1.000       conc
 o40                     997271.           1.000       conc
 o41                    1.774430E+06       1.000       conc
 o42                    2.486710E+06       1.000       conc
 o43                    4.526740E+06       1.000       conc
 o44                    1.324490E+07       1.000       conc
 o45                    1.652660E+07       1.000       conc
 o46                    1.706350E+07       1.000       conc
 o47                    1.896430E+07       1.000       conc
 o48                    2.344730E+07       1.000       conc
 o49                    1.701390E+07       1.000       conc
 o50                    7.951230E+06       1.000       conc
 o51                    9.231250E+06       1.000       conc
 o52                    8.369040E+06       1.000       conc
 o53                    7.774150E+06       1.000       conc
```

| | | | |
|---|---|---|---|
| o54 | 1.047680E+07 | 1.000 | conc |
| o55 | 1.058310E+07 | 1.000 | conc |
| o56 | 7.333510E+06 | 1.000 | conc |
| o57 | 6.669610E+06 | 1.000 | conc |
| o58 | 5.939640E+06 | 1.000 | conc |
| o59 | 9.578960E+06 | 1.000 | conc |
| o60 | 1.000890E+07 | 1.000 | conc |
| o61 | 9.376560E+06 | 1.000 | conc |
| o62 | 1.006980E+07 | 1.000 | conc |
| o63 | 4.971070E+06 | 1.000 | conc |
| o64 | 2.447770E+06 | 1.000 | conc |
| o65 | 3.784400E+06 | 1.000 | conc |
| o66 | 9.389320E+06 | 1.000 | conc |
| o67 | 1.016350E+07 | 1.000 | conc |
| o68 | 5.345640E+06 | 1.000 | conc |
| o69 | 5.859810E+06 | 1.000 | conc |
| o70 | 5.246670E+06 | 1.000 | conc |
| o71 | 6.640150E+06 | 1.000 | conc |
| o72 | 5.332380E+06 | 1.000 | conc |
| o73 | 3.356340E+06 | 1.000 | conc |
| o74 | 2.774270E+06 | 1.000 | conc |
| o75 | 2.385890E+06 | 1.000 | conc |
| o76 | 2.952400E+06 | 1.000 | conc |
| o77 | 2.057460E+06 | 1.000 | conc |
| o78 | 1.584830E+06 | 1.000 | conc |
| o79 | 2.916260E+06 | 1.000 | conc |
| o80 | 2.096230E+06 | 1.000 | conc |
| o81 | 2.504470E+06 | 1.000 | conc |
| o82 | 1.829420E+06 | 1.000 | conc |
| o83 | 830066. | 1.000 | conc |
| o84 | 2.518770E+06 | 1.000 | conc |
| o85 | 2.285180E+06 | 1.000 | conc |
| o86 | 1.772680E+06 | 1.000 | conc |
| o87 | 1.961070E+06 | 1.000 | conc |
| o88 | 1.718450E+06 | 1.000 | conc |
| o89 | 1.663840E+06 | 1.000 | conc |
| o90 | 1.764140E+06 | 1.000 | conc |
| o91 | 1.492310E+06 | 1.000 | conc |
| o92 | 1.283080E+06 | 1.000 | conc |
| o93 | 1.473610E+06 | 1.000 | conc |
| o94 | 1.738380E+06 | 1.000 | conc |
| o95 | 1.675570E+06 | 1.000 | conc |
| o96 | 1.604000E+06 | 1.000 | conc |
| o97 | 889930. | 1.000 | conc |
| o98 | 0.00000 | 1.000 | conc |
| o99 | 0.00000 | 1.000 | conc |
| o100 | 955486. | 1.000 | conc |
| o101 | 1.738130E+06 | 1.000 | conc |
| o102 | 310564. | 1.000 | conc |
| o103 | 0.00000 | 1.000 | conc |
| o104 | 202705. | 1.000 | conc |
| o105 | 166223. | 1.000 | conc |
| o106 | 110561. | 1.000 | conc |
| o107 | 86750.9 | 1.000 | conc |
| o108 | 80882.2 | 1.000 | conc |
| o109 | 89213.7 | 1.000 | conc |
| o110 | 113375. | 1.000 | conc |
| o111 | 136223. | 1.000 | conc |
| o112 | 138918. | 1.000 | conc |
| o113 | 121682. | 1.000 | conc |
| o114 | 121917. | 1.000 | conc |
| o115 | 140363. | 1.000 | conc |
| o116 | 138477. | 1.000 | conc |
| o117 | 118625. | 1.000 | conc |
| o118 | 103562. | 1.000 | conc |
| o119 | 101237. | 1.000 | conc |
| o120 | 107748. | 1.000 | conc |
| o121 | 102626. | 1.000 | conc |
| o122 | 75468.0 | 1.000 | conc |
| o123 | 61503.4 | 1.000 | conc |
| o124 | 76858.3 | 1.000 | conc |
| o125 | 83796.5 | 1.000 | conc |
| o126 | 75864.9 | 1.000 | conc |

| | | | |
|---|---|---|---|
| o127 | 78627.1 | 1.000 | conc |
| o128 | 82993.1 | 1.000 | conc |
| o129 | 70929.6 | 1.000 | conc |
| o130 | 61300.5 | 1.000 | conc |
| o131 | 66857.4 | 1.000 | conc |
| o132 | 70288.6 | 1.000 | conc |
| o133 | 58739.3 | 1.000 | conc |
| o134 | 46363.6 | 1.000 | conc |
| o135 | 48146.6 | 1.000 | conc |
| o136 | 69423.2 | 1.000 | conc |
| o137 | 90789.1 | 1.000 | conc |
| o138 | 78851.6 | 1.000 | conc |
| o139 | 55522.4 | 1.000 | conc |
| o140 | 56299.4 | 1.000 | conc |
| o141 | 72537.0 | 1.000 | conc |
| o142 | 86973.6 | 1.000 | conc |
| o143 | 84790.9 | 1.000 | conc |
| o144 | 62089.3 | 1.000 | conc |
| o145 | 47548.6 | 1.000 | conc |
| o146 | 52955.3 | 1.000 | conc |
| o147 | 55351.2 | 1.000 | conc |
| o148 | 51979.7 | 1.000 | conc |
| o149 | 55427.2 | 1.000 | conc |
| o150 | 53199.8 | 1.000 | conc |
| o151 | 33000.8 | 1.000 | conc |
| o152 | 18162.2 | 1.000 | conc |
| o153 | 22739.4 | 1.000 | conc |
| o154 | 33621.3 | 1.000 | conc |
| o155 | 39197.2 | 1.000 | conc |
| o156 | 39125.2 | 1.000 | conc |
| o157 | 35025.4 | 1.000 | conc |
| o158 | 28518.2 | 1.000 | conc |
| o159 | 21223.7 | 1.000 | conc |
| o160 | 14762.2 | 1.000 | conc |
| o161 | 10753.4 | 1.000 | conc |
| o162 | 10066.4 | 1.000 | conc |
| o163 | 11344.4 | 1.000 | conc |
| o164 | 13039.5 | 1.000 | conc |
| o165 | 14764.0 | 1.000 | conc |
| o166 | 16068.4 | 1.000 | conc |
| o167 | 15455.8 | 1.000 | conc |
| o168 | 14663.3 | 1.000 | conc |
| o169 | 19091.1 | 1.000 | conc |
| o170 | 24352.6 | 1.000 | conc |
| o171 | 21494.7 | 1.000 | conc |
| o172 | 14671.8 | 1.000 | conc |
| o173 | 10655.3 | 1.000 | conc |
| o174 | 10844.5 | 1.000 | conc |
| o175 | 14963.9 | 1.000 | conc |
| o176 | 19019.4 | 1.000 | conc |
| o177 | 19892.0 | 1.000 | conc |
| o178 | 19083.0 | 1.000 | conc |
| o179 | 18695.9 | 1.000 | conc |
| o180 | 20563.9 | 1.000 | conc |
| o181 | 24196.4 | 1.000 | conc |
| o182 | 27794.8 | 1.000 | conc |
| o183 | 30310.9 | 1.000 | conc |
| o184 | 31421.3 | 1.000 | conc |
| o185 | 31982.9 | 1.000 | conc |
| o186 | 31860.6 | 1.000 | conc |
| o187 | 25884.9 | 1.000 | conc |
| o188 | 13221.5 | 1.000 | conc |
| o189 | 4834.01 | 1.000 | conc |
| o190 | 5004.64 | 1.000 | conc |
| o191 | 9007.62 | 1.000 | conc |
| o192 | 11966.3 | 1.000 | conc |
| o193 | 12294.3 | 1.000 | conc |
| o194 | 22618.7 | 1.000 | conc |
| o195 | 46542.1 | 1.000 | conc |
| o196 | 59116.4 | 1.000 | conc |
| o197 | 54086.6 | 1.000 | conc |
| o198 | 49842.9 | 1.000 | conc |
| o199 | 49878.8 | 1.000 | conc |

| | | | |
|---|---|---|---|
| o200 | 48086.8 | 1.000 | conc |
| o201 | 45371.6 | 1.000 | conc |
| o202 | 48500.7 | 1.000 | conc |
| o203 | 52492.5 | 1.000 | conc |
| o204 | 49431.0 | 1.000 | conc |
| o205 | 45507.9 | 1.000 | conc |
| o206 | 45413.5 | 1.000 | conc |
| o207 | 43509.8 | 1.000 | conc |
| o208 | 39265.3 | 1.000 | conc |
| o209 | 38196.0 | 1.000 | conc |
| o210 | 39207.9 | 1.000 | conc |
| o211 | 37626.7 | 1.000 | conc |
| o212 | 34032.5 | 1.000 | conc |
| o213 | 33337.7 | 1.000 | conc |
| o214 | 36078.7 | 1.000 | conc |
| o215 | 39600.7 | 1.000 | conc |
| o216 | 37543.5 | 1.000 | conc |
| o217 | 30230.7 | 1.000 | conc |
| o218 | 31305.9 | 1.000 | conc |
| o219 | 39101.2 | 1.000 | conc |
| o220 | 37756.0 | 1.000 | conc |
| o221 | 29402.1 | 1.000 | conc |
| o222 | 25088.1 | 1.000 | conc |
| o223 | 26564.8 | 1.000 | conc |
| o224 | 28123.6 | 1.000 | conc |
| o225 | 27390.2 | 1.000 | conc |
| o226 | 24404.7 | 1.000 | conc |
| o227 | 22055.8 | 1.000 | conc |
| o228 | 21263.4 | 1.000 | conc |
| o229 | 18848.2 | 1.000 | conc |
| o230 | 16756.2 | 1.000 | conc |
| o231 | 20731.4 | 1.000 | conc |
| o232 | 27836.6 | 1.000 | conc |
| o233 | 31507.7 | 1.000 | conc |
| o234 | 32254.2 | 1.000 | conc |
| o235 | 34888.8 | 1.000 | conc |
| o236 | 36437.5 | 1.000 | conc |
| o237 | 33037.8 | 1.000 | conc |
| o238 | 27844.2 | 1.000 | conc |
| o239 | 23663.0 | 1.000 | conc |
| o240 | 20639.0 | 1.000 | conc |
| o241 | 18686.5 | 1.000 | conc |
| o242 | 17719.8 | 1.000 | conc |
| o243 | 17653.0 | 1.000 | conc |
| o244 | 18400.6 | 1.000 | conc |
| o245 | 19876.7 | 1.000 | conc |
| o246 | 21947.8 | 1.000 | conc |
| o247 | 22959.0 | 1.000 | conc |
| o248 | 19972.5 | 1.000 | conc |
| o249 | 15696.4 | 1.000 | conc |
| o250 | 14824.8 | 1.000 | conc |
| o251 | 14163.1 | 1.000 | conc |
| o252 | 10938.9 | 1.000 | conc |
| o253 | 9297.15 | 1.000 | conc |
| o254 | 10474.8 | 1.000 | conc |
| o255 | 10806.7 | 1.000 | conc |
| o256 | 8796.51 | 1.000 | conc |
| o257 | 8014.11 | 1.000 | conc |
| o258 | 9493.13 | 1.000 | conc |
| o259 | 11093.2 | 1.000 | conc |
| o260 | 13360.3 | 1.000 | conc |
| o261 | 17159.2 | 1.000 | conc |
| o262 | 17469.6 | 1.000 | conc |
| o263 | 12117.2 | 1.000 | conc |
| o264 | 8708.14 | 1.000 | conc |
| o265 | 10278.4 | 1.000 | conc |
| o266 | 13136.8 | 1.000 | conc |
| o267 | 14682.0 | 1.000 | conc |
| o268 | 15092.5 | 1.000 | conc |
| o269 | 14703.9 | 1.000 | conc |
| o270 | 13870.6 | 1.000 | conc |
| o271 | 14481.2 | 1.000 | conc |
| o272 | 17999.1 | 1.000 | conc |

| | | | |
|---|---|---|---|
| o273 | 20879.1 | 1.000 | conc |
| o274 | 20813.3 | 1.000 | conc |
| o275 | 20470.0 | 1.000 | conc |
| o276 | 20325.7 | 1.000 | conc |
| o277 | 17950.9 | 1.000 | conc |
| o278 | 13279.2 | 1.000 | conc |
| o279 | 10433.2 | 1.000 | conc |
| o280 | 10368.9 | 1.000 | conc |
| o281 | 10902.9 | 1.000 | conc |
| o282 | 10993.4 | 1.000 | conc |
| o283 | 10436.0 | 1.000 | conc |
| o284 | 9908.35 | 1.000 | conc |
| o285 | 9893.18 | 1.000 | conc |
| o286 | 10141.9 | 1.000 | conc |
| o287 | 10404.2 | 1.000 | conc |
| o288 | 10507.0 | 1.000 | conc |
| o289 | 10500.4 | 1.000 | conc |
| o290 | 10687.5 | 1.000 | conc |
| o291 | 11015.6 | 1.000 | conc |
| o292 | 11336.7 | 1.000 | conc |
| o293 | 11502.8 | 1.000 | conc |
| o294 | 11425.5 | 1.000 | conc |
| o295 | 11217.6 | 1.000 | conc |
| o296 | 11033.7 | 1.000 | conc |
| o297 | 11018.9 | 1.000 | conc |
| o298 | 11137.2 | 1.000 | conc |
| o299 | 11192.9 | 1.000 | conc |
| o300 | 10984.6 | 1.000 | conc |
| o301 | 10377.9 | 1.000 | conc |
| o302 | 9476.11 | 1.000 | conc |
| o303 | 8435.45 | 1.000 | conc |
| o304 | 7412.14 | 1.000 | conc |
| o305 | 6542.14 | 1.000 | conc |
| o306 | 5899.57 | 1.000 | conc |
| o307 | 5546.75 | 1.000 | conc |
| o308 | 5538.49 | 1.000 | conc |
| o309 | 5808.09 | 1.000 | conc |
| o310 | 6190.55 | 1.000 | conc |
| o311 | 6518.06 | 1.000 | conc |
| o312 | 6669.54 | 1.000 | conc |
| o313 | 6674.79 | 1.000 | conc |
| o314 | 6594.03 | 1.000 | conc |
| o315 | 6487.49 | 1.000 | conc |
| o316 | 6423.96 | 1.000 | conc |
| o317 | 6495.94 | 1.000 | conc |
| o318 | 6800.02 | 1.000 | conc |
| o319 | 7419.38 | 1.000 | conc |
| o320 | 8246.96 | 1.000 | conc |
| o321 | 9034.60 | 1.000 | conc |
| o322 | 9530.89 | 1.000 | conc |
| o323 | 9568.11 | 1.000 | conc |
| o324 | 9223.84 | 1.000 | conc |
| o325 | 8620.39 | 1.000 | conc |
| o326 | 7880.08 | 1.000 | conc |
| o327 | 7125.52 | 1.000 | conc |
| o328 | 6480.04 | 1.000 | conc |
| o329 | 6067.10 | 1.000 | conc |
| o330 | 5990.81 | 1.000 | conc |
| o331 | 6113.39 | 1.000 | conc |
| o332 | 6132.63 | 1.000 | conc |
| o333 | 5743.19 | 1.000 | conc |
| o334 | 4692.77 | 1.000 | conc |
| o335 | 3278.84 | 1.000 | conc |
| o336 | 2124.16 | 1.000 | conc |
| o337 | 1855.75 | 1.000 | conc |
| o338 | 2987.66 | 1.000 | conc |
| o339 | 5053.39 | 1.000 | conc |
| o340 | 7082.62 | 1.000 | conc |
| o341 | 8104.81 | 1.000 | conc |
| o342 | 7667.29 | 1.000 | conc |
| o343 | 6347.40 | 1.000 | conc |
| o344 | 4844.30 | 1.000 | conc |
| o345 | 3800.34 | 1.000 | conc |

| | | | |
|---|---|---|---|
| o346 | 3332.00 | 1.000 | conc |
| o347 | 3271.28 | 1.000 | conc |
| o348 | 3447.37 | 1.000 | conc |
| o349 | 3697.06 | 1.000 | conc |
| o350 | 3916.26 | 1.000 | conc |
| o351 | 4028.66 | 1.000 | conc |
| o352 | 3958.99 | 1.000 | conc |
| o353 | 3720.92 | 1.000 | conc |
| o354 | 3489.71 | 1.000 | conc |
| o355 | 3457.76 | 1.000 | conc |
| o356 | 3786.29 | 1.000 | conc |
| o357 | 4378.55 | 1.000 | conc |
| o358 | 5010.24 | 1.000 | conc |
| o359 | 5456.18 | 1.000 | conc |
| o360 | 5519.01 | 1.000 | conc |
| o361 | 5196.47 | 1.000 | conc |
| o362 | 4569.70 | 1.000 | conc |
| o363 | 3720.87 | 1.000 | conc |
| o364 | 2783.53 | 1.000 | conc |
| o365 | 1976.63 | 1.000 | conc |
| o366 | 1527.18 | 1.000 | conc |
| o367 | 1625.43 | 1.000 | conc |
| o368 | 2187.95 | 1.000 | conc |
| o369 | 3007.88 | 1.000 | conc |
| o370 | 3877.77 | 1.000 | conc |
| o371 | 4609.32 | 1.000 | conc |
| o372 | 5135.25 | 1.000 | conc |
| o373 | 5435.37 | 1.000 | conc |
| o374 | 5489.98 | 1.000 | conc |
| o375 | 5300.82 | 1.000 | conc |
| o376 | 4902.23 | 1.000 | conc |
| o377 | 4331.65 | 1.000 | conc |
| o378 | 3660.98 | 1.000 | conc |
| o379 | 3025.59 | 1.000 | conc |
| o380 | 2567.69 | 1.000 | conc |
| o381 | 2416.88 | 1.000 | conc |
| o382 | 2596.80 | 1.000 | conc |
| o383 | 3077.96 | 1.000 | conc |
| o384 | 3830.52 | 1.000 | conc |
| o385 | 4812.50 | 1.000 | conc |
| o386 | 5895.83 | 1.000 | conc |
| o387 | 6915.08 | 1.000 | conc |
| o388 | 7705.17 | 1.000 | conc |
| o389 | 8137.47 | 1.000 | conc |
| o390 | 8144.74 | 1.000 | conc |
| o391 | 7665.61 | 1.000 | conc |
| o392 | 6666.14 | 1.000 | conc |
| o393 | 5319.59 | 1.000 | conc |
| o394 | 3893.92 | 1.000 | conc |
| o395 | 2657.58 | 1.000 | conc |
| o396 | 1843.08 | 1.000 | conc |
| o397 | 1452.31 | 1.000 | conc |
| o398 | 1396.14 | 1.000 | conc |
| o399 | 1586.28 | 1.000 | conc |
| o400 | 1996.92 | 1.000 | conc |
| o401 | 2698.55 | 1.000 | conc |
| o402 | 3769.91 | 1.000 | conc |
| o403 | 5245.89 | 1.000 | conc |
| o404 | 6863.28 | 1.000 | conc |
| o405 | 8234.65 | 1.000 | conc |
| o406 | 8972.15 | 1.000 | conc |
| o407 | 8786.59 | 1.000 | conc |
| o408 | 7959.86 | 1.000 | conc |
| o409 | 6978.88 | 1.000 | conc |
| o410 | 6322.32 | 1.000 | conc |
| o411 | 6064.89 | 1.000 | conc |
| o412 | 5710.94 | 1.000 | conc |
| o413 | 4721.68 | 1.000 | conc |
| o414 | 2762.35 | 1.000 | conc |
| o415 | 750.199 | 1.000 | conc |
| o416 | 77.2322 | 1.000 | conc |
| o417 | 2136.35 | 1.000 | conc |
| o418 | 6022.50 | 1.000 | conc |

| | | | |
|---|---|---|---|
| o419 | 6694.25 | 1.000 | conc |
| o420 | 5144.10 | 1.000 | conc |
| o421 | 2885.38 | 1.000 | conc |
| o422 | 1382.02 | 1.000 | conc |
| o423 | 1080.33 | 1.000 | conc |
| o424 | 1470.63 | 1.000 | conc |
| o425 | 2006.22 | 1.000 | conc |
| o426 | 2257.46 | 1.000 | conc |
| o427 | 2243.07 | 1.000 | conc |
| o428 | 2089.08 | 1.000 | conc |
| o429 | 1918.42 | 1.000 | conc |
| o430 | 1783.93 | 1.000 | conc |
| o431 | 1669.31 | 1.000 | conc |
| o432 | 1555.32 | 1.000 | conc |
| o433 | 1425.76 | 1.000 | conc |
| o434 | 1318.85 | 1.000 | conc |
| o435 | 1319.69 | 1.000 | conc |
| o436 | 1514.93 | 1.000 | conc |
| o437 | 1927.13 | 1.000 | conc |
| o438 | 2356.48 | 1.000 | conc |
| o439 | 2554.99 | 1.000 | conc |
| o440 | 2290.19 | 1.000 | conc |
| o441 | 1630.71 | 1.000 | conc |
| o442 | 917.822 | 1.000 | conc |
| o443 | 502.633 | 1.000 | conc |
| o444 | 710.489 | 1.000 | conc |
| o445 | 1465.38 | 1.000 | conc |
| o446 | 2374.01 | 1.000 | conc |
| o447 | 3034.49 | 1.000 | conc |
| o448 | 3179.19 | 1.000 | conc |
| o449 | 2963.08 | 1.000 | conc |
| o450 | 2624.07 | 1.000 | conc |
| o451 | 2389.02 | 1.000 | conc |
| o452 | 2299.28 | 1.000 | conc |
| o453 | 2242.04 | 1.000 | conc |
| o454 | 2099.86 | 1.000 | conc |
| o455 | 1768.94 | 1.000 | conc |
| o456 | 1332.86 | 1.000 | conc |
| o457 | 1010.96 | 1.000 | conc |
| o458 | 1025.49 | 1.000 | conc |
| o459 | 1485.10 | 1.000 | conc |
| o460 | 2173.72 | 1.000 | conc |
| o461 | 2817.71 | 1.000 | conc |
| o462 | 3158.16 | 1.000 | conc |
| o463 | 3153.43 | 1.000 | conc |
| o464 | 2927.44 | 1.000 | conc |
| o465 | 2608.26 | 1.000 | conc |
| o466 | 2318.46 | 1.000 | conc |
| o467 | 2114.00 | 1.000 | conc |
| o468 | 2006.63 | 1.000 | conc |
| o469 | 2007.23 | 1.000 | conc |
| o470 | 2111.33 | 1.000 | conc |
| o471 | 2274.46 | 1.000 | conc |
| o472 | 2445.72 | 1.000 | conc |
| o473 | 2576.12 | 1.000 | conc |
| o474 | 2641.08 | 1.000 | conc |
| o475 | 2633.07 | 1.000 | conc |
| o476 | 2544.91 | 1.000 | conc |
| o477 | 2374.42 | 1.000 | conc |
| o478 | 2172.82 | 1.000 | conc |
| o479 | 2023.83 | 1.000 | conc |
| o480 | 2011.43 | 1.000 | conc |
| o481 | 2162.14 | 1.000 | conc |
| o482 | 2366.61 | 1.000 | conc |
| o483 | 2495.85 | 1.000 | conc |
| o484 | 2434.78 | 1.000 | conc |
| o485 | 2228.21 | 1.000 | conc |
| o486 | 2023.19 | 1.000 | conc |
| o487 | 1968.43 | 1.000 | conc |
| o488 | 2188.95 | 1.000 | conc |
| o489 | 2582.80 | 1.000 | conc |
| o490 | 2921.79 | 1.000 | conc |
| o491 | 2977.08 | 1.000 | conc |

```
 o492                    2650.13          1.000        conc
 o493                    2123.20          1.000        conc
 o494                    1615.08          1.000        conc
 o495                    1326.27          1.000        conc
 o496                    1270.57          1.000        conc
 o497                    1352.40          1.000        conc
 o498                    1474.85          1.000        conc
 o499                    1549.54          1.000        conc
 o500                    1561.66          1.000        conc


Control settings:-

   Initial lambda                                               :  10.000
   Lambda adjustment factor                                     :  2.0000
   Sufficient new/old phi ratio per optimisation iteration      : 0.30000
   Limiting relative phi reduction between lambdas              : 1.00000E-02
   Maximum trial lambdas per iteration                          :   8
   Forgive model run failure during lamda testing               : yes
   Forgive model run failure during Jacobian runs               : yes

   Perform Broyden's update of Jacobian matrix                  : no
   Undertake observation re-referencing                         : no

   Maximum  factor  parameter change (factor-limited changes)   :  10.000
   Maximum relative parameter change (relative-limited changes) : na
   Fraction of initial parameter values used in computing
   change limit for near-zero parameters                        : 1.00000E-03
   Allow bending of parameter upgrade vector                    : no
   Allow parameters to stick to their bounds                    : no

   Relative phi reduction below which to begin use of
   central derivatives                                          : 0.10000
   Iteration at which to first consider derivatives switch      :   1

   Relative phi reduction indicating convergence                : 0.50000E-02
   Number of phi values required within this range              :   4
   Maximum number of consecutive failures to lower phi          :   4
   Minimal relative parameter change indicating convergence     : 0.50000E-02
   Number of consecutive iterations with minimal param change   :   4
   Maximum number of optimisation iterations                    : 200

   Attempt automatic user intervention                          : no

   Attempt reuse of parameter sensitivities                     : no

   Scale parameters by their bounds                             : yes


File saving options: -

   Save best JCO file                                           : yes
   Save multiple JCO files                                      : no
   Save multiple REI files                                      : no
   Save multiple PAR files                                      : yes


                             OPTIMISATION RECORD


INITIAL CONDITIONS:
   Sum of squared weighted residuals (ie phi) =  4.78192E+14

      Current parameter values
      h1                 33.0000
      s1                 510.000
      p1                0.800000
      o1                7.500000E-04


OPTIMISATION ITERATION NO.        : 1
   Model calls so far             : 1
   Derivative model calls so far  : 0
```

```
   Starting phi for this iteration:  4.78192E+14

       Lambda =   10.000     ----->
          Phi =  3.79929E+14  (  0.795 of starting phi)

       Lambda =   5.0000     ----->
          Phi =  3.79929E+14  (  0.795 of starting phi)

   No more lambdas: relative phi reduction between lambdas less than 0.0100
   Lowest phi this iteration:  3.79929E+14

      Current parameter values                 Previous parameter values
      h1                34.4779                h1                33.0000
      s1                1532.35                s1                510.000
      p1               0.810216                p1               0.800000
      o1               4.496110E-04            o1               7.500000E-04
   Maximum   factor change:  3.005     ["s1"]
   Maximum relative change:  2.005     ["s1"]


OPTIMISATION ITERATION NO.        : 2
   Model calls so far             : 5
   Derivative model calls so far  : 1
   Starting phi for this iteration:  3.79929E+14

       Lambda =   5.0000     ----->
          Phi =  2.22370E+14  (  0.585 of starting phi)

       Lambda =   2.5000     ----->
          Phi =  2.22370E+14  (  0.585 of starting phi)

   No more lambdas: relative phi reduction between lambdas less than 0.0100
   Lowest phi this iteration:  2.22370E+14

      Current parameter values                 Previous parameter values
      h1                33.7862                h1                34.4779
      s1                1396.30                s1                1532.35
      p1               0.800007                p1               0.810216
      o1               5.708461E-04            o1               4.496110E-04
   Maximum   factor change:  1.270     ["o1"]
   Maximum relative change: 0.2696     ["o1"]


OPTIMISATION ITERATION NO.        : 3
   Model calls so far             : 9
   Derivative model calls so far  : 2
   Starting phi for this iteration:  2.22370E+14

       Lambda =   2.5000     ----->
          Phi =  2.08110E+14  (  0.936 of starting phi)

       Lambda =   1.2500     ----->
          Phi =  2.08110E+14  (  0.936 of starting phi)

   No more lambdas: relative phi reduction between lambdas less than 0.0100
   Lowest phi this iteration:  2.08110E+14
   Relative phi reduction between optimisation iterations less than 0.1000
   Switch to higher order derivatives calculation

      Current parameter values                 Previous parameter values
      h1                33.6670                h1                33.7862
      s1                1782.84                s1                1396.30
      p1               0.795725                p1               0.800007
      o1               5.561629E-04            o1               5.708461E-04
   Maximum   factor change:  1.277     ["s1"]
   Maximum relative change: 0.2768     ["s1"]


OPTIMISATION ITERATION NO.        : 4
   Model calls so far             : 13
   Derivative model calls so far  : 3
   Starting phi for this iteration:  2.08110E+14
```

```
         Lambda =   1.2500     ----->
            Phi =  2.05750E+14  (  0.989 of starting phi)

         Lambda =  0.62500     ----->
            Phi =  2.05750E+14  (  0.989 of starting phi)

     No more lambdas: relative phi reduction between lambdas less than 0.0100
     Lowest phi this iteration:  2.05750E+14

        Current parameter values                 Previous parameter values
        h1                33.7425                h1                33.6670
        s1                1609.73                s1                1782.84
        p1               0.796664                p1               0.795725
        o1               5.389217E-04            o1               5.561629E-04
     Maximum   factor change:  1.108     ["s1"]
     Maximum relative change: 9.7098E-02 ["s1"]


  OPTIMISATION ITERATION NO.        : 5
     Model calls so far             : 19
     Derivative model calls so far  : 4
     Starting phi for this iteration:  2.05750E+14

         Lambda =  0.62500     ----->
            Phi =  2.05663E+14  (  1.000 of starting phi)

         Lambda =  0.31250     ----->
            Phi =  2.05663E+14  (  1.000 of starting phi)

     No more lambdas: relative phi reduction between lambdas less than 0.0100
     Lowest phi this iteration:  2.05663E+14

        Current parameter values                 Previous parameter values
        h1                33.7443                h1                33.7425
        s1                1641.24                s1                1609.73
        p1               0.796750                p1               0.796664
        o1               5.391827E-04            o1               5.389217E-04
     Maximum   factor change:  1.020     ["s1"]
     Maximum relative change: 1.9577E-02 ["s1"]


  OPTIMISATION ITERATION NO.        : 6
     Model calls so far             : 25
     Derivative model calls so far  : 5
     Starting phi for this iteration:  2.05663E+14

         Lambda =  0.31250     ----->
            Phi =  2.05637E+14  (  1.000 of starting phi)

         Lambda =  0.15625     ----->
            Phi =  2.05637E+14  (  1.000 of starting phi)

     No more lambdas: relative phi reduction between lambdas less than 0.0100
     Lowest phi this iteration:  2.05637E+14

        Current parameter values                 Previous parameter values
        h1                33.7492                h1                33.7443
        s1                1635.36                s1                1641.24
        p1               0.796770                p1               0.796750
        o1               5.381239E-04            o1               5.391827E-04
     Maximum   factor change:  1.004     ["s1"]
     Maximum relative change: 3.5811E-03 ["s1"]


  OPTIMISATION ITERATION NO.        : 7
     Model calls so far             : 31
     Derivative model calls so far  : 6
     Starting phi for this iteration:  2.05637E+14

         Lambda =  0.15625     ----->
            Phi =  2.05640E+14  (  1.000 times starting phi)

         Lambda =  7.81250E-02 ----->
```

```
            Phi =  2.05640E+14  (  1.000 times starting phi)

     No more lambdas: relative phi reduction between lambdas less than 0.0100
     Lowest phi this iteration:  2.05640E+14

        Current parameter values                 Previous parameter values
        h1                33.7485                h1                33.7492
        s1                1638.13                s1                1635.36
        p1               0.796763                p1               0.796770
        o1               5.383118E-04            o1               5.381239E-04
     Maximum   factor change:  1.002     ["s1"]
     Maximum relative change: 1.6892E-03 ["s1"]

     Optimisation complete: the  4 lowest phi's are within a relative distance
                            of eachother of 5.000E-03
     Total model calls:     37

     The model has been run one final time using best parameters.
     Thus all model input files contain best parameter values, and model
     output files contain model results based on these parameters.


                               OPTIMISATION RESULTS

  Covariance matrix and parameter confidence intervals cannot be determined:-
  Memory conservation is operative so that covariance matrix is not calculated.


  Parameters ----->

  Parameter      Estimated value
   h1              33.7492
   s1              1635.36
   p1             0.796770
   o1             5.381239E-04

  See file mfit.sen for parameter sensitivities.


  Observations ----->

  Observation              Measured       Calculated     Residual       Weight     Group
                           value          value
  o1                      5.186150E-16    0.00000       5.186150E-16    1.000
  conc
  o2                      6.614330E-16   3.911353-315   6.614330E-16    1.000
  conc
  o3                      6.755630E-16   5.911513-284   6.755630E-16    1.000
  conc
  o4                      2.107200E-14   2.340870-256   2.107200E-14    1.000
  conc
  o5                       0.00000       9.636611-232  -9.636611-232    1.000
  conc
  o6                      3.543900E-13   1.078024-209   3.543900E-13    1.000
  conc
  o7                       0.00000       7.218103-190  -7.218103-190    1.000
  conc
  o8                      4.614550E-12   5.568550-172   4.614550E-12    1.000
  conc
  o9                       0.00000       8.560173-156  -8.560173-156    1.000
  conc
  o10                     4.829760E-11   4.160521-141   4.829760E-11    1.000
  conc
  o11                      0.00000       9.460560-128  -9.460560-128    1.000
  conc
  o12                     3.682830E-10   1.406593-115   3.682830E-10    1.000
  conc
  o13                     2.847080E-10   1.823221-104   2.847080E-10    1.000
  conc
  o14                     1.012680E-09   2.641774E-94   1.012680E-09    1.000
  conc
  o15                     1.234790E-08   5.310304E-85   1.234790E-08    1.000
  conc
```

| | | | | | |
|---|---|---|---|---|---|
| 862 | o16 conc | 0.00000 | 1.787984E-76 | -1.787984E-76 | 1.000 |
| 863 | o17 conc | 2.207160E-07 | 1.189573E-68 | 2.207160E-07 | 1.000 |
| 864 | o18 conc | 0.00000 | 1.808766E-61 | -1.808766E-61 | 1.000 |
| 865 | o19 conc | 2.982970E-06 | 7.147952E-55 | 2.982970E-06 | 1.000 |
| 866 | o20 conc | 0.00000 | 8.228708E-49 | -8.228708E-49 | 1.000 |
| 867 | o21 conc | 3.844780E-05 | 3.054487E-43 | 3.844780E-05 | 1.000 |
| 868 | o22 conc | 1.332810E-04 | 4.003104E-38 | 1.332810E-04 | 1.000 |
| 869 | o23 conc | 4.524700E-05 | 2.009114E-33 | 4.524700E-05 | 1.000 |
| 870 | o24 conc | 0.00000 | 4.154133E-29 | -4.154133E-29 | 1.000 |
| 871 | o25 conc | 0.00000 | 3.779267E-25 | -3.779267E-25 | 1.000 |
| 872 | o26 conc | 3.313920E-03 | 1.605508E-21 | 3.313920E-03 | 1.000 |
| 873 | o27 conc | 4.709680E-03 | 3.361158E-18 | 4.709680E-03 | 1.000 |
| 874 | o28 conc | 3.738630E-03 | 3.641499E-15 | 3.738630E-03 | 1.000 |
| 875 | o29 conc | 0.00000 | 2.134619E-12 | -2.134619E-12 | 1.000 |
| 876 | o30 conc | 0.795515 | 7.050911E-10 | 0.795515 | 1.000 |
| 877 | o31 conc | 0.00000 | 1.362001E-07 | -1.362001E-07 | 1.000 |
| 878 | o32 conc | 18.0567 | 1.591792E-05 | 18.0567 | 1.000 |
| 879 | o33 conc | 0.00000 | 1.161262E-03 | -1.161262E-03 | 1.000 |
| 880 | o34 conc | 158.617 | 5.442290E-02 | 158.563 | 1.000 |
| 881 | o35 conc | 1021.51 | 1.68244 | 1019.83 | 1.000 |
| 882 | o36 conc | 25461.2 | 35.1586 | 25426.0 | 1.000 |
| 883 | o37 conc | 54889.1 | 508.042 | 54381.1 | 1.000 |
| 884 | o38 conc | 123252. | 5184.37 | 118068. | 1.000 |
| 885 | o39 conc | 439629. | 38103.2 | 401526. | 1.000 |
| 886 | o40 conc | 997271. | 205454. | 791817. | 1.000 |
| 887 | o41 conc | 1.774430E+06 | 827102. | 947328. | 1.000 |
| 888 | o42 conc | 2.486710E+06 | 2.528157E+06 | -41447.1 | 1.000 |
| 889 | o43 conc | 4.526740E+06 | 5.965732E+06 | -1.438992E+06 | 1.000 |
| 890 | o44 conc | 1.324490E+07 | 1.105499E+07 | 2.189910E+06 | 1.000 |
| 891 | o45 conc | 1.652660E+07 | 1.639246E+07 | 134145. | 1.000 |
| 892 | o46 conc | 1.706350E+07 | 1.989503E+07 | -2.831527E+06 | 1.000 |
| 893 | o47 conc | 1.896430E+07 | 2.036653E+07 | -1.402232E+06 | 1.000 |
| 894 | o48 conc | 2.344730E+07 | 1.834823E+07 | 5.099067E+06 | 1.000 |
| 895 | o49 conc | 1.701390E+07 | 1.541077E+07 | 1.603128E+06 | 1.000 |
| 896 | o50 conc | 7.951230E+06 | 1.286894E+07 | -4.917707E+06 | 1.000 |
| 897 | o51 conc | 9.231250E+06 | 1.118894E+07 | -1.957689E+06 | 1.000 |
| 898 | o52 | 8.369040E+06 | 1.022259E+07 | -1.853554E+06 | 1.000 |

| | | | | | | |
|---|---|---|---|---|---|---|
| | | | | | | conc |
| 899 | o53 | 7.774150E+06 | 9.658084E+06 | -1.883934E+06 | 1.000 | conc |
| 900 | o54 | 1.047680E+07 | 9.266509E+06 | 1.210291E+06 | 1.000 | conc |
| 901 | o55 | 1.058310E+07 | 8.935110E+06 | 1.647990E+06 | 1.000 | conc |
| 902 | o56 | 7.333510E+06 | 8.621998E+06 | -1.288488E+06 | 1.000 | conc |
| 903 | o57 | 6.669610E+06 | 8.315177E+06 | -1.645567E+06 | 1.000 | conc |
| 904 | o58 | 5.939640E+06 | 8.012312E+06 | -2.072672E+06 | 1.000 | conc |
| 905 | o59 | 9.578960E+06 | 7.713532E+06 | 1.865428E+06 | 1.000 | conc |
| 906 | o60 | 1.000890E+07 | 7.419419E+06 | 2.589481E+06 | 1.000 | conc |
| 907 | o61 | 9.376560E+06 | 7.130564E+06 | 2.245996E+06 | 1.000 | conc |
| 908 | o62 | 1.006980E+07 | 6.847483E+06 | 3.222317E+06 | 1.000 | conc |
| 909 | o63 | 4.971070E+06 | 6.570614E+06 | -1.599544E+06 | 1.000 | conc |
| 910 | o64 | 2.447770E+06 | 6.300320E+06 | -3.852550E+06 | 1.000 | conc |
| 911 | o65 | 3.784400E+06 | 6.036897E+06 | -2.252497E+06 | 1.000 | conc |
| 912 | o66 | 9.389320E+06 | 5.780581E+06 | 3.608739E+06 | 1.000 | conc |
| 913 | o67 | 1.016350E+07 | 5.531553E+06 | 4.631947E+06 | 1.000 | conc |
| 914 | o68 | 5.345640E+06 | 5.289944E+06 | 55695.9 | 1.000 | conc |
| 915 | o69 | 5.859810E+06 | 5.055841E+06 | 803969. | 1.000 | conc |
| 916 | o70 | 5.246670E+06 | 4.829291E+06 | 417379. | 1.000 | conc |
| 917 | o71 | 6.640150E+06 | 4.610306E+06 | 2.029844E+06 | 1.000 | conc |
| 918 | o72 | 5.332380E+06 | 4.398868E+06 | 933512. | 1.000 | conc |
| 919 | o73 | 3.356340E+06 | 4.194930E+06 | -838590. | 1.000 | conc |
| 920 | o74 | 2.774270E+06 | 3.998421E+06 | -1.224151E+06 | 1.000 | conc |
| 921 | o75 | 2.385890E+06 | 3.809249E+06 | -1.423359E+06 | 1.000 | conc |
| 922 | o76 | 2.952400E+06 | 3.627303E+06 | -674903. | 1.000 | conc |
| 923 | o77 | 2.057460E+06 | 3.452457E+06 | -1.394997E+06 | 1.000 | conc |
| 924 | o78 | 1.584830E+06 | 3.284572E+06 | -1.699742E+06 | 1.000 | conc |
| 925 | o79 | 2.916260E+06 | 3.123496E+06 | -207236. | 1.000 | conc |
| 926 | o80 | 2.096230E+06 | 2.969069E+06 | -872839. | 1.000 | conc |
| 927 | o81 | 2.504470E+06 | 2.821122E+06 | -316652. | 1.000 | conc |
| 928 | o82 | 1.829420E+06 | 2.679481E+06 | -850061. | 1.000 | conc |
| 929 | o83 | 830066. | 2.543967E+06 | -1.713901E+06 | 1.000 | conc |
| 930 | o84 | 2.518770E+06 | 2.414397E+06 | 104373. | 1.000 | conc |
| 931 | o85 | 2.285180E+06 | 2.290586E+06 | -5405.54 | 1.000 | conc |
| 932 | o86 | 1.772680E+06 | 2.172347E+06 | -399667. | 1.000 | conc |
| 933 | o87 | 1.961070E+06 | 2.059495E+06 | -98424.8 | 1.000 | conc |
| 934 | o88 | 1.718450E+06 | 1.951842E+06 | -233392. | 1.000 | conc |

| | | | | | | |
|---|---|---|---|---|---|---|
| 935 | o89 | conc | 1.663840E+06 | 1.849204E+06 | -185364. | 1.000 |
| 936 | o90 | conc | 1.764140E+06 | 1.751397E+06 | 12742.9 | 1.000 |
| 937 | o91 | conc | 1.492310E+06 | 1.658240E+06 | -165930. | 1.000 |
| 938 | o92 | conc | 1.283080E+06 | 1.569554E+06 | -286474. | 1.000 |
| 939 | o93 | conc | 1.473610E+06 | 1.485164E+06 | -11553.6 | 1.000 |
| 940 | o94 | conc | 1.738380E+06 | 1.404897E+06 | 333483. | 1.000 |
| 941 | o95 | conc | 1.675570E+06 | 1.328586E+06 | 346984. | 1.000 |
| 942 | o96 | conc | 1.604000E+06 | 1.256067E+06 | 347933. | 1.000 |
| 943 | o97 | conc | 889930. | 1.187179E+06 | -297249. | 1.000 |
| 944 | o98 | conc | 0.00000 | 1.121767E+06 | -1.121767E+06 | 1.000 |
| 945 | o99 | conc | 0.00000 | 1.059679E+06 | -1.059679E+06 | 1.000 |
| 946 | o100 | conc | 955486. | 1.000770E+06 | -45283.6 | 1.000 |
| 947 | o101 | conc | 1.738130E+06 | 944895. | 793235. | 1.000 |
| 948 | o102 | conc | 310564. | 891920. | -581356. | 1.000 |
| 949 | o103 | conc | 0.00000 | 841709. | -841709. | 1.000 |
| 950 | o104 | conc | 202705. | 794136. | -591431. | 1.000 |
| 951 | o105 | conc | 166223. | 749077. | -582854. | 1.000 |
| 952 | o106 | conc | 110561. | 706412. | -595851. | 1.000 |
| 953 | o107 | conc | 86750.9 | 666027. | -579276. | 1.000 |
| 954 | o108 | conc | 80882.2 | 627813. | -546931. | 1.000 |
| 955 | o109 | conc | 89213.7 | 591663. | -502449. | 1.000 |
| 956 | o110 | conc | 113375. | 557475. | -444100. | 1.000 |
| 957 | o111 | conc | 136223. | 525154. | -388931. | 1.000 |
| 958 | o112 | conc | 138918. | 494605. | -355687. | 1.000 |
| 959 | o113 | conc | 121682. | 465738. | -344056. | 1.000 |
| 960 | o114 | conc | 121917. | 438470. | -316553. | 1.000 |
| 961 | o115 | conc | 140363. | 412718. | -272355. | 1.000 |
| 962 | o116 | conc | 138477. | 388404. | -249927. | 1.000 |
| 963 | o117 | conc | 118625. | 365453. | -246828. | 1.000 |
| 964 | o118 | conc | 103562. | 343795. | -240233. | 1.000 |
| 965 | o119 | conc | 101237. | 323361. | -222124. | 1.000 |
| 966 | o120 | conc | 107748. | 304087. | -196339. | 1.000 |
| 967 | o121 | conc | 102626. | 285912. | -183286. | 1.000 |
| 968 | o122 | conc | 75468.0 | 268776. | -193308. | 1.000 |
| 969 | o123 | conc | 61503.4 | 252624. | -191121. | 1.000 |
| 970 | o124 | conc | 76858.3 | 237403. | -160545. | 1.000 |
| 971 | o125 | | 83796.5 | 223062. | -139265. | 1.000 |

| | | | | | |
|---|---|---|---|---|---|
| | conc | | | | |
| 972 | o126 conc | 75864.9 | 209553. | -133688. | 1.000 |
| 973 | o127 conc | 78627.1 | 196830. | -118203. | 1.000 |
| 974 | o128 conc | 82993.1 | 184851. | -101858. | 1.000 |
| 975 | o129 conc | 70929.6 | 173573. | -102644. | 1.000 |
| 976 | o130 conc | 61300.5 | 162959. | -101658. | 1.000 |
| 977 | o131 conc | 66857.4 | 152970. | -86112.6 | 1.000 |
| 978 | o132 conc | 70288.6 | 143572. | -73283.6 | 1.000 |
| 979 | o133 conc | 58739.3 | 134732. | -75992.5 | 1.000 |
| 980 | o134 conc | 46363.6 | 126417. | -80053.8 | 1.000 |
| 981 | o135 conc | 48146.6 | 118599. | -70452.4 | 1.000 |
| 982 | o136 conc | 69423.2 | 111248. | -41825.2 | 1.000 |
| 983 | o137 conc | 90789.1 | 104339. | -13549.8 | 1.000 |
| 984 | o138 conc | 78851.6 | 97845.0 | -18993.4 | 1.000 |
| 985 | o139 conc | 55522.4 | 91742.9 | -36220.5 | 1.000 |
| 986 | o140 conc | 56299.4 | 86009.7 | -29710.3 | 1.000 |
| 987 | o141 conc | 72537.0 | 80624.2 | -8087.19 | 1.000 |
| 988 | o142 conc | 86973.6 | 75566.0 | 11407.6 | 1.000 |
| 989 | o143 conc | 84790.9 | 70816.0 | 13974.9 | 1.000 |
| 990 | o144 conc | 62089.3 | 66356.2 | -4266.87 | 1.000 |
| 991 | o145 conc | 47548.6 | 62169.4 | -14620.8 | 1.000 |
| 992 | o146 conc | 52955.3 | 58239.5 | -5284.22 | 1.000 |
| 993 | o147 conc | 55351.2 | 54551.4 | 799.816 | 1.000 |
| 994 | o148 conc | 51979.7 | 51090.6 | 889.077 | 1.000 |
| 995 | o149 conc | 55427.2 | 47843.7 | 7583.51 | 1.000 |
| 996 | o150 conc | 53199.8 | 44797.8 | 8401.99 | 1.000 |
| 997 | o151 conc | 33000.8 | 41940.9 | -8940.14 | 1.000 |
| 998 | o152 conc | 18162.2 | 39261.7 | -21099.5 | 1.000 |
| 999 | o153 conc | 22739.4 | 36749.5 | -14010.1 | 1.000 |
| 1000 | o154 conc | 33621.3 | 34394.1 | -772.809 | 1.000 |
| 1001 | o155 conc | 39197.2 | 32186.1 | 7011.09 | 1.000 |
| 1002 | o156 conc | 39125.2 | 30116.5 | 9008.66 | 1.000 |
| 1003 | o157 conc | 35025.4 | 28177.0 | 6848.42 | 1.000 |
| 1004 | o158 conc | 28518.2 | 26359.5 | 2158.71 | 1.000 |
| 1005 | o159 conc | 21223.7 | 24656.6 | -3432.90 | 1.000 |
| 1006 | o160 conc | 14762.2 | 23061.3 | -8299.10 | 1.000 |
| 1007 | o161 conc | 10753.4 | 21567.0 | -10813.6 | 1.000 |

| | | | | | |
|---|---|---|---|---|---|
| 1008 | o162 conc | 10066.4 | 20167.4 | -10101.0 | 1.000 |
| 1009 | o163 conc | 11344.4 | 18856.7 | -7512.33 | 1.000 |
| 1010 | o164 conc | 13039.5 | 17629.5 | -4589.96 | 1.000 |
| 1011 | o165 conc | 14764.0 | 16480.4 | -1716.43 | 1.000 |
| 1012 | o166 conc | 16068.4 | 15404.8 | 663.635 | 1.000 |
| 1013 | o167 conc | 15455.8 | 14397.9 | 1057.90 | 1.000 |
| 1014 | o168 conc | 14663.3 | 13455.6 | 1207.75 | 1.000 |
| 1015 | o169 conc | 19091.1 | 12573.7 | 6517.42 | 1.000 |
| 1016 | o170 conc | 24352.6 | 11748.5 | 12604.1 | 1.000 |
| 1017 | o171 conc | 21494.7 | 10976.4 | 10518.3 | 1.000 |
| 1018 | o172 conc | 14671.8 | 10254.2 | 4417.64 | 1.000 |
| 1019 | o173 conc | 10655.3 | 9578.53 | 1076.77 | 1.000 |
| 1020 | o174 conc | 10844.5 | 8946.61 | 1897.89 | 1.000 |
| 1021 | o175 conc | 14963.9 | 8355.63 | 6608.27 | 1.000 |
| 1022 | o176 conc | 19019.4 | 7802.99 | 11216.4 | 1.000 |
| 1023 | o177 conc | 19892.0 | 7286.26 | 12605.7 | 1.000 |
| 1024 | o178 conc | 19083.0 | 6803.15 | 12279.8 | 1.000 |
| 1025 | o179 conc | 18695.9 | 6351.53 | 12344.4 | 1.000 |
| 1026 | o180 conc | 20563.9 | 5929.38 | 14634.5 | 1.000 |
| 1027 | o181 conc | 24196.4 | 5534.82 | 18661.6 | 1.000 |
| 1028 | o182 conc | 27794.8 | 5166.09 | 22628.7 | 1.000 |
| 1029 | o183 conc | 30310.9 | 4821.51 | 25489.4 | 1.000 |
| 1030 | o184 conc | 31421.3 | 4499.55 | 26921.7 | 1.000 |
| 1031 | o185 conc | 31982.9 | 4198.75 | 27784.2 | 1.000 |
| 1032 | o186 conc | 31860.6 | 3917.73 | 27942.9 | 1.000 |
| 1033 | o187 conc | 25884.9 | 3655.24 | 22229.7 | 1.000 |
| 1034 | o188 conc | 13221.5 | 3410.06 | 9811.44 | 1.000 |
| 1035 | o189 conc | 4834.01 | 3181.07 | 1652.94 | 1.000 |
| 1036 | o190 conc | 5004.64 | 2967.23 | 2037.41 | 1.000 |
| 1037 | o191 conc | 9007.62 | 2767.55 | 6240.07 | 1.000 |
| 1038 | o192 conc | 11966.3 | 2581.11 | 9385.19 | 1.000 |
| 1039 | o193 conc | 12294.3 | 2407.05 | 9887.25 | 1.000 |
| 1040 | o194 conc | 22618.7 | 2244.56 | 20374.1 | 1.000 |
| 1041 | o195 conc | 46542.1 | 2092.88 | 44449.2 | 1.000 |
| 1042 | o196 conc | 59116.4 | 1951.30 | 57165.1 | 1.000 |
| 1043 | o197 conc | 54086.6 | 1819.17 | 52267.4 | 1.000 |
| 1044 | o198 | 49842.9 | 1695.87 | 48147.0 | 1.000 |

| | | | | | |
|---|---|---|---|---|---|
| | conc | | | | |
| 1045 | o199 conc | 49878.8 | 1580.81 | 48298.0 | 1.000 |
| 1046 | o200 conc | 48086.8 | 1473.45 | 46613.4 | 1.000 |
| 1047 | o201 conc | 45371.6 | 1373.28 | 43998.3 | 1.000 |
| 1048 | o202 conc | 48500.7 | 1279.83 | 47220.9 | 1.000 |
| 1049 | o203 conc | 52492.5 | 1192.66 | 51299.8 | 1.000 |
| 1050 | o204 conc | 49431.0 | 1111.35 | 48319.7 | 1.000 |
| 1051 | o205 conc | 45507.9 | 1035.51 | 44472.4 | 1.000 |
| 1052 | o206 conc | 45413.5 | 964.783 | 44448.7 | 1.000 |
| 1053 | o207 conc | 43509.8 | 898.825 | 42611.0 | 1.000 |
| 1054 | o208 conc | 39265.3 | 837.320 | 38428.0 | 1.000 |
| 1055 | o209 conc | 38196.0 | 779.972 | 37416.0 | 1.000 |
| 1056 | o210 conc | 39207.9 | 726.504 | 38481.4 | 1.000 |
| 1057 | o211 conc | 37626.7 | 676.658 | 36950.0 | 1.000 |
| 1058 | o212 conc | 34032.5 | 630.190 | 33402.3 | 1.000 |
| 1059 | o213 conc | 33337.7 | 586.876 | 32750.8 | 1.000 |
| 1060 | o214 conc | 36078.7 | 546.504 | 35532.2 | 1.000 |
| 1061 | o215 conc | 39600.7 | 508.877 | 39091.8 | 1.000 |
| 1062 | o216 conc | 37543.5 | 473.811 | 37069.7 | 1.000 |
| 1063 | o217 conc | 30230.7 | 441.134 | 29789.6 | 1.000 |
| 1064 | o218 conc | 31305.9 | 410.686 | 30895.2 | 1.000 |
| 1065 | o219 conc | 39101.2 | 382.315 | 38718.9 | 1.000 |
| 1066 | o220 conc | 37756.0 | 355.883 | 37400.1 | 1.000 |
| 1067 | o221 conc | 29402.1 | 331.258 | 29070.8 | 1.000 |
| 1068 | o222 conc | 25088.1 | 308.319 | 24779.8 | 1.000 |
| 1069 | o223 conc | 26564.8 | 286.951 | 26277.8 | 1.000 |
| 1070 | o224 conc | 28123.6 | 267.048 | 27856.6 | 1.000 |
| 1071 | o225 conc | 27390.2 | 248.511 | 27141.7 | 1.000 |
| 1072 | o226 conc | 24404.7 | 231.247 | 24173.5 | 1.000 |
| 1073 | o227 conc | 22055.8 | 215.171 | 21840.6 | 1.000 |
| 1074 | o228 conc | 21263.4 | 200.200 | 21063.2 | 1.000 |
| 1075 | o229 conc | 18848.2 | 186.260 | 18661.9 | 1.000 |
| 1076 | o230 conc | 16756.2 | 173.282 | 16582.9 | 1.000 |
| 1077 | o231 conc | 20731.4 | 161.198 | 20570.2 | 1.000 |
| 1078 | o232 conc | 27836.6 | 149.949 | 27686.7 | 1.000 |
| 1079 | o233 conc | 31507.7 | 139.477 | 31368.2 | 1.000 |
| 1080 | o234 conc | 32254.2 | 129.730 | 32124.5 | 1.000 |

| | | | | | |
|---|---|---|---|---|---|
| 1081 | o235 conc | 34888.8 | 120.657 | 34768.1 | 1.000 |
| 1082 | o236 conc | 36437.5 | 112.212 | 36325.3 | 1.000 |
| 1083 | o237 conc | 33037.8 | 104.353 | 32933.4 | 1.000 |
| 1084 | o238 conc | 27844.2 | 97.0390 | 27747.2 | 1.000 |
| 1085 | o239 conc | 23663.0 | 90.2330 | 23572.8 | 1.000 |
| 1086 | o240 conc | 20639.0 | 83.8999 | 20555.1 | 1.000 |
| 1087 | o241 conc | 18686.5 | 78.0072 | 18608.5 | 1.000 |
| 1088 | o242 conc | 17719.8 | 72.5247 | 17647.3 | 1.000 |
| 1089 | o243 conc | 17653.0 | 67.4239 | 17585.6 | 1.000 |
| 1090 | o244 conc | 18400.6 | 62.6788 | 18337.9 | 1.000 |
| 1091 | o245 conc | 19876.7 | 58.2646 | 19818.4 | 1.000 |
| 1092 | o246 conc | 21947.8 | 54.1585 | 21893.6 | 1.000 |
| 1093 | o247 conc | 22959.0 | 50.3393 | 22908.7 | 1.000 |
| 1094 | o248 conc | 19972.5 | 46.7871 | 19925.7 | 1.000 |
| 1095 | o249 conc | 15696.4 | 43.4834 | 15652.9 | 1.000 |
| 1096 | o250 conc | 14824.8 | 40.4109 | 14784.4 | 1.000 |
| 1097 | o251 conc | 14163.1 | 37.5538 | 14125.5 | 1.000 |
| 1098 | o252 conc | 10938.9 | 34.8969 | 10904.0 | 1.000 |
| 1099 | o253 conc | 9297.15 | 32.4265 | 9264.72 | 1.000 |
| 1100 | o254 conc | 10474.8 | 30.1295 | 10444.7 | 1.000 |
| 1101 | o255 conc | 10806.7 | 27.9938 | 10778.7 | 1.000 |
| 1102 | o256 conc | 8796.51 | 26.0084 | 8770.50 | 1.000 |
| 1103 | o257 conc | 8014.11 | 24.1626 | 7989.95 | 1.000 |
| 1104 | o258 conc | 9493.13 | 22.4467 | 9470.68 | 1.000 |
| 1105 | o259 conc | 11093.2 | 20.8517 | 11072.3 | 1.000 |
| 1106 | o260 conc | 13360.3 | 19.3692 | 13340.9 | 1.000 |
| 1107 | o261 conc | 17159.2 | 17.9913 | 17141.2 | 1.000 |
| 1108 | o262 conc | 17469.6 | 16.7106 | 17452.9 | 1.000 |
| 1109 | o263 conc | 12117.2 | 15.5204 | 12101.7 | 1.000 |
| 1110 | o264 conc | 8708.14 | 14.4143 | 8693.73 | 1.000 |
| 1111 | o265 conc | 10278.4 | 13.3864 | 10265.0 | 1.000 |
| 1112 | o266 conc | 13136.8 | 12.4313 | 13124.4 | 1.000 |
| 1113 | o267 conc | 14682.0 | 11.5438 | 14670.5 | 1.000 |
| 1114 | o268 conc | 15092.5 | 10.7192 | 15081.8 | 1.000 |
| 1115 | o269 conc | 14703.9 | 9.95306 | 14693.9 | 1.000 |
| 1116 | o270 conc | 13870.6 | 9.24129 | 13861.4 | 1.000 |
| 1117 | o271 | 14481.2 | 8.58004 | 14472.6 | 1.000 |

| | | | | | |
|---|---|---|---|---|---|
| | conc | | | | |
| 1118 | o272 conc | 17999.1 | 7.96577 | 17991.1 | 1.000 |
| 1119 | o273 conc | 20879.1 | 7.39517 | 20871.7 | 1.000 |
| 1120 | o274 conc | 20813.3 | 6.86514 | 20806.4 | 1.000 |
| 1121 | o275 conc | 20470.0 | 6.37284 | 20463.6 | 1.000 |
| 1122 | o276 conc | 20325.7 | 5.91559 | 20319.8 | 1.000 |
| 1123 | o277 conc | 17950.9 | 5.49092 | 17945.4 | 1.000 |
| 1124 | o278 conc | 13279.2 | 5.09652 | 13274.1 | 1.000 |
| 1125 | o279 conc | 10433.2 | 4.73026 | 10428.5 | 1.000 |
| 1126 | o280 conc | 10368.9 | 4.39014 | 10364.5 | 1.000 |
| 1127 | o281 conc | 10902.9 | 4.07431 | 10898.8 | 1.000 |
| 1128 | o282 conc | 10993.4 | 3.78105 | 10989.6 | 1.000 |
| 1129 | o283 conc | 10436.0 | 3.50875 | 10432.5 | 1.000 |
| 1130 | o284 conc | 9908.35 | 3.25594 | 9905.09 | 1.000 |
| 1131 | o285 conc | 9893.18 | 3.02122 | 9890.16 | 1.000 |
| 1132 | o286 conc | 10141.9 | 2.80331 | 10139.1 | 1.000 |
| 1133 | o287 conc | 10404.2 | 2.60102 | 10401.6 | 1.000 |
| 1134 | o288 conc | 10507.0 | 2.41322 | 10504.6 | 1.000 |
| 1135 | o289 conc | 10500.4 | 2.23891 | 10498.2 | 1.000 |
| 1136 | o290 conc | 10687.5 | 2.07710 | 10685.4 | 1.000 |
| 1137 | o291 conc | 11015.6 | 1.92691 | 11013.7 | 1.000 |
| 1138 | o292 conc | 11336.7 | 1.78751 | 11334.9 | 1.000 |
| 1139 | o293 conc | 11502.8 | 1.65814 | 11501.1 | 1.000 |
| 1140 | o294 conc | 11425.5 | 1.53807 | 11424.0 | 1.000 |
| 1141 | o295 conc | 11217.6 | 1.42664 | 11216.2 | 1.000 |
| 1142 | o296 conc | 11033.7 | 1.32323 | 11032.4 | 1.000 |
| 1143 | o297 conc | 11018.9 | 1.22728 | 11017.7 | 1.000 |
| 1144 | o298 conc | 11137.2 | 1.13824 | 11136.1 | 1.000 |
| 1145 | o299 conc | 11192.9 | 1.05562 | 11191.8 | 1.000 |
| 1146 | o300 conc | 10984.6 | 0.978960 | 10983.6 | 1.000 |
| 1147 | o301 conc | 10377.9 | 0.907836 | 10377.0 | 1.000 |
| 1148 | o302 conc | 9476.11 | 0.841849 | 9475.27 | 1.000 |
| 1149 | o303 conc | 8435.45 | 0.780630 | 8434.67 | 1.000 |
| 1150 | o304 conc | 7412.14 | 0.723837 | 7411.42 | 1.000 |
| 1151 | o305 conc | 6542.14 | 0.671152 | 6541.47 | 1.000 |
| 1152 | o306 conc | 5899.57 | 0.622279 | 5898.95 | 1.000 |
| 1153 | o307 conc | 5546.75 | 0.576945 | 5546.17 | 1.000 |

| | | | | | |
|---|---|---|---|---|---|
| 1154 | o308 conc | 5538.49 | 0.534895 | 5537.96 | 1.000 |
| 1155 | o309 conc | 5808.09 | 0.495892 | 5807.59 | 1.000 |
| 1156 | o310 conc | 6190.55 | 0.459718 | 6190.09 | 1.000 |
| 1157 | o311 conc | 6518.06 | 0.426167 | 6517.63 | 1.000 |
| 1158 | o312 conc | 6669.54 | 0.395052 | 6669.14 | 1.000 |
| 1159 | o313 conc | 6674.79 | 0.366195 | 6674.42 | 1.000 |
| 1160 | o314 conc | 6594.03 | 0.339435 | 6593.69 | 1.000 |
| 1161 | o315 conc | 6487.49 | 0.314620 | 6487.18 | 1.000 |
| 1162 | o316 conc | 6423.96 | 0.291609 | 6423.67 | 1.000 |
| 1163 | o317 conc | 6495.94 | 0.270272 | 6495.67 | 1.000 |
| 1164 | o318 conc | 6800.02 | 0.250488 | 6799.77 | 1.000 |
| 1165 | o319 conc | 7419.38 | 0.232145 | 7419.15 | 1.000 |
| 1166 | o320 conc | 8246.96 | 0.215138 | 8246.74 | 1.000 |
| 1167 | o321 conc | 9034.60 | 0.199370 | 9034.40 | 1.000 |
| 1168 | o322 conc | 9530.89 | 0.184751 | 9530.71 | 1.000 |
| 1169 | o323 conc | 9568.11 | 0.171199 | 9567.94 | 1.000 |
| 1170 | o324 conc | 9223.84 | 0.158636 | 9223.68 | 1.000 |
| 1171 | o325 conc | 8620.39 | 0.146990 | 8620.24 | 1.000 |
| 1172 | o326 conc | 7880.08 | 0.136195 | 7879.94 | 1.000 |
| 1173 | o327 conc | 7125.52 | 0.126189 | 7125.39 | 1.000 |
| 1174 | o328 conc | 6480.04 | 0.116914 | 6479.92 | 1.000 |
| 1175 | o329 conc | 6067.10 | 0.108317 | 6066.99 | 1.000 |
| 1176 | o330 conc | 5990.81 | 0.100349 | 5990.71 | 1.000 |
| 1177 | o331 conc | 6113.39 | 9.296461E-02 | 6113.30 | 1.000 |
| 1178 | o332 conc | 6132.63 | 8.612080E-02 | 6132.54 | 1.000 |
| 1179 | o333 conc | 5743.19 | 7.977834E-02 | 5743.11 | 1.000 |
| 1180 | o334 conc | 4692.77 | 7.390070E-02 | 4692.70 | 1.000 |
| 1181 | o335 conc | 3278.84 | 6.845399E-02 | 3278.77 | 1.000 |
| 1182 | o336 conc | 2124.16 | 6.340678E-02 | 2124.10 | 1.000 |
| 1183 | o337 conc | 1855.75 | 5.872992E-02 | 1855.69 | 1.000 |
| 1184 | o338 conc | 2987.66 | 5.439638E-02 | 2987.61 | 1.000 |
| 1185 | o339 conc | 5053.39 | 5.038109E-02 | 5053.34 | 1.000 |
| 1186 | o340 conc | 7082.62 | 4.666078E-02 | 7082.57 | 1.000 |
| 1187 | o341 conc | 8104.81 | 4.321391E-02 | 8104.77 | 1.000 |
| 1188 | o342 conc | 7667.29 | 4.002047E-02 | 7667.25 | 1.000 |
| 1189 | o343 conc | 6347.40 | 3.706192E-02 | 6347.36 | 1.000 |
| 1190 | o344 | 4844.30 | 3.432108E-02 | 4844.27 | 1.000 |

| | | | | | |
|---|---|---|---|---|---|
| | conc | | | | |
| 1191 | o345 conc | 3800.34 | 3.178200E-02 | 3800.31 | 1.000 |
| 1192 | o346 conc | 3332.00 | 2.942990E-02 | 3331.97 | 1.000 |
| 1193 | o347 conc | 3271.28 | 2.725109E-02 | 3271.25 | 1.000 |
| 1194 | o348 conc | 3447.37 | 2.523285E-02 | 3447.34 | 1.000 |
| 1195 | o349 conc | 3697.06 | 2.336341E-02 | 3697.04 | 1.000 |
| 1196 | o350 conc | 3916.26 | 2.163185E-02 | 3916.24 | 1.000 |
| 1197 | o351 conc | 4028.66 | 2.002806E-02 | 4028.64 | 1.000 |
| 1198 | o352 conc | 3958.99 | 1.854264E-02 | 3958.97 | 1.000 |
| 1199 | o353 conc | 3720.92 | 1.716691E-02 | 3720.90 | 1.000 |
| 1200 | o354 conc | 3489.71 | 1.589280E-02 | 3489.69 | 1.000 |
| 1201 | o355 conc | 3457.76 | 1.471285E-02 | 3457.75 | 1.000 |
| 1202 | o356 conc | 3786.29 | 1.362011E-02 | 3786.28 | 1.000 |
| 1203 | o357 conc | 4378.55 | 1.260819E-02 | 4378.54 | 1.000 |
| 1204 | o358 conc | 5010.24 | 1.167112E-02 | 5010.23 | 1.000 |
| 1205 | o359 conc | 5456.18 | 1.080341E-02 | 5456.17 | 1.000 |
| 1206 | o360 conc | 5519.01 | 9.999928E-03 | 5519.00 | 1.000 |
| 1207 | o361 conc | 5196.47 | 9.255954E-03 | 5196.46 | 1.000 |
| 1208 | o362 conc | 4569.70 | 8.567097E-03 | 4569.69 | 1.000 |
| 1209 | o363 conc | 3720.87 | 7.929293E-03 | 3720.86 | 1.000 |
| 1210 | o364 conc | 2783.53 | 7.338774E-03 | 2783.52 | 1.000 |
| 1211 | o365 conc | 1976.63 | 6.792051E-03 | 1976.62 | 1.000 |
| 1212 | o366 conc | 1527.18 | 6.285890E-03 | 1527.17 | 1.000 |
| 1213 | o367 conc | 1625.43 | 5.817295E-03 | 1625.42 | 1.000 |
| 1214 | o368 conc | 2187.95 | 5.383490E-03 | 2187.94 | 1.000 |
| 1215 | o369 conc | 3007.88 | 4.981903E-03 | 3007.88 | 1.000 |
| 1216 | o370 conc | 3877.77 | 4.610151E-03 | 3877.77 | 1.000 |
| 1217 | o371 conc | 4609.32 | 4.266029E-03 | 4609.32 | 1.000 |
| 1218 | o372 conc | 5135.25 | 3.947491E-03 | 5135.25 | 1.000 |
| 1219 | o373 conc | 5435.37 | 3.652643E-03 | 5435.37 | 1.000 |
| 1220 | o374 conc | 5489.98 | 3.379730E-03 | 5489.98 | 1.000 |
| 1221 | o375 conc | 5300.82 | 3.127129E-03 | 5300.82 | 1.000 |
| 1222 | o376 conc | 4902.23 | 2.893333E-03 | 4902.23 | 1.000 |
| 1223 | o377 conc | 4331.65 | 2.676948E-03 | 4331.65 | 1.000 |
| 1224 | o378 conc | 3660.98 | 2.476683E-03 | 3660.98 | 1.000 |
| 1225 | o379 conc | 3025.59 | 2.291342E-03 | 3025.59 | 1.000 |
| 1226 | o380 conc | 2567.69 | 2.119818E-03 | 2567.69 | 1.000 |

| | | | | | |
|---|---|---|---|---|---|
| 1227 | o381 conc | 2416.88 | 1.961085E-03 | 2416.88 | 1.000 |
| 1228 | o382 conc | 2596.80 | 1.814192E-03 | 2596.80 | 1.000 |
| 1229 | o383 conc | 3077.96 | 1.678260E-03 | 3077.96 | 1.000 |
| 1230 | o384 conc | 3830.52 | 1.552475E-03 | 3830.52 | 1.000 |
| 1231 | o385 conc | 4812.50 | 1.436082E-03 | 4812.50 | 1.000 |
| 1232 | o386 conc | 5895.83 | 1.328383E-03 | 5895.83 | 1.000 |
| 1233 | o387 conc | 6915.08 | 1.228731E-03 | 6915.08 | 1.000 |
| 1234 | o388 conc | 7705.17 | 1.136526E-03 | 7705.17 | 1.000 |
| 1235 | o389 conc | 8137.47 | 1.051215E-03 | 8137.47 | 1.000 |
| 1236 | o390 conc | 8144.74 | 9.722848E-04 | 8144.74 | 1.000 |
| 1237 | o391 conc | 7665.61 | 8.992590E-04 | 7665.61 | 1.000 |
| 1238 | o392 conc | 6666.14 | 8.316981E-04 | 6666.14 | 1.000 |
| 1239 | o393 conc | 5319.59 | 7.691947E-04 | 5319.59 | 1.000 |
| 1240 | o394 conc | 3893.92 | 7.113716E-04 | 3893.92 | 1.000 |
| 1241 | o395 conc | 2657.58 | 6.578796E-04 | 2657.58 | 1.000 |
| 1242 | o396 conc | 1843.08 | 6.083957E-04 | 1843.08 | 1.000 |
| 1243 | o397 conc | 1452.31 | 5.626206E-04 | 1452.31 | 1.000 |
| 1244 | o398 conc | 1396.14 | 5.202774E-04 | 1396.14 | 1.000 |
| 1245 | o399 conc | 1586.28 | 4.811098E-04 | 1586.28 | 1.000 |
| 1246 | o400 conc | 1996.92 | 4.448804E-04 | 1996.92 | 1.000 |
| 1247 | o401 conc | 2698.55 | 4.113698E-04 | 2698.55 | 1.000 |
| 1248 | o402 conc | 3769.91 | 3.803745E-04 | 3769.91 | 1.000 |
| 1249 | o403 conc | 5245.89 | 3.517066E-04 | 5245.89 | 1.000 |
| 1250 | o404 conc | 6863.28 | 3.251919E-04 | 6863.28 | 1.000 |
| 1251 | o405 conc | 8234.65 | 3.006692E-04 | 8234.65 | 1.000 |
| 1252 | o406 conc | 8972.15 | 2.779895E-04 | 8972.15 | 1.000 |
| 1253 | o407 conc | 8786.59 | 2.570148E-04 | 8786.59 | 1.000 |
| 1254 | o408 conc | 7959.86 | 2.376172E-04 | 7959.86 | 1.000 |
| 1255 | o409 conc | 6978.88 | 2.196787E-04 | 6978.88 | 1.000 |
| 1256 | o410 conc | 6322.32 | 2.030900E-04 | 6322.32 | 1.000 |
| 1257 | o411 conc | 6064.89 | 1.877497E-04 | 6064.89 | 1.000 |
| 1258 | o412 conc | 5710.94 | 1.735643E-04 | 5710.94 | 1.000 |
| 1259 | o413 conc | 4721.68 | 1.604471E-04 | 4721.68 | 1.000 |
| 1260 | o414 conc | 2762.35 | 1.483180E-04 | 2762.35 | 1.000 |
| 1261 | o415 conc | 750.199 | 1.371028E-04 | 750.199 | 1.000 |
| 1262 | o416 conc | 77.2322 | 1.267328E-04 | 77.2321 | 1.000 |
| 1263 | o417 | 2136.35 | 1.171447E-04 | 2136.35 | 1.000 |

| | | | | | | |
|---|---|---|---|---|---|---|
| | | | | | | conc |
| 1264 | o418 | 6022.50 | 1.082796E-04 | 6022.50 | 1.000 | conc |
| 1265 | o419 | 6694.25 | 1.000833E-04 | 6694.25 | 1.000 | conc |
| 1266 | o420 | 5144.10 | 9.250536E-05 | 5144.10 | 1.000 | conc |
| 1267 | o421 | 2885.38 | 8.549938E-05 | 2885.38 | 1.000 | conc |
| 1268 | o422 | 1382.02 | 7.902231E-05 | 1382.02 | 1.000 | conc |
| 1269 | o423 | 1080.33 | 7.303437E-05 | 1080.33 | 1.000 | conc |
| 1270 | o424 | 1470.63 | 6.749873E-05 | 1470.63 | 1.000 | conc |
| 1271 | o425 | 2006.22 | 6.238135E-05 | 2006.22 | 1.000 | conc |
| 1272 | o426 | 2257.46 | 5.765073E-05 | 2257.46 | 1.000 | conc |
| 1273 | o427 | 2243.07 | 5.327773E-05 | 2243.07 | 1.000 | conc |
| 1274 | o428 | 2089.08 | 4.923541E-05 | 2089.08 | 1.000 | conc |
| 1275 | o429 | 1918.42 | 4.549885E-05 | 1918.42 | 1.000 | conc |
| 1276 | o430 | 1783.93 | 4.204498E-05 | 1783.93 | 1.000 | conc |
| 1277 | o431 | 1669.31 | 3.885250E-05 | 1669.31 | 1.000 | conc |
| 1278 | o432 | 1555.32 | 3.590169E-05 | 1555.32 | 1.000 | conc |
| 1279 | o433 | 1425.76 | 3.317431E-05 | 1425.76 | 1.000 | conc |
| 1280 | o434 | 1318.85 | 3.065349E-05 | 1318.85 | 1.000 | conc |
| 1281 | o435 | 1319.69 | 2.832365E-05 | 1319.69 | 1.000 | conc |
| 1282 | o436 | 1514.93 | 2.617035E-05 | 1514.93 | 1.000 | conc |
| 1283 | o437 | 1927.13 | 2.418028E-05 | 1927.13 | 1.000 | conc |
| 1284 | o438 | 2356.48 | 2.234108E-05 | 2356.48 | 1.000 | conc |
| 1285 | o439 | 2554.99 | 2.064136E-05 | 2554.99 | 1.000 | conc |
| 1286 | o440 | 2290.19 | 1.907057E-05 | 2290.19 | 1.000 | conc |
| 1287 | o441 | 1630.71 | 1.761897E-05 | 1630.71 | 1.000 | conc |
| 1288 | o442 | 917.822 | 1.627754E-05 | 917.822 | 1.000 | conc |
| 1289 | o443 | 502.633 | 1.503794E-05 | 502.633 | 1.000 | conc |
| 1290 | o444 | 710.489 | 1.389247E-05 | 710.489 | 1.000 | conc |
| 1291 | o445 | 1465.38 | 1.283399E-05 | 1465.38 | 1.000 | conc |
| 1292 | o446 | 2374.01 | 1.185593E-05 | 2374.01 | 1.000 | conc |
| 1293 | o447 | 3034.49 | 1.095220E-05 | 3034.49 | 1.000 | conc |
| 1294 | o448 | 3179.19 | 1.011715E-05 | 3179.19 | 1.000 | conc |
| 1295 | o449 | 2963.08 | 9.345592E-06 | 2963.08 | 1.000 | conc |
| 1296 | o450 | 2624.07 | 8.632708E-06 | 2624.07 | 1.000 | conc |
| 1297 | o451 | 2389.02 | 7.974048E-06 | 2389.02 | 1.000 | conc |
| 1298 | o452 | 2299.28 | 7.365502E-06 | 2299.28 | 1.000 | conc |
| 1299 | o453 | 2242.04 | 6.803268E-06 | 2242.04 | 1.000 | conc |

| | | | | | |
|---|---|---|---|---|---|
| 1300 | o454 conc | 2099.86 | 6.283831E-06 | 2099.86 | 1.000 |
| 1301 | o455 conc | 1768.94 | 5.803943E-06 | 1768.94 | 1.000 |
| 1302 | o456 conc | 1332.86 | 5.360602E-06 | 1332.86 | 1.000 |
| 1303 | o457 conc | 1010.96 | 4.951033E-06 | 1010.96 | 1.000 |
| 1304 | o458 conc | 1025.49 | 4.572670E-06 | 1025.49 | 1.000 |
| 1305 | o459 conc | 1485.10 | 4.223143E-06 | 1485.10 | 1.000 |
| 1306 | o460 conc | 2173.72 | 3.900261E-06 | 2173.72 | 1.000 |
| 1307 | o461 conc | 2817.71 | 3.601997E-06 | 2817.71 | 1.000 |
| 1308 | o462 conc | 3158.16 | 3.326481E-06 | 3158.16 | 1.000 |
| 1309 | o463 conc | 3153.43 | 3.071982E-06 | 3153.43 | 1.000 |
| 1310 | o464 conc | 2927.44 | 2.836902E-06 | 2927.44 | 1.000 |
| 1311 | o465 conc | 2608.26 | 2.619763E-06 | 2608.26 | 1.000 |
| 1312 | o466 conc | 2318.46 | 2.419200E-06 | 2318.46 | 1.000 |
| 1313 | o467 conc | 2114.00 | 2.233950E-06 | 2114.00 | 1.000 |
| 1314 | o468 conc | 2006.63 | 2.062849E-06 | 2006.63 | 1.000 |
| 1315 | o469 conc | 2007.23 | 1.904817E-06 | 2007.23 | 1.000 |
| 1316 | o470 conc | 2111.33 | 1.758861E-06 | 2111.33 | 1.000 |
| 1317 | o471 conc | 2274.46 | 1.624059E-06 | 2274.46 | 1.000 |
| 1318 | o472 conc | 2445.72 | 1.499562E-06 | 2445.72 | 1.000 |
| 1319 | o473 conc | 2576.12 | 1.384584E-06 | 2576.12 | 1.000 |
| 1320 | o474 conc | 2641.08 | 1.278398E-06 | 2641.08 | 1.000 |
| 1321 | o475 conc | 2633.07 | 1.180336E-06 | 2633.07 | 1.000 |
| 1322 | o476 conc | 2544.91 | 1.089776E-06 | 2544.91 | 1.000 |
| 1323 | o477 conc | 2374.42 | 1.006146E-06 | 2374.42 | 1.000 |
| 1324 | o478 conc | 2172.82 | 9.289179E-07 | 2172.82 | 1.000 |
| 1325 | o479 conc | 2023.83 | 8.576025E-07 | 2023.83 | 1.000 |
| 1326 | o480 conc | 2011.43 | 7.917482E-07 | 2011.43 | 1.000 |
| 1327 | o481 conc | 2162.14 | 7.309381E-07 | 2162.14 | 1.000 |
| 1328 | o482 conc | 2366.61 | 6.747868E-07 | 2366.61 | 1.000 |
| 1329 | o483 conc | 2495.85 | 6.229382E-07 | 2495.85 | 1.000 |
| 1330 | o484 conc | 2434.78 | 5.750637E-07 | 2434.78 | 1.000 |
| 1331 | o485 conc | 2228.21 | 5.308592E-07 | 2228.21 | 1.000 |
| 1332 | o486 conc | 2023.19 | 4.900443E-07 | 2023.19 | 1.000 |
| 1333 | o487 conc | 1968.43 | 4.523598E-07 | 1968.43 | 1.000 |
| 1334 | o488 conc | 2188.95 | 4.175660E-07 | 2188.95 | 1.000 |
| 1335 | o489 conc | 2582.80 | 3.854419E-07 | 2582.80 | 1.000 |
| 1336 | o490 | 2921.79 | 3.557831E-07 | 2921.79 | 1.000 |

```
conc
o491                   2977.08       3.284010E-07    2977.08        1.000
conc
o492                   2650.13       3.031211E-07    2650.13        1.000
conc
o493                   2123.20       2.797826E-07    2123.20        1.000
conc
o494                   1615.08       2.582367E-07    1615.08        1.000
conc
o495                   1326.27       2.383460E-07    1326.27        1.000
conc
o496                   1270.57       2.199838E-07    1270.57        1.000
conc
o497                   1352.40       2.030328E-07    1352.40        1.000
conc
o498                   1474.85       1.873849E-07    1474.85        1.000
conc
o499                   1549.54       1.729402E-07    1549.54        1.000
conc
o500                   1561.66       1.596063E-07    1561.66        1.000
conc

See file mfit.res for more details of residuals in graph-ready format.

See file mfit.seo for composite observation sensitivities.


Objective function ----->

  Sum of squared weighted residuals (ie phi)                =  2.0564E+14


Correlation Coefficient ----->

  Correlation coefficient                                   =  0.96796


Analysis of residuals ----->

  All residuals:-
     Number of residuals with non-zero weight                       =   500
     Mean value of non-zero weighted residuals                      = -2.4301E+04
     Maximum weighted residual [observation "o48"]                  =  5.0991E+06
     Minimum weighted residual [observation "o50"]                  = -4.9177E+06
     Standard variance of weighted residuals                        =  4.1459E+11
     Standard error of weighted residuals                           =  6.4389E+05

     Note: the above variance was obtained by dividing the objective
     function by the number of system degrees of freedom (ie. number of
     observations with non-zero weight plus number of prior information
     articles with non-zero weight minus the number of adjustable parameters.)
     If the degrees of freedom is negative the divisor becomes
     the number of observations with non-zero weight plus the number of
     prior information items with non-zero weight.


Covariance and other statistical matrices cannot be determined:-
Memory conservation is operative so that covariance matrix is not calculated.
```
