# Supplementary material for: Comparing the Fate and Transport of MS2 Bacteriophage and Sodium Fluorescein in a Karstic Chalk Aquifer
Source: Pathogens. 2024 Feb 13;13(2):168. doi: 10.3390/pathogens13020168 (PMC10891704; doi:10.3390/pathogens13020168)
Supplement: Supplementary file 1 [file pathogens-13-00168-s001.zip › ABH2_dye_mfit.pdf]

```
                          PEST RUN RECORD: CASE mfit


PEST Version: 16.1


PEST run mode:-

   Parameter estimation mode


Case dimensions:-

   Number of parameters                              :    12
   Number of adjustable parameters                   :    12
   Number of parameter groups                        :     1
   Number of observations                            :   500
   Number of prior estimates                         :     0


Model command line(s):-

   MDP_2RNE


Jacobian command line:-

   MDP_2RNE /d
   Jacobian read from file Deriv.txt
   This is an ASCII file.


Model interface files:-

   Templates:
      MFIT.tpl
   for model input files:
      Input.txt

   (Parameter values written using single precision protocol.)
   (Decimal point always included.)

   Instruction files:
      MFIT.ins
   for reading model output files:
      Output.txt


PEST-to-model message file:-

   na


Singular value decomposition:-

   Perform SVD on XtQX or Q^(1/2)X              : Q^(1/2)X
   Max. number of singular values to employ     : 12
   Ratio of lowest/highest singular value       :  5.000000E-07
   Record eigenvectors in SVD file              : yes


Derivatives calculation:-

Param        Increment   Increment   Increment   Forward or   Multiplier  Method
group        type                    low bound   central      (central)   (central)
pgnam        relative    1.0000E-02   none         switch       1.500     parabolic


Parameter definitions:-

Name         Trans-            Change       Initial        Lower          Upper
             formation         limit        value          bound          bound
```

h1	log	factor	90.0000	80.0000	110.000
s1	log	factor	250.000	1.00000	5000.00
p1	log	factor	0.900000	0.100000	0.999000
o1	log	factor	8.000000E-05	1.000000E-10	1.000000E-02
h2	log	factor	130.000	120.000	140.000
s2	log	factor	260.000	1.00000	5000.00
p2	log	factor	0.900000	0.100000	0.999000
o2	log	factor	4.000000E-05	1.000000E-10	1.000000E-02
h3	log	factor	165.000	160.000	180.000
s3	log	factor	260.000	1.00000	5000.00
p3	log	factor	0.900000	0.100000	0.999000
o3	log	factor	5.000000E-05	1.000000E-10	1.000000E-02

Name	Group	Scale	Offset	Model command number
h1	pgnam	1.00000	0.00000	1
s1	pgnam	1.00000	0.00000	0
p1	pgnam	1.00000	0.00000	1
o1	pgnam	1.00000	0.00000	0
h2	pgnam	1.00000	0.00000	1
s2	pgnam	1.00000	0.00000	0
p2	pgnam	1.00000	0.00000	1
o2	pgnam	1.00000	0.00000	0
h3	pgnam	1.00000	0.00000	1
s3	pgnam	1.00000	0.00000	0
p3	pgnam	1.00000	0.00000	1
o3	pgnam	1.00000	0.00000	0

Prior information:-

No prior information supplied

Observations:-

Observation name	Observation	Weight	Group
o1	9.793690E-08	0.000	conc
o2	9.555730E-08	2.9875E-34	conc
o3	6.909480E-08	2.9166E-33	conc
o4	3.456580E-08	0.000	conc
o5	1.385540E-08	0.000	conc
o6	2.932160E-08	2.2916E-32	conc
o7	6.577020E-08	2.7618E-32	conc
o8	7.682880E-08	0.000	conc
o9	6.670350E-08	0.000	conc
o10	6.234490E-08	5.2774E-31	conc
o11	5.799060E-08	0.000	conc
o12	4.476690E-08	0.000	conc
o13	3.367850E-08	2.7840E-30	conc
o14	3.211560E-08	7.0866E-30	conc
o15	3.667740E-08	0.000	conc
o16	4.483250E-08	0.000	conc
o17	5.451830E-08	8.8097E-29	conc
o18	5.329870E-08	1.7567E-29	conc
o19	3.170710E-08	0.000	conc
o20	1.969980E-08	2.2803E-28	conc
o21	3.595080E-08	1.5027E-27	conc
o22	4.159740E-08	0.000	conc
o23	2.288350E-08	0.000	conc
o24	1.396180E-08	1.3218E-26	conc
o25	2.130310E-08	1.2576E-26	conc
o26	2.668720E-08	0.000	conc
o27	2.834440E-08	0.000	conc
o28	3.261680E-08	2.8365E-25	conc
o29	4.429590E-08	0.000	conc
o30	5.980420E-08	0.000	conc
o31	5.733100E-08	1.7049E-24	conc
o32	3.870850E-08	3.5219E-24	conc
o33	3.932060E-08	0.000	conc
o34	6.141800E-08	0.000	conc
o35	8.121820E-08	6.4216E-23	conc
o36	9.162970E-08	1.8034E-22	conc
o37	9.354340E-08	2.7427E-22	conc

o38	8.789140E-08	2.8007E-22	conc
o39	7.560540E-08	1.3180E-22	conc
o40	5.780150E-08	0.000	conc
o41	3.886980E-08	0.000	conc
o42	2.628710E-08	8.5834E-22	conc
o43	2.989340E-08	1.0422E-22	conc
o44	5.157320E-08	0.000	conc
o45	5.332080E-08	2.4642E-21	conc
o46	2.038440E-08	1.4128E-20	conc
o47	1.903840E-08	0.000	conc
o48	6.196760E-08	0.000	conc
o49	7.597280E-08	1.2914E-19	conc
o50	5.556080E-08	1.1076E-19	conc
o51	4.831800E-08	0.000	conc
o52	5.189860E-08	0.000	conc
o53	4.596900E-08	2.6974E-18	conc
o54	3.430070E-08	0.000	conc
o55	2.472200E-08	0.000	conc
o56	2.023650E-08	1.7009E-17	conc
o57	2.148250E-08	3.2382E-17	conc
o58	2.703960E-08	0.000	conc
o59	3.751750E-08	0.000	conc
o60	5.294450E-08	4.6745E-16	conc
o61	5.765700E-08	0.000	conc
o62	4.120220E-08	0.000	conc
o63	3.836340E-08	1.7138E-15	conc
o64	6.394200E-08	7.2934E-15	conc
o65	6.994170E-08	0.000	conc
o66	4.878650E-08	0.000	conc
o67	4.718890E-08	7.3345E-14	conc
o68	6.046870E-08	4.6697E-14	conc
o69	5.309940E-08	0.000	conc
o70	3.999120E-08	5.7380E-14	conc
o71	4.417600E-08	1.4348E-12	conc
o72	4.504670E-08	0.000	conc
o73	3.019780E-08	0.000	conc
o74	2.138340E-08	1.0126E-11	conc
o75	2.722030E-08	1.5698E-11	conc
o76	3.698210E-08	0.000	conc
o77	3.912680E-08	0.000	conc
o78	2.721060E-08	2.5531E-10	conc
o79	2.474320E-08	0.000	conc
o80	5.070650E-08	0.000	conc
o81	7.305120E-08	1.1428E-09	conc
o82	7.050620E-08	5.3571E-09	conc
o83	6.039960E-08	0.000	conc
o84	5.758830E-08	0.000	conc
o85	5.072720E-08	1.1595E-08	conc
o86	3.325280E-08	5.4377E-08	conc
o87	2.315410E-08	0.000	conc
o88	2.704570E-08	0.000	conc
o89	3.444830E-08	5.2756E-07	conc
o90	5.476300E-08	3.7702E-07	conc
o91	1.038760E-07	0.000	conc
o92	1.349690E-07	2.4252E-07	conc
o93	9.972090E-08	1.0565E-05	conc
o94	4.303290E-08	0.000	conc
o95	9.519600E-09	0.000	conc
o96	0.00000	7.1661E-05	conc
o97	2.867600E-10	1.1972E-04	conc
o98	5.113690E-10	0.000	conc
o99	3.620180E-10	0.000	conc
o100	8.644170E-09	1.8620E-03	conc
o101	1.687530E-08	0.000	conc
o102	1.559690E-08	0.000	conc
o103	1.561520E-08	7.7163E-03	conc
o104	2.199730E-08	2.7854E-02	conc
o105	2.105160E-08	0.000	conc
o106	1.351390E-08	0.000	conc
o107	1.636200E-08	0.3487	conc
o108	2.216440E-08	0.9488	conc
o109	1.374520E-08	1.102	conc
o110	1.313990E-08	0.9934	conc

220	o111	3.845720E-08	0.9771	conc
221	o112	4.944010E-08	1.005	conc
222	o113	2.832360E-08	1.004	conc
223	o114	1.708140E-08	0.9981	conc
224	o115	3.104670E-08	0.9993	conc
225	o116	5.152090E-08	1.001	conc
226	o117	6.025300E-08	1.000	conc
227	o118	4.539120E-08	0.9999	conc
228	o119	2.340680E-08	1.000	conc
229	o120	1.412990E-08	1.000	conc
230	o121	1.864530E-08	1.000	conc
231	o122	2.655290E-08	1.000	conc
232	o123	2.514160E-08	1.000	conc
233	o124	2.011030E-08	1.000	conc
234	o125	1.646220E-08	1.000	conc
235	o126	1.646350E-08	1.000	conc
236	o127	2.225790E-08	1.000	conc
237	o128	3.508680E-08	1.000	conc
238	o129	5.136760E-08	1.000	conc
239	o130	6.591230E-08	1.000	conc
240	o131	7.358170E-08	1.000	conc
241	o132	7.271480E-08	1.000	conc
242	o133	6.732970E-08	1.000	conc
243	o134	6.196880E-08	1.000	conc
244	o135	6.078950E-08	1.000	conc
245	o136	6.429350E-08	1.000	conc
246	o137	7.096050E-08	1.000	conc
247	o138	7.925050E-08	1.000	conc
248	o139	8.847530E-08	1.000	conc
249	o140	1.002400E-07	1.000	conc
250	o141	1.165300E-07	1.000	conc
251	o142	1.391270E-07	1.000	conc
252	o143	1.659020E-07	1.000	conc
253	o144	1.912470E-07	1.000	conc
254	o145	2.094310E-07	1.000	conc
255	o146	2.165920E-07	1.000	conc
256	o147	2.174470E-07	1.000	conc
257	o148	2.191720E-07	1.000	conc
258	o149	2.288320E-07	1.000	conc
259	o150	2.480410E-07	1.000	conc
260	o151	2.706290E-07	1.000	conc
261	o152	2.898290E-07	1.000	conc
262	o153	2.997530E-07	1.000	conc
263	o154	3.015680E-07	1.000	conc
264	o155	2.998420E-07	1.000	conc
265	o156	2.991550E-07	1.000	conc
266	o157	3.022960E-07	1.000	conc
267	o158	3.078700E-07	1.000	conc
268	o159	3.138870E-07	1.000	conc
269	o160	3.184520E-07	1.000	conc
270	o161	3.210780E-07	1.000	conc
271	o162	3.223800E-07	1.000	conc
272	o163	3.230020E-07	1.000	conc
273	o164	3.235000E-07	1.000	conc
274	o165	3.240740E-07	1.000	conc
275	o166	3.248360E-07	1.000	conc
276	o167	3.259000E-07	1.000	conc
277	o168	3.273780E-07	1.000	conc
278	o169	3.293840E-07	1.000	conc
279	o170	3.320300E-07	1.000	conc
280	o171	3.354300E-07	1.000	conc
281	o172	3.396930E-07	1.000	conc
282	o173	3.431300E-07	1.000	conc
283	o174	3.395130E-07	1.000	conc
284	o175	3.262010E-07	1.000	conc
285	o176	3.068900E-07	1.000	conc
286	o177	2.857790E-07	1.000	conc
287	o178	2.668110E-07	1.000	conc
288	o179	2.517910E-07	1.000	conc
289	o180	2.414640E-07	1.000	conc
290	o181	2.365650E-07	1.000	conc
291	o182	2.363110E-07	1.000	conc
292	o183	2.362840E-07	1.000	conc

o184	2.315340E-07	1.000	conc
o185	2.175590E-07	1.000	conc
o186	1.970180E-07	1.000	conc
o187	1.783120E-07	1.000	conc
o188	1.700010E-07	1.000	conc
o189	1.777150E-07	1.000	conc
o190	1.951580E-07	1.000	conc
o191	2.130090E-07	1.000	conc
o192	2.220980E-07	1.000	conc
o193	2.190210E-07	1.000	conc
o194	2.077680E-07	1.000	conc
o195	1.928170E-07	1.000	conc
o196	1.781330E-07	1.000	conc
o197	1.640790E-07	1.000	conc
o198	1.494680E-07	1.000	conc
o199	1.331190E-07	1.000	conc
o200	1.157390E-07	1.000	conc
o201	1.019720E-07	1.000	conc
o202	9.694750E-08	1.000	conc
o203	1.051370E-07	1.000	conc
o204	1.223640E-07	1.000	conc
o205	1.383770E-07	1.000	conc
o206	1.428050E-07	1.000	conc
o207	1.314920E-07	1.000	conc
o208	1.220170E-07	1.000	conc
o209	1.310930E-07	1.000	conc
o210	1.516410E-07	1.000	conc
o211	1.702820E-07	1.000	conc
o212	1.738790E-07	1.000	conc
o213	1.592060E-07	1.000	conc
o214	1.362240E-07	1.000	conc
o215	1.158070E-07	1.000	conc
o216	1.075060E-07	1.000	conc
o217	1.111280E-07	1.000	conc
o218	1.221220E-07	1.000	conc
o219	1.359230E-07	1.000	conc
o220	1.490340E-07	1.000	conc
o221	1.602590E-07	1.000	conc
o222	1.687090E-07	1.000	conc
o223	1.735440E-07	1.000	conc
o224	1.746890E-07	1.000	conc
o225	1.726160E-07	1.000	conc
o226	1.678140E-07	1.000	conc
o227	1.609850E-07	1.000	conc
o228	1.536160E-07	1.000	conc
o229	1.473760E-07	1.000	conc
o230	1.439010E-07	1.000	conc
o231	1.439110E-07	1.000	conc
o232	1.470550E-07	1.000	conc
o233	1.529210E-07	1.000	conc
o234	1.607910E-07	1.000	conc
o235	1.680200E-07	1.000	conc
o236	1.712170E-07	1.000	conc
o237	1.670130E-07	1.000	conc
o238	1.547170E-07	1.000	conc
o239	1.386780E-07	1.000	conc
o240	1.238020E-07	1.000	conc
o241	1.146300E-07	1.000	conc
o242	1.115150E-07	1.000	conc
o243	1.121430E-07	1.000	conc
o244	1.141600E-07	1.000	conc
o245	1.158380E-07	1.000	conc
o246	1.174180E-07	1.000	conc
o247	1.195250E-07	1.000	conc
o248	1.227500E-07	1.000	conc
o249	1.267810E-07	1.000	conc
o250	1.303900E-07	1.000	conc
o251	1.323070E-07	1.000	conc
o252	1.312620E-07	1.000	conc
o253	1.259960E-07	1.000	conc
o254	1.164210E-07	1.000	conc
o255	1.044990E-07	1.000	conc
o256	9.240340E-08	1.000	conc

o257	8.230520E-08	1.000	conc
o258	7.637660E-08	1.000	conc
o259	7.622740E-08	1.000	conc
o260	8.043840E-08	1.000	conc
o261	8.657490E-08	1.000	conc
o262	9.220160E-08	1.000	conc
o263	9.488300E-08	1.000	conc
o264	9.228590E-08	1.000	conc
o265	8.425190E-08	1.000	conc
o266	7.270670E-08	1.000	conc
o267	5.965960E-08	1.000	conc
o268	4.711980E-08	1.000	conc
o269	3.709570E-08	1.000	conc
o270	3.087830E-08	1.000	conc
o271	2.781440E-08	1.000	conc
o272	2.692480E-08	1.000	conc
o273	2.723000E-08	1.000	conc
o274	2.775070E-08	1.000	conc
o275	2.760460E-08	1.000	conc
o276	2.676470E-08	1.000	conc
o277	2.564830E-08	1.000	conc
o278	2.467640E-08	1.000	conc
o279	2.427000E-08	1.000	conc
o280	2.484250E-08	1.000	conc
o281	2.641960E-08	1.000	conc
o282	2.847000E-08	1.000	conc
o283	3.041950E-08	1.000	conc
o284	3.169390E-08	1.000	conc
o285	3.171880E-08	1.000	conc
o286	3.013650E-08	1.000	conc
o287	2.751750E-08	1.000	conc
o288	2.468130E-08	1.000	conc
o289	2.244710E-08	1.000	conc
o290	2.163440E-08	1.000	conc
o291	2.300960E-08	1.000	conc
o292	2.652400E-08	1.000	conc
o293	3.148730E-08	1.000	conc
o294	3.719240E-08	1.000	conc
o295	4.293170E-08	1.000	conc
o296	4.799810E-08	1.000	conc
o297	5.168400E-08	1.000	conc
o298	5.328230E-08	1.000	conc
o299	5.226800E-08	1.000	conc
o300	4.918680E-08	1.000	conc
o301	4.497330E-08	1.000	conc
o302	4.056260E-08	1.000	conc
o303	3.689010E-08	1.000	conc
o304	3.486190E-08	1.000	conc
o305	3.468440E-08	1.000	conc
o306	3.584280E-08	1.000	conc
o307	3.778920E-08	1.000	conc
o308	3.997600E-08	1.000	conc
o309	4.185520E-08	1.000	conc
o310	4.287920E-08	1.000	conc
o311	4.250540E-08	1.000	conc
o312	4.056860E-08	1.000	conc
o313	3.752820E-08	1.000	conc
o314	3.390200E-08	1.000	conc
o315	3.020810E-08	1.000	conc
o316	2.696450E-08	1.000	conc
o317	2.468900E-08	1.000	conc
o318	2.389950E-08	1.000	conc
o319	2.486570E-08	1.000	conc
o320	2.718140E-08	1.000	conc
o321	3.032590E-08	1.000	conc
o322	3.377870E-08	1.000	conc
o323	3.701890E-08	1.000	conc
o324	3.952600E-08	1.000	conc
o325	4.077930E-08	1.000	conc
o326	4.038680E-08	1.000	conc
o327	3.855690E-08	1.000	conc
o328	3.567240E-08	1.000	conc
o329	3.211630E-08	1.000	conc

o330	2.827140E-08	1.000	conc
o331	2.452070E-08	1.000	conc
o332	2.124710E-08	1.000	conc
o333	1.879250E-08	1.000	conc
o334	1.716460E-08	1.000	conc
o335	1.620760E-08	1.000	conc
o336	1.576470E-08	1.000	conc
o337	1.567900E-08	1.000	conc
o338	1.579370E-08	1.000	conc
o339	1.595200E-08	1.000	conc
o340	1.600030E-08	1.000	conc
o341	1.587080E-08	1.000	conc
o342	1.558860E-08	1.000	conc
o343	1.518340E-08	1.000	conc
o344	1.468500E-08	1.000	conc
o345	1.412300E-08	1.000	conc
o346	1.352730E-08	1.000	conc
o347	1.292740E-08	1.000	conc
o348	1.234810E-08	1.000	conc
o349	1.180590E-08	1.000	conc
o350	1.131620E-08	1.000	conc
o351	1.089450E-08	1.000	conc
o352	1.055630E-08	1.000	conc
o353	1.031710E-08	1.000	conc
o354	1.019230E-08	1.000	conc
o355	1.018390E-08	1.000	conc
o356	1.025410E-08	1.000	conc
o357	1.035830E-08	1.000	conc
o358	1.045180E-08	1.000	conc
o359	1.048990E-08	1.000	conc
o360	1.042780E-08	1.000	conc
o361	1.022100E-08	1.000	conc
o362	9.846240E-09	1.000	conc
o363	9.387050E-09	1.000	conc
o364	8.959660E-09	1.000	conc
o365	8.680350E-09	1.000	conc
o366	8.665390E-09	1.000	conc
o367	9.031040E-09	1.000	conc
o368	9.893570E-09	1.000	conc
o369	1.134860E-08	1.000	conc
o370	1.331210E-08	1.000	conc
o371	1.560750E-08	1.000	conc
o372	1.805770E-08	1.000	conc
o373	2.048540E-08	1.000	conc
o374	2.271350E-08	1.000	conc
o375	2.456470E-08	1.000	conc
o376	2.587030E-08	1.000	conc
o377	2.660560E-08	1.000	conc
o378	2.686570E-08	1.000	conc
o379	2.674930E-08	1.000	conc
o380	2.635510E-08	1.000	conc
o381	2.578180E-08	1.000	conc
o382	2.512810E-08	1.000	conc
o383	2.448710E-08	1.000	conc
o384	2.393560E-08	1.000	conc
o385	2.354760E-08	1.000	conc
o386	2.339700E-08	1.000	conc
o387	2.355770E-08	1.000	conc
o388	2.410370E-08	1.000	conc
o389	2.510890E-08	1.000	conc
o390	2.660560E-08	1.000	conc
o391	2.841940E-08	1.000	conc
o392	3.031210E-08	1.000	conc
o393	3.204560E-08	1.000	conc
o394	3.338170E-08	1.000	conc
o395	3.408220E-08	1.000	conc
o396	3.390880E-08	1.000	conc
o397	3.266310E-08	1.000	conc
o398	3.049240E-08	1.000	conc
o399	2.772300E-08	1.000	conc
o400	2.468260E-08	1.000	conc
o401	2.169880E-08	1.000	conc
o402	1.909940E-08	1.000	conc

o403	1.721200E-08	1.000	conc
o404	1.634920E-08	1.000	conc
o405	1.656890E-08	1.000	conc
o406	1.771620E-08	1.000	conc
o407	1.962940E-08	1.000	conc
o408	2.214720E-08	1.000	conc
o409	2.510800E-08	1.000	conc
o410	2.835030E-08	1.000	conc
o411	3.171240E-08	1.000	conc
o412	3.502540E-08	1.000	conc
o413	3.811070E-08	1.000	conc
o414	4.078870E-08	1.000	conc
o415	4.288030E-08	1.000	conc
o416	4.420600E-08	1.000	conc
o417	4.458640E-08	1.000	conc
o418	4.384330E-08	1.000	conc
o419	4.198300E-08	1.000	conc
o420	3.941400E-08	1.000	conc
o421	3.659730E-08	1.000	conc
o422	3.399400E-08	1.000	conc
o423	3.204760E-08	1.000	conc
o424	3.086660E-08	1.000	conc
o425	3.025930E-08	1.000	conc
o426	3.002380E-08	1.000	conc
o427	2.995800E-08	1.000	conc
o428	2.985980E-08	1.000	conc
o429	2.952710E-08	1.000	conc
o430	2.876140E-08	1.000	conc
o431	2.753560E-08	1.000	conc
o432	2.606870E-08	1.000	conc
o433	2.459860E-08	1.000	conc
o434	2.336330E-08	1.000	conc
o435	2.260070E-08	1.000	conc
o436	2.254880E-08	1.000	conc
o437	2.344480E-08	1.000	conc
o438	2.536450E-08	1.000	conc
o439	2.800460E-08	1.000	conc
o440	3.100760E-08	1.000	conc
o441	3.401610E-08	1.000	conc
o442	3.667270E-08	1.000	conc
o443	3.862000E-08	1.000	conc
o444	3.950070E-08	1.000	conc
o445	3.908620E-08	1.000	conc
o446	3.766020E-08	1.000	conc
o447	3.563300E-08	1.000	conc
o448	3.341490E-08	1.000	conc
o449	3.141630E-08	1.000	conc
o450	3.004740E-08	1.000	conc
o451	2.971880E-08	1.000	conc
o452	3.075270E-08	1.000	conc
o453	3.286720E-08	1.000	conc
o454	3.552650E-08	1.000	conc
o455	3.819390E-08	1.000	conc
o456	4.033280E-08	1.000	conc
o457	4.140650E-08	1.000	conc
o458	4.087830E-08	1.000	conc
o459	3.826280E-08	1.000	conc
o460	3.372330E-08	1.000	conc
o461	2.786850E-08	1.000	conc
o462	2.131620E-08	1.000	conc
o463	1.468380E-08	1.000	conc
o464	8.588990E-09	1.000	conc
o465	3.649360E-09	1.000	conc
o466	4.677260E-10	1.000	conc
o467	0.00000	1.000	conc
o468	0.00000	1.000	conc
o469	1.626740E-09	1.000	conc
o470	4.693200E-09	1.000	conc
o471	8.644330E-09	1.000	conc
o472	1.320690E-08	1.000	conc
o473	1.810750E-08	1.000	conc
o474	2.306840E-08	1.000	conc
o475	2.780330E-08	1.000	conc

```
 o476                    3.202510E-08      1.000       conc
 o477                    3.544690E-08      1.000       conc
 o478                    3.778170E-08      1.000       conc
 o479                    3.874230E-08      1.000       conc
 o480                    3.804200E-08      1.000       conc
 o481                    3.556310E-08      1.000       conc
 o482                    3.168540E-08      1.000       conc
 o483                    2.688000E-08      1.000       conc
 o484                    2.161760E-08      1.000       conc
 o485                    1.636920E-08      1.000       conc
 o486                    1.160560E-08      1.000       conc
 o487                    7.797780E-09      1.000       conc
 o488                    5.321320E-09      1.000       conc
 o489                    4.072160E-09      1.000       conc
 o490                    3.795720E-09      1.000       conc
 o491                    4.237380E-09      1.000       conc
 o492                    5.142490E-09      1.000       conc
 o493                    6.256440E-09      1.000       conc
 o494                    7.324600E-09      1.000       conc
 o495                    8.113290E-09      1.000       conc
 o496                    8.575080E-09      1.000       conc
 o497                    8.760030E-09      1.000       conc
 o498                    8.719080E-09      1.000       conc
 o499                    8.503150E-09      1.000       conc
 o500                    8.163170E-09      1.000       conc


Control settings:-

   Initial lambda                                               :  10.000
   Lambda adjustment factor                                     :  2.0000
   Sufficient new/old phi ratio per optimisation iteration      : 0.30000
   Limiting relative phi reduction between lambdas              : 1.00000E-02
   Maximum trial lambdas per iteration                          :   8
   Forgive model run failure during lamda testing               : yes
   Forgive model run failure during Jacobian runs               : yes

   Perform Broyden's update of Jacobian matrix                  : no
   Undertake observation re-referencing                         : no

   Maximum  factor  parameter change (factor-limited changes)   :  10.000
   Maximum relative parameter change (relative-limited changes) : na
   Fraction of initial parameter values used in computing
   change limit for near-zero parameters                        : 1.00000E-03
   Allow bending of parameter upgrade vector                    : no
   Allow parameters to stick to their bounds                    : no

   Relative phi reduction below which to begin use of
   central derivatives                                          : 0.10000
   Iteration at which to first consider derivatives switch      :   1

   Relative phi reduction indicating convergence                : 0.50000E-02
   Number of phi values required within this range              :   4
   Maximum number of consecutive failures to lower phi          :   4
   Minimal relative parameter change indicating convergence     : 0.50000E-02
   Number of consecutive iterations with minimal param change   :   4
   Maximum number of optimisation iterations                    : 200

   Attempt automatic user intervention                          : no

   Attempt reuse of parameter sensitivities                     : no

   Scale parameters by their bounds                             : yes


File saving options: -

   Save best JCO file                                           : yes
   Save multiple JCO files                                      : no
   Save multiple REI files                                      : no
   Save multiple PAR files                                      : yes
```

```
                               OPTIMISATION RECORD


INITIAL CONDITIONS:
   Sum of squared weighted residuals (ie phi) =  1.98446E-13

      Current parameter values
      h1                90.0000
      s1                250.000
      p1               0.900000
      o1               8.000000E-05
      h2                130.000
      s2                260.000
      p2               0.900000
      o2               4.000000E-05
      h3                165.000
      s3                260.000
      p3               0.900000
      o3               5.000000E-05


OPTIMISATION ITERATION NO.        : 1
   Model calls so far             : 1
   Derivative model calls so far  : 0
   Starting phi for this iteration:  1.98446E-13

       Lambda =   10.000     ----->
          Phi =  1.95988E-13  (  0.988 of starting phi)

       Lambda =   5.0000     ----->
          Phi =  1.95988E-13  (  0.988 of starting phi)

   No more lambdas: relative phi reduction between lambdas less than 0.0100
   Lowest phi this iteration:  1.95988E-13
   Relative phi reduction between optimisation iterations less than 0.1000
   Switch to higher order derivatives calculation

      Current parameter values                 Previous parameter values
      h1                90.0054                h1                90.0000
      s1                248.315                s1                250.000
      p1               0.893119                p1               0.900000
      o1               8.049647E-05            o1               8.000000E-05
      h2                130.000                h2                130.000
      s2                260.285                s2                260.000
      p2               0.900623                p2               0.900000
      o2               3.995065E-05            o2               4.000000E-05
      h3                165.000                h3                165.000
      s3                259.831                s3                260.000
      p3               0.900228                p3               0.900000
      o3               5.011740E-05            o3               5.000000E-05
   Maximum   factor change:  1.008     ["p1"]
   Maximum relative change: 7.6459E-03 ["p1"]


OPTIMISATION ITERATION NO.        : 2
   Model calls so far             : 9
   Derivative model calls so far  : 1
   Starting phi for this iteration:  1.95988E-13

       Lambda =   5.0000     ----->
          Phi =  1.95952E-13  (  1.000 of starting phi)

       Lambda =   2.5000     ----->
          Phi =  1.95952E-13  (  1.000 of starting phi)

   No more lambdas: relative phi reduction between lambdas less than 0.0100
   Lowest phi this iteration:  1.95952E-13

      Current parameter values                 Previous parameter values
      h1                90.0072                h1                90.0054
      s1                247.963                s1                248.315
      p1               0.892184                p1               0.893119
      o1               8.054990E-05            o1               8.049647E-05
```

```
      h2              129.999                 h2                130.000
      s2              260.191                 s2                260.285
      p2             0.900733                 p2               0.900623
      o2             3.992553E-05             o2               3.995065E-05
      h3              165.001                 h3                165.000
      s3              259.293                 s3                259.831
      p3             0.900920                 p3               0.900228
      o3             5.058311E-05             o3               5.011740E-05
   Maximum   factor change:  1.009     ["o3"]
   Maximum relative change: 9.2924E-03 ["o3"]


OPTIMISATION ITERATION NO.        : 3
   Model calls so far             : 23
   Derivative model calls so far  : 2
   Starting phi for this iteration:  1.95952E-13

       Lambda =   2.5000     ----->
          Phi =  1.95908E-13  (  1.000 of starting phi)

       Lambda =   1.2500     ----->
          Phi =  1.95908E-13  (  1.000 of starting phi)

   No more lambdas: relative phi reduction between lambdas less than 0.0100
   Lowest phi this iteration:  1.95908E-13

      Current parameter values                Previous parameter values
      h1              90.0080                 h1                90.0072
      s1              248.131                 s1                247.963
      p1             0.893152                 p1               0.892184
      o1             8.046042E-05             o1               8.054990E-05
      h2              129.999                 h2                129.999
      s2              259.967                 s2                260.191
      p2             0.900518                 p2               0.900733
      o2             3.989134E-05             o2               3.992553E-05
      h3              165.002                 h3                165.001
      s3              258.765                 s3                259.293
      p3             0.901591                 p3               0.900920
      o3             5.106194E-05             o3               5.058311E-05
   Maximum   factor change:  1.009     ["o3"]
   Maximum relative change: 9.4663E-03 ["o3"]


OPTIMISATION ITERATION NO.        : 4
   Model calls so far             : 37
   Derivative model calls so far  : 3
   Starting phi for this iteration:  1.95908E-13

       Lambda =   1.2500     ----->
          Phi =  1.95875E-13  (  1.000 of starting phi)

       Lambda =  0.62500     ----->
          Phi =  1.95875E-13  (  1.000 of starting phi)

   No more lambdas: relative phi reduction between lambdas less than 0.0100
   Lowest phi this iteration:  1.95875E-13

      Current parameter values                Previous parameter values
      h1              90.0097                 h1                90.0080
      s1              247.777                 s1                248.131
      p1             0.892252                 p1               0.893152
      o1             8.051378E-05             o1               8.046042E-05
      h2              129.998                 h2                129.999
      s2              259.897                 s2                259.967
      p2             0.900710                 p2               0.900518
      o2             3.987043E-05             o2               3.989134E-05
      h3              165.002                 h3                165.002
      s3              258.298                 s3                258.765
      p3             0.902186                 p3               0.901591
      o3             5.147327E-05             o3               5.106194E-05
   Maximum   factor change:  1.008     ["o3"]
   Maximum relative change: 8.0555E-03 ["o3"]
```

Optimisation complete: the 4 lowest phi's are within a relative distance of eachother of 5.000E-03

Total model calls: 51

The model has been run one final time using best parameters. Thus all model input files contain best parameter values, and model output files contain model results based on these parameters.

OPTIMISATION RESULTS

Covariance matrix and parameter confidence intervals cannot be determined:-
Memory conservation is operative so that covariance matrix is not calculated.

Parameters ----->

Parameter	Estimated value
h1	90.0097
s1	247.777
p1	0.892252
o1	8.051378E-05
h2	129.998
s2	259.897
p2	0.900710
o2	3.987043E-05
h3	165.002
s3	258.298
p3	0.902186
o3	5.147327E-05

See file mfit.sen for parameter sensitivities.

Observations ----->

Observation	Measured value	Calculated value	Residual	Weight	Group
o1	9.793690E-08	0.00000	9.793690E-08	0.000	conc
o2	9.555730E-08	0.00000	9.555730E-08	2.9875E-34	conc
o3	6.909480E-08	0.00000	6.909480E-08	2.9166E-33	conc
o4	3.456580E-08	0.00000	3.456580E-08	0.000	conc
o5	1.385540E-08	0.00000	1.385540E-08	0.000	conc
o6	2.932160E-08	0.00000	2.932160E-08	2.2916E-32	conc
o7	6.577020E-08	0.00000	6.577020E-08	2.7618E-32	conc
o8	7.682880E-08	0.00000	7.682880E-08	0.000	conc
o9	6.670350E-08	0.00000	6.670350E-08	0.000	conc
o10	6.234490E-08	0.00000	6.234490E-08	5.2774E-31	conc
o11	5.799060E-08	5.434722-322	5.799060E-08	0.000	conc
o12	4.476690E-08	2.061434-293	4.476690E-08	0.000	conc
o13	3.367850E-08	5.261114-269	3.367850E-08	2.7840E-30	conc
o14	3.211560E-08	6.347935-248	3.211560E-08	7.0866E-30	conc
o15	3.667740E-08	1.551079-229	3.667740E-08	0.000	conc
o16	4.483250E-08	2.324796-213	4.483250E-08	0.000	conc
o17	5.451830E-08	5.046382-199	5.451830E-08	8.8097E-29	conc
o18	5.329870E-08	3.119588-186	5.329870E-08	1.7567E-29	

	conc				
860	o19 conc	3.170710E-08	9.420147-175	3.170710E-08	0.000
861	o20 conc	1.969980E-08	2.148331-164	1.969980E-08	2.2803E-28
862	o21 conc	3.595080E-08	5.281148-155	3.595080E-08	1.5027E-27
863	o22 conc	4.159740E-08	1.876387-146	4.159740E-08	0.000
864	o23 conc	2.288350E-08	1.229837-138	2.288350E-08	0.000
865	o24 conc	1.396180E-08	1.824607-131	1.396180E-08	1.3218E-26
866	o25 conc	2.130310E-08	7.283986-125	2.130310E-08	1.2576E-26
867	o26 conc	2.668720E-08	9.063933-119	2.668720E-08	0.000
868	o27 conc	2.834440E-08	3.987380-113	2.834440E-08	0.000
869	o28 conc	3.261680E-08	6.911307-108	3.261680E-08	2.8365E-25
870	o29 conc	4.429590E-08	5.184299-103	4.429590E-08	0.000
871	o30 conc	5.980420E-08	1.826154E-98	5.980420E-08	0.000
872	o31 conc	5.733100E-08	3.244141E-94	5.733100E-08	1.7049E-24
873	o32 conc	3.870850E-08	3.094514E-90	3.870850E-08	3.5219E-24
874	o33 conc	3.932060E-08	1.674977E-86	3.932060E-08	0.000
875	o34 conc	6.141800E-08	5.402343E-83	6.141800E-08	0.000
876	o35 conc	8.121820E-08	1.084358E-79	8.121820E-08	6.4216E-23
877	o36 conc	9.162970E-08	1.407956E-76	9.162970E-08	1.8034E-22
878	o37 conc	9.354340E-08	1.224224E-73	9.354340E-08	2.7427E-22
879	o38 conc	8.789140E-08	7.353036E-71	8.789140E-08	2.8007E-22
880	o39 conc	7.560540E-08	3.137111E-68	7.560540E-08	1.3180E-22
881	o40 conc	5.780150E-08	9.749559E-66	5.780150E-08	0.000
882	o41 conc	3.886980E-08	2.257974E-63	3.886980E-08	0.000
883	o42 conc	2.628710E-08	3.978274E-61	2.628710E-08	8.5834E-22
884	o43 conc	2.989340E-08	5.433201E-59	2.989340E-08	1.0422E-22
885	o44 conc	5.157320E-08	5.850841E-57	5.157320E-08	0.000
886	o45 conc	5.332080E-08	5.046036E-55	5.332080E-08	2.4642E-21
887	o46 conc	2.038440E-08	3.535420E-53	2.038440E-08	1.4128E-20
888	o47 conc	1.903840E-08	2.038738E-51	1.903840E-08	0.000
889	o48 conc	6.196760E-08	9.793032E-50	6.196760E-08	0.000
890	o49 conc	7.597280E-08	3.961830E-48	7.597280E-08	1.2914E-19
891	o50 conc	5.556080E-08	1.363664E-46	5.556080E-08	1.1076E-19
892	o51 conc	4.831800E-08	4.031083E-45	4.831800E-08	0.000
893	o52 conc	5.189860E-08	1.032284E-43	5.189860E-08	0.000
894	o53 conc	4.596900E-08	2.308455E-42	4.596900E-08	2.6974E-18
895	o54 conc	3.430070E-08	4.541662E-41	3.430070E-08	0.000

896	o55 conc	2.472200E-08	7.915439E-40	2.472200E-08	0.000
897	o56 conc	2.023650E-08	1.229946E-38	2.023650E-08	1.7009E-17
898	o57 conc	2.148250E-08	1.714115E-37	2.148250E-08	3.2382E-17
899	o58 conc	2.703960E-08	2.154523E-36	2.703960E-08	0.000
900	o59 conc	3.751750E-08	2.455123E-35	3.751750E-08	0.000
901	o60 conc	5.294450E-08	2.548657E-34	5.294450E-08	4.6745E-16
902	o61 conc	5.765700E-08	2.421220E-33	5.765700E-08	0.000
903	o62 conc	4.120220E-08	2.113903E-32	4.120220E-08	0.000
904	o63 conc	3.836340E-08	1.702908E-31	3.836340E-08	1.7138E-15
905	o64 conc	6.394200E-08	1.270497E-30	6.394200E-08	7.2934E-15
906	o65 conc	6.994170E-08	8.809576E-30	6.994170E-08	0.000
907	o66 conc	4.878650E-08	5.695946E-29	4.878650E-08	0.000
908	o67 conc	4.718890E-08	3.444712E-28	4.718890E-08	7.3345E-14
909	o68 conc	6.046870E-08	1.954269E-27	6.046870E-08	4.6697E-14
910	o69 conc	5.309940E-08	1.042932E-26	5.309940E-08	0.000
911	o70 conc	3.999120E-08	5.249226E-26	3.999120E-08	5.7380E-14
912	o71 conc	4.417600E-08	2.497857E-25	4.417600E-08	1.4348E-12
913	o72 conc	4.504670E-08	1.126365E-24	4.504670E-08	0.000
914	o73 conc	3.019780E-08	4.823719E-24	3.019780E-08	0.000
915	o74 conc	2.138340E-08	1.965971E-23	2.138340E-08	1.0126E-11
916	o75 conc	2.722030E-08	7.640435E-23	2.722030E-08	1.5698E-11
917	o76 conc	3.698210E-08	2.836701E-22	3.698210E-08	0.000
918	o77 conc	3.912680E-08	1.007931E-21	3.912680E-08	0.000
919	o78 conc	2.721060E-08	3.433196E-21	2.721060E-08	2.5531E-10
920	o79 conc	2.474320E-08	1.122815E-20	2.474320E-08	0.000
921	o80 conc	5.070650E-08	3.531164E-20	5.070650E-08	0.000
922	o81 conc	7.305120E-08	1.069430E-19	7.305120E-08	1.1428E-09
923	o82 conc	7.050620E-08	3.123256E-19	7.050620E-08	5.3571E-09
924	o83 conc	6.039960E-08	8.807440E-19	6.039960E-08	0.000
925	o84 conc	5.758830E-08	2.401148E-18	5.758830E-08	0.000
926	o85 conc	5.072720E-08	6.336222E-18	5.072720E-08	1.1595E-08
927	o86 conc	3.325280E-08	1.620224E-17	3.325280E-08	5.4377E-08
928	o87 conc	2.315410E-08	4.019034E-17	2.315410E-08	0.000
929	o88 conc	2.704570E-08	9.680952E-17	2.704570E-08	0.000
930	o89 conc	3.444830E-08	2.266689E-16	3.444830E-08	5.2756E-07
931	o90 conc	5.476300E-08	5.163597E-16	5.476300E-08	3.7702E-07
932	o91	1.038760E-07	1.145487E-15	1.038760E-07	0.000

conc				
o92 conc	1.349690E-07	2.476737E-15	1.349690E-07	2.4252E-07
o93 conc	9.972090E-08	5.223721E-15	9.972089E-08	1.0565E-05
o94 conc	4.303290E-08	1.075557E-14	4.303289E-08	0.000
o95 conc	9.519600E-09	2.163563E-14	9.519578E-09	0.000
o96 conc	0.00000	4.255038E-14	-4.255038E-14	7.1661E-05
o97 conc	2.867600E-10	8.187245E-14	2.866781E-10	1.1972E-04
o98 conc	5.113690E-10	1.542279E-13	5.112148E-10	0.000
o99 conc	3.620180E-10	2.846149E-13	3.617334E-10	0.000
o100 conc	8.644170E-09	5.148604E-13	8.643655E-09	1.8620E-03
o101 conc	1.687530E-08	9.135156E-13	1.687439E-08	0.000
o102 conc	1.559690E-08	1.590685E-12	1.559531E-08	0.000
o103 conc	1.561520E-08	2.719770E-12	1.561248E-08	7.7163E-03
o104 conc	2.199730E-08	4.568656E-12	2.199273E-08	2.7854E-02
o105 conc	2.105160E-08	7.543512E-12	2.104406E-08	0.000
o106 conc	1.351390E-08	1.224897E-11	1.350165E-08	0.000
o107 conc	1.636200E-08	1.956907E-11	1.634243E-08	0.3487
o108 conc	2.216440E-08	3.077393E-11	2.213363E-08	0.9488
o109 conc	1.374520E-08	4.765706E-11	1.369754E-08	1.102
o110 conc	1.313990E-08	7.270866E-11	1.306719E-08	0.9934
o111 conc	3.845720E-08	1.093292E-10	3.834787E-08	0.9771
o112 conc	4.944010E-08	1.620868E-10	4.927801E-08	1.005
o113 conc	2.832360E-08	2.370200E-10	2.808658E-08	1.004
o114 conc	1.708140E-08	3.419853E-10	1.673941E-08	0.9981
o115 conc	3.104670E-08	4.870439E-10	3.055966E-08	0.9993
o116 conc	5.152090E-08	6.848819E-10	5.083602E-08	1.001
o117 conc	6.025300E-08	9.512475E-10	5.930175E-08	1.000
o118 conc	4.539120E-08	1.305391E-09	4.408581E-08	0.9999
o119 conc	2.340680E-08	1.770479E-09	2.163632E-08	1.000
o120 conc	1.412990E-08	2.373967E-09	1.175593E-08	1.000
o121 conc	1.864530E-08	3.147880E-09	1.549742E-08	1.000
o122 conc	2.655290E-08	4.128982E-09	2.242392E-08	1.000
o123 conc	2.514160E-08	5.358803E-09	1.978280E-08	1.000
o124 conc	2.011030E-08	6.883467E-09	1.322683E-08	1.000
o125 conc	1.646220E-08	8.753315E-09	7.708885E-09	1.000
o126 conc	1.646350E-08	1.102229E-08	5.441208E-09	1.000
o127 conc	2.225790E-08	1.374707E-08	8.510827E-09	1.000

969	o128 conc	3.508680E-08	1.698594E-08	1.810086E-08	1.000
970	o129 conc	5.136760E-08	2.079739E-08	3.057021E-08	1.000
971	o130 conc	6.591230E-08	2.523856E-08	4.067374E-08	1.000
972	o131 conc	7.358170E-08	3.036343E-08	4.321827E-08	1.000
973	o132 conc	7.271480E-08	3.622086E-08	3.649394E-08	1.000
974	o133 conc	6.732970E-08	4.285262E-08	2.447708E-08	1.000
975	o134 conc	6.196880E-08	5.029138E-08	1.167742E-08	1.000
976	o135 conc	6.078950E-08	5.855879E-08	2.230709E-09	1.000
977	o136 conc	6.429350E-08	6.766368E-08	-3.370181E-09	1.000
978	o137 conc	7.096050E-08	7.760058E-08	-6.640080E-09	1.000
979	o138 conc	7.925050E-08	8.834850E-08	-9.097998E-09	1.000
980	o139 conc	8.847530E-08	9.987012E-08	-1.139482E-08	1.000
981	o140 conc	1.002400E-07	1.121115E-07	-1.187146E-08	1.000
982	o141 conc	1.165300E-07	1.250019E-07	-8.471948E-09	1.000
983	o142 conc	1.391270E-07	1.384551E-07	6.719474E-10	1.000
984	o143 conc	1.659020E-07	1.523694E-07	1.353259E-08	1.000
985	o144 conc	1.912470E-07	1.666304E-07	2.461660E-08	1.000
986	o145 conc	2.094310E-07	1.811122E-07	2.831884E-08	1.000
987	o146 conc	2.165920E-07	1.956800E-07	2.091203E-08	1.000
988	o147 conc	2.174470E-07	2.101930E-07	7.254047E-09	1.000
989	o148 conc	2.191720E-07	2.245070E-07	-5.334960E-09	1.000
990	o149 conc	2.288320E-07	2.384776E-07	-9.645570E-09	1.000
991	o150 conc	2.480410E-07	2.519631E-07	-3.922115E-09	1.000
992	o151 conc	2.706290E-07	2.648276E-07	5.801377E-09	1.000
993	o152 conc	2.898290E-07	2.769436E-07	1.288542E-08	1.000
994	o153 conc	2.997530E-07	2.881944E-07	1.155856E-08	1.000
995	o154 conc	3.015680E-07	2.984768E-07	3.091196E-09	1.000
996	o155 conc	2.998420E-07	3.077022E-07	-7.860232E-09	1.000
997	o156 conc	2.991550E-07	3.157986E-07	-1.664361E-08	1.000
998	o157 conc	3.022960E-07	3.227111E-07	-2.041507E-08	1.000
999	o158 conc	3.078700E-07	3.284025E-07	-2.053249E-08	1.000
1000	o159 conc	3.138870E-07	3.328535E-07	-1.896653E-08	1.000
1001	o160 conc	3.184520E-07	3.360622E-07	-1.761019E-08	1.000
1002	o161 conc	3.210780E-07	3.380431E-07	-1.696514E-08	1.000
1003	o162 conc	3.223800E-07	3.388265E-07	-1.644650E-08	1.000
1004	o163 conc	3.230020E-07	3.384565E-07	-1.545455E-08	1.000
1005	o164	3.235000E-07	3.369901E-07	-1.349006E-08	1.000

	conc				
1006	o165	3.240740E-07	3.344945E-07	-1.042053E-08	1.000
	conc				
1007	o166	3.248360E-07	3.310463E-07	-6.210346E-09	1.000
	conc				
1008	o167	3.259000E-07	3.267288E-07	-8.288124E-10	1.000
	conc				
1009	o168	3.273780E-07	3.216303E-07	5.747707E-09	1.000
	conc				
1010	o169	3.293840E-07	3.158424E-07	1.354164E-08	1.000
	conc				
1011	o170	3.320300E-07	3.094580E-07	2.257196E-08	1.000
	conc				
1012	o171	3.354300E-07	3.025702E-07	3.285975E-08	1.000
	conc				
1013	o172	3.396930E-07	2.952703E-07	4.442265E-08	1.000
	conc				
1014	o173	3.431300E-07	2.876469E-07	5.548312E-08	1.000
	conc				
1015	o174	3.395130E-07	2.797845E-07	5.972848E-08	1.000
	conc				
1016	o175	3.262010E-07	2.717633E-07	5.443774E-08	1.000
	conc				
1017	o176	3.068900E-07	2.636577E-07	4.323234E-08	1.000
	conc				
1018	o177	2.857790E-07	2.555365E-07	3.024253E-08	1.000
	conc				
1019	o178	2.668110E-07	2.474622E-07	1.934876E-08	1.000
	conc				
1020	o179	2.517910E-07	2.394912E-07	1.229976E-08	1.000
	conc				
1021	o180	2.414640E-07	2.316734E-07	9.790572E-09	1.000
	conc				
1022	o181	2.365650E-07	2.240525E-07	1.251245E-08	1.000
	conc				
1023	o182	2.363110E-07	2.166664E-07	1.964462E-08	1.000
	conc				
1024	o183	2.362840E-07	2.095470E-07	2.673701E-08	1.000
	conc				
1025	o184	2.315340E-07	2.027211E-07	2.881290E-08	1.000
	conc				
1026	o185	2.175590E-07	1.962105E-07	2.134853E-08	1.000
	conc				
1027	o186	1.970180E-07	1.900323E-07	6.985718E-09	1.000
	conc				
1028	o187	1.783120E-07	1.841996E-07	-5.887595E-09	1.000
	conc				
1029	o188	1.700010E-07	1.787217E-07	-8.720711E-09	1.000
	conc				
1030	o189	1.777150E-07	1.736046E-07	4.110413E-09	1.000
	conc				
1031	o190	1.951580E-07	1.688512E-07	2.630680E-08	1.000
	conc				
1032	o191	2.130090E-07	1.644619E-07	4.854713E-08	1.000
	conc				
1033	o192	2.220980E-07	1.604346E-07	6.166340E-08	1.000
	conc				
1034	o193	2.190210E-07	1.567653E-07	6.225571E-08	1.000
	conc				
1035	o194	2.077680E-07	1.534480E-07	5.431999E-08	1.000
	conc				
1036	o195	1.928170E-07	1.504752E-07	4.234181E-08	1.000
	conc				
1037	o196	1.781330E-07	1.478377E-07	3.029525E-08	1.000
	conc				
1038	o197	1.640790E-07	1.455253E-07	1.855370E-08	1.000
	conc				
1039	o198	1.494680E-07	1.435262E-07	5.941815E-09	1.000
	conc				
1040	o199	1.331190E-07	1.418276E-07	-8.708632E-09	1.000
	conc				
1041	o200	1.157390E-07	1.404158E-07	-2.467679E-08	1.000
	conc				

1042	o201	1.019720E-07	1.392758E-07	-3.730380E-08	1.000	conc
1043	o202	9.694750E-08	1.383919E-07	-4.144437E-08	1.000	conc
1044	o203	1.051370E-07	1.377473E-07	-3.261034E-08	1.000	conc
1045	o204	1.223640E-07	1.373247E-07	-1.496068E-08	1.000	conc
1046	o205	1.383770E-07	1.371056E-07	1.271351E-09	1.000	conc
1047	o206	1.428050E-07	1.370713E-07	5.733719E-09	1.000	conc
1048	o207	1.314920E-07	1.372020E-07	-5.710016E-09	1.000	conc
1049	o208	1.220170E-07	1.374778E-07	-1.546077E-08	1.000	conc
1050	o209	1.310930E-07	1.378781E-07	-6.785063E-09	1.000	conc
1051	o210	1.516410E-07	1.383821E-07	1.325891E-08	1.000	conc
1052	o211	1.702820E-07	1.389689E-07	3.131311E-08	1.000	conc
1053	o212	1.738790E-07	1.396175E-07	3.426154E-08	1.000	conc
1054	o213	1.592060E-07	1.403069E-07	1.889910E-08	1.000	conc
1055	o214	1.362240E-07	1.410166E-07	-4.792569E-09	1.000	conc
1056	o215	1.158070E-07	1.417262E-07	-2.591922E-08	1.000	conc
1057	o216	1.075060E-07	1.424162E-07	-3.491019E-08	1.000	conc
1058	o217	1.111280E-07	1.430675E-07	-3.193951E-08	1.000	conc
1059	o218	1.221220E-07	1.436621E-07	-2.154009E-08	1.000	conc
1060	o219	1.359230E-07	1.441828E-07	-8.259832E-09	1.000	conc
1061	o220	1.490340E-07	1.446138E-07	4.420214E-09	1.000	conc
1062	o221	1.602590E-07	1.449403E-07	1.531873E-08	1.000	conc
1063	o222	1.687090E-07	1.451490E-07	2.356005E-08	1.000	conc
1064	o223	1.735440E-07	1.452280E-07	2.831601E-08	1.000	conc
1065	o224	1.746890E-07	1.451671E-07	2.952194E-08	1.000	conc
1066	o225	1.726160E-07	1.449575E-07	2.765854E-08	1.000	conc
1067	o226	1.678140E-07	1.445921E-07	2.322190E-08	1.000	conc
1068	o227	1.609850E-07	1.440656E-07	1.691943E-08	1.000	conc
1069	o228	1.536160E-07	1.433741E-07	1.024189E-08	1.000	conc
1070	o229	1.473760E-07	1.425156E-07	4.860388E-09	1.000	conc
1071	o230	1.439010E-07	1.414896E-07	2.411428E-09	1.000	conc
1072	o231	1.439110E-07	1.402970E-07	3.613954E-09	1.000	conc
1073	o232	1.470550E-07	1.389406E-07	8.114422E-09	1.000	conc
1074	o233	1.529210E-07	1.374241E-07	1.549688E-08	1.000	conc
1075	o234	1.607910E-07	1.357529E-07	2.503807E-08	1.000	conc
1076	o235	1.680200E-07	1.339335E-07	3.408652E-08	1.000	conc
1077	o236	1.712170E-07	1.319733E-07	3.924367E-08	1.000	conc
1078	o237	1.670130E-07	1.298810E-07	3.713202E-08	1.000	

	conc				
1079	o238 conc	1.547170E-07	1.276658E-07	2.705118E-08	1.000
1080	o239 conc	1.386780E-07	1.253379E-07	1.334011E-08	1.000
1081	o240 conc	1.238020E-07	1.229079E-07	8.941448E-10	1.000
1082	o241 conc	1.146300E-07	1.203868E-07	-5.756787E-09	1.000
1083	o242 conc	1.115150E-07	1.177861E-07	-6.271087E-09	1.000
1084	o243 conc	1.121430E-07	1.151173E-07	-2.974340E-09	1.000
1085	o244 conc	1.141600E-07	1.123922E-07	1.767803E-09	1.000
1086	o245 conc	1.158380E-07	1.096223E-07	6.215737E-09	1.000
1087	o246 conc	1.174180E-07	1.068190E-07	1.059902E-08	1.000
1088	o247 conc	1.195250E-07	1.039936E-07	1.553145E-08	1.000
1089	o248 conc	1.227500E-07	1.011568E-07	2.159318E-08	1.000
1090	o249 conc	1.267810E-07	9.831922E-08	2.846178E-08	1.000
1091	o250 conc	1.303900E-07	9.549069E-08	3.489931E-08	1.000
1092	o251 conc	1.323070E-07	9.268059E-08	3.962641E-08	1.000
1093	o252 conc	1.312620E-07	8.989772E-08	4.136428E-08	1.000
1094	o253 conc	1.259960E-07	8.715022E-08	3.884578E-08	1.000
1095	o254 conc	1.164210E-07	8.444553E-08	3.197547E-08	1.000
1096	o255 conc	1.044990E-07	8.179043E-08	2.270857E-08	1.000
1097	o256 conc	9.240340E-08	7.919098E-08	1.321242E-08	1.000
1098	o257 conc	8.230520E-08	7.665254E-08	5.652663E-09	1.000
1099	o258 conc	7.637660E-08	7.417974E-08	2.196861E-09	1.000
1100	o259 conc	7.622740E-08	7.177653E-08	4.450867E-09	1.000
1101	o260 conc	8.043840E-08	6.944619E-08	1.099221E-08	1.000
1102	o261 conc	8.657490E-08	6.719132E-08	1.938358E-08	1.000
1103	o262 conc	9.220160E-08	6.501390E-08	2.718770E-08	1.000
1104	o263 conc	9.488300E-08	6.291532E-08	3.196768E-08	1.000
1105	o264 conc	9.228590E-08	6.089639E-08	3.138951E-08	1.000
1106	o265 conc	8.425190E-08	5.895741E-08	2.529449E-08	1.000
1107	o266 conc	7.270670E-08	5.709818E-08	1.560852E-08	1.000
1108	o267 conc	5.965960E-08	5.531804E-08	4.341557E-09	1.000
1109	o268 conc	4.711980E-08	5.361596E-08	-6.496162E-09	1.000
1110	o269 conc	3.709570E-08	5.199052E-08	-1.489482E-08	1.000
1111	o270 conc	3.087830E-08	5.043998E-08	-1.956168E-08	1.000
1112	o271 conc	2.781440E-08	4.896231E-08	-2.114791E-08	1.000
1113	o272 conc	2.692480E-08	4.755527E-08	-2.063047E-08	1.000
1114	o273 conc	2.723000E-08	4.621638E-08	-1.898638E-08	1.000

1115	o274 conc	2.775070E-08	4.494301E-08	-1.719231E-08	1.000
1116	o275 conc	2.760460E-08	4.373240E-08	-1.612780E-08	1.000
1117	o276 conc	2.676470E-08	4.258169E-08	-1.581699E-08	1.000
1118	o277 conc	2.564830E-08	4.148794E-08	-1.583964E-08	1.000
1119	o278 conc	2.467640E-08	4.044818E-08	-1.577178E-08	1.000
1120	o279 conc	2.427000E-08	3.945944E-08	-1.518944E-08	1.000
1121	o280 conc	2.484250E-08	3.851874E-08	-1.367624E-08	1.000
1122	o281 conc	2.641960E-08	3.762314E-08	-1.120354E-08	1.000
1123	o282 conc	2.847000E-08	3.676975E-08	-8.299752E-09	1.000
1124	o283 conc	3.041950E-08	3.595576E-08	-5.536257E-09	1.000
1125	o284 conc	3.169390E-08	3.517842E-08	-3.484517E-09	1.000
1126	o285 conc	3.171880E-08	3.443509E-08	-2.716291E-09	1.000
1127	o286 conc	3.013650E-08	3.372324E-08	-3.586742E-09	1.000
1128	o287 conc	2.751750E-08	3.304045E-08	-5.522948E-09	1.000
1129	o288 conc	2.468130E-08	3.238441E-08	-7.703109E-09	1.000
1130	o289 conc	2.244710E-08	3.175295E-08	-9.305850E-09	1.000
1131	o290 conc	2.163440E-08	3.114402E-08	-9.509624E-09	1.000
1132	o291 conc	2.300960E-08	3.055572E-08	-7.546116E-09	1.000
1133	o292 conc	2.652400E-08	2.998624E-08	-3.462243E-09	1.000
1134	o293 conc	3.148730E-08	2.943395E-08	2.053346E-09	1.000
1135	o294 conc	3.719240E-08	2.889732E-08	8.295076E-09	1.000
1136	o295 conc	4.293170E-08	2.837496E-08	1.455674E-08	1.000
1137	o296 conc	4.799810E-08	2.786559E-08	2.013251E-08	1.000
1138	o297 conc	5.168400E-08	2.736806E-08	2.431594E-08	1.000
1139	o298 conc	5.328230E-08	2.688133E-08	2.640097E-08	1.000
1140	o299 conc	5.226800E-08	2.640447E-08	2.586353E-08	1.000
1141	o300 conc	4.918680E-08	2.593667E-08	2.325013E-08	1.000
1142	o301 conc	4.497330E-08	2.547719E-08	1.949611E-08	1.000
1143	o302 conc	4.056260E-08	2.502540E-08	1.553720E-08	1.000
1144	o303 conc	3.689010E-08	2.458076E-08	1.230934E-08	1.000
1145	o304 conc	3.486190E-08	2.414280E-08	1.071910E-08	1.000
1146	o305 conc	3.468440E-08	2.371112E-08	1.097328E-08	1.000
1147	o306 conc	3.584280E-08	2.328540E-08	1.255740E-08	1.000
1148	o307 conc	3.778920E-08	2.286537E-08	1.492383E-08	1.000
1149	o308 conc	3.997600E-08	2.245083E-08	1.752517E-08	1.000
1150	o309 conc	4.185520E-08	2.204161E-08	1.981359E-08	1.000
1151	o310	4.287920E-08	2.163760E-08	2.124160E-08	1.000

		conc				
1152	o311	conc	4.250540E-08	2.123872E-08	2.126668E-08	1.000
1153	o312	conc	4.056860E-08	2.084492E-08	1.972368E-08	1.000
1154	o313	conc	3.752820E-08	2.045620E-08	1.707200E-08	1.000
1155	o314	conc	3.390200E-08	2.007256E-08	1.382944E-08	1.000
1156	o315	conc	3.020810E-08	1.969404E-08	1.051406E-08	1.000
1157	o316	conc	2.696450E-08	1.932066E-08	7.643837E-09	1.000
1158	o317	conc	2.468900E-08	1.895251E-08	5.736494E-09	1.000
1159	o318	conc	2.389950E-08	1.858963E-08	5.309871E-09	1.000
1160	o319	conc	2.486570E-08	1.823211E-08	6.633593E-09	1.000
1161	o320	conc	2.718140E-08	1.788002E-08	9.301384E-09	1.000
1162	o321	conc	3.032590E-08	1.753343E-08	1.279247E-08	1.000
1163	o322	conc	3.377870E-08	1.719244E-08	1.658626E-08	1.000
1164	o323	conc	3.701890E-08	1.685711E-08	2.016179E-08	1.000
1165	o324	conc	3.952600E-08	1.652750E-08	2.299850E-08	1.000
1166	o325	conc	4.077930E-08	1.620369E-08	2.457561E-08	1.000
1167	o326	conc	4.038680E-08	1.588573E-08	2.450107E-08	1.000
1168	o327	conc	3.855690E-08	1.557367E-08	2.298323E-08	1.000
1169	o328	conc	3.567240E-08	1.526755E-08	2.040485E-08	1.000
1170	o329	conc	3.211630E-08	1.496739E-08	1.714891E-08	1.000
1171	o330	conc	2.827140E-08	1.467323E-08	1.359817E-08	1.000
1172	o331	conc	2.452070E-08	1.438507E-08	1.013563E-08	1.000
1173	o332	conc	2.124710E-08	1.410291E-08	7.144189E-09	1.000
1174	o333	conc	1.879250E-08	1.382675E-08	4.965754E-09	1.000
1175	o334	conc	1.716460E-08	1.355656E-08	3.608044E-09	1.000
1176	o335	conc	1.620760E-08	1.329231E-08	2.915288E-09	1.000
1177	o336	conc	1.576470E-08	1.303398E-08	2.730723E-09	1.000
1178	o337	conc	1.567900E-08	1.278150E-08	2.897496E-09	1.000
1179	o338	conc	1.579370E-08	1.253484E-08	3.258862E-09	1.000
1180	o339	conc	1.595200E-08	1.229391E-08	3.658085E-09	1.000
1181	o340	conc	1.600030E-08	1.205866E-08	3.941636E-09	1.000
1182	o341	conc	1.587080E-08	1.182901E-08	4.041792E-09	1.000
1183	o342	conc	1.558860E-08	1.160486E-08	3.983738E-09	1.000
1184	o343	conc	1.518340E-08	1.138614E-08	3.797264E-09	1.000
1185	o344	conc	1.468500E-08	1.117274E-08	3.512265E-09	1.000
1186	o345	conc	1.412300E-08	1.096456E-08	3.158441E-09	1.000
1187	o346	conc	1.352730E-08	1.076150E-08	2.765796E-09	1.000

1188	o347	conc	1.292740E-08	1.056346E-08	2.363937E-09	1.000
1189	o348	conc	1.234810E-08	1.037032E-08	1.977776E-09	1.000
1190	o349	conc	1.180590E-08	1.018198E-08	1.623925E-09	1.000
1191	o350	conc	1.131620E-08	9.998302E-09	1.317898E-09	1.000
1192	o351	conc	1.089450E-08	9.819187E-09	1.075313E-09	1.000
1193	o352	conc	1.055630E-08	9.644515E-09	9.117851E-10	1.000
1194	o353	conc	1.031710E-08	9.474167E-09	8.429331E-10	1.000
1195	o354	conc	1.019230E-08	9.308026E-09	8.842742E-10	1.000
1196	o355	conc	1.018390E-08	9.145974E-09	1.037926E-09	1.000
1197	o356	conc	1.025410E-08	8.987895E-09	1.266205E-09	1.000
1198	o357	conc	1.035830E-08	8.833674E-09	1.524626E-09	1.000
1199	o358	conc	1.045180E-08	8.683195E-09	1.768605E-09	1.000
1200	o359	conc	1.048990E-08	8.536346E-09	1.953554E-09	1.000
1201	o360	conc	1.042780E-08	8.393016E-09	2.034784E-09	1.000
1202	o361	conc	1.022100E-08	8.253095E-09	1.967905E-09	1.000
1203	o362	conc	9.846240E-09	8.116478E-09	1.729762E-09	1.000
1204	o363	conc	9.387050E-09	7.983058E-09	1.403992E-09	1.000
1205	o364	conc	8.959660E-09	7.852735E-09	1.106925E-09	1.000
1206	o365	conc	8.680350E-09	7.725408E-09	9.549417E-10	1.000
1207	o366	conc	8.665390E-09	7.600981E-09	1.064409E-09	1.000
1208	o367	conc	9.031040E-09	7.479360E-09	1.551680E-09	1.000
1209	o368	conc	9.893570E-09	7.360453E-09	2.533117E-09	1.000
1210	o369	conc	1.134860E-08	7.244172E-09	4.104428E-09	1.000
1211	o370	conc	1.331210E-08	7.130430E-09	6.181670E-09	1.000
1212	o371	conc	1.560750E-08	7.019147E-09	8.588353E-09	1.000
1213	o372	conc	1.805770E-08	6.910240E-09	1.114746E-08	1.000
1214	o373	conc	2.048540E-08	6.803634E-09	1.368177E-08	1.000
1215	o374	conc	2.271350E-08	6.699253E-09	1.601425E-08	1.000
1216	o375	conc	2.456470E-08	6.597027E-09	1.796767E-08	1.000
1217	o376	conc	2.587030E-08	6.496887E-09	1.937341E-08	1.000
1218	o377	conc	2.660560E-08	6.398765E-09	2.020683E-08	1.000
1219	o378	conc	2.686570E-08	6.302599E-09	2.056310E-08	1.000
1220	o379	conc	2.674930E-08	6.208328E-09	2.054097E-08	1.000
1221	o380	conc	2.635510E-08	6.115893E-09	2.023921E-08	1.000
1222	o381	conc	2.578180E-08	6.025237E-09	1.975656E-08	1.000
1223	o382	conc	2.512810E-08	5.936307E-09	1.919179E-08	1.000
1224	o383		2.448710E-08	5.849051E-09	1.863805E-08	1.000

conc

1225	o384 conc	2.393560E-08	5.763419E-09	1.817218E-08	1.000
1226	o385 conc	2.354760E-08	5.679365E-09	1.786824E-08	1.000
1227	o386 conc	2.339700E-08	5.596842E-09	1.780016E-08	1.000
1228	o387 conc	2.355770E-08	5.515807E-09	1.804189E-08	1.000
1229	o388 conc	2.410370E-08	5.436218E-09	1.866748E-08	1.000
1230	o389 conc	2.510890E-08	5.358036E-09	1.975086E-08	1.000
1231	o390 conc	2.660560E-08	5.281222E-09	2.132438E-08	1.000
1232	o391 conc	2.841940E-08	5.205739E-09	2.321366E-08	1.000
1233	o392 conc	3.031210E-08	5.131553E-09	2.518055E-08	1.000
1234	o393 conc	3.204560E-08	5.058629E-09	2.698697E-08	1.000
1235	o394 conc	3.338170E-08	4.986935E-09	2.839476E-08	1.000
1236	o395 conc	3.408220E-08	4.916441E-09	2.916576E-08	1.000
1237	o396 conc	3.390880E-08	4.847117E-09	2.906168E-08	1.000
1238	o397 conc	3.266310E-08	4.778934E-09	2.788417E-08	1.000
1239	o398 conc	3.049240E-08	4.711865E-09	2.578054E-08	1.000
1240	o399 conc	2.772300E-08	4.645883E-09	2.307712E-08	1.000
1241	o400 conc	2.468260E-08	4.580964E-09	2.010164E-08	1.000
1242	o401 conc	2.169880E-08	4.517084E-09	1.718172E-08	1.000
1243	o402 conc	1.909940E-08	4.454218E-09	1.464518E-08	1.000
1244	o403 conc	1.721200E-08	4.392344E-09	1.281966E-08	1.000
1245	o404 conc	1.634920E-08	4.331442E-09	1.201776E-08	1.000
1246	o405 conc	1.656890E-08	4.271489E-09	1.229741E-08	1.000
1247	o406 conc	1.771620E-08	4.212466E-09	1.350373E-08	1.000
1248	o407 conc	1.962940E-08	4.154354E-09	1.547505E-08	1.000
1249	o408 conc	2.214720E-08	4.097133E-09	1.805007E-08	1.000
1250	o409 conc	2.510800E-08	4.040787E-09	2.106721E-08	1.000
1251	o410 conc	2.835030E-08	3.985296E-09	2.436500E-08	1.000
1252	o411 conc	3.171240E-08	3.930645E-09	2.778176E-08	1.000
1253	o412 conc	3.502540E-08	3.876816E-09	3.114858E-08	1.000
1254	o413 conc	3.811070E-08	3.823795E-09	3.428691E-08	1.000
1255	o414 conc	4.078870E-08	3.771565E-09	3.701713E-08	1.000
1256	o415 conc	4.288030E-08	3.720113E-09	3.916019E-08	1.000
1257	o416 conc	4.420600E-08	3.669423E-09	4.053658E-08	1.000
1258	o417 conc	4.458640E-08	3.619481E-09	4.096692E-08	1.000
1259	o418 conc	4.384330E-08	3.570274E-09	4.027303E-08	1.000
1260	o419 conc	4.198300E-08	3.521788E-09	3.846121E-08	1.000

1261	o420 conc	3.941400E-08	3.474012E-09	3.593999E-08	1.000
1262	o421 conc	3.659730E-08	3.426931E-09	3.317037E-08	1.000
1263	o422 conc	3.399400E-08	3.380534E-09	3.061347E-08	1.000
1264	o423 conc	3.204760E-08	3.334810E-09	2.871279E-08	1.000
1265	o424 conc	3.086660E-08	3.289745E-09	2.757685E-08	1.000
1266	o425 conc	3.025930E-08	3.245330E-09	2.701397E-08	1.000
1267	o426 conc	3.002380E-08	3.201553E-09	2.682225E-08	1.000
1268	o427 conc	2.995800E-08	3.158404E-09	2.679960E-08	1.000
1269	o428 conc	2.985980E-08	3.115871E-09	2.674393E-08	1.000
1270	o429 conc	2.952710E-08	3.073945E-09	2.645315E-08	1.000
1271	o430 conc	2.876140E-08	3.032616E-09	2.572878E-08	1.000
1272	o431 conc	2.753560E-08	2.991873E-09	2.454373E-08	1.000
1273	o432 conc	2.606870E-08	2.951708E-09	2.311699E-08	1.000
1274	o433 conc	2.459860E-08	2.912111E-09	2.168649E-08	1.000
1275	o434 conc	2.336330E-08	2.873072E-09	2.049023E-08	1.000
1276	o435 conc	2.260070E-08	2.834584E-09	1.976612E-08	1.000
1277	o436 conc	2.254880E-08	2.796636E-09	1.975216E-08	1.000
1278	o437 conc	2.344480E-08	2.759221E-09	2.068558E-08	1.000
1279	o438 conc	2.536450E-08	2.722330E-09	2.264217E-08	1.000
1280	o439 conc	2.800460E-08	2.685954E-09	2.531865E-08	1.000
1281	o440 conc	3.100760E-08	2.650087E-09	2.835751E-08	1.000
1282	o441 conc	3.401610E-08	2.614719E-09	3.140138E-08	1.000
1283	o442 conc	3.667270E-08	2.579843E-09	3.409286E-08	1.000
1284	o443 conc	3.862000E-08	2.545452E-09	3.607455E-08	1.000
1285	o444 conc	3.950070E-08	2.511538E-09	3.698916E-08	1.000
1286	o445 conc	3.908620E-08	2.478093E-09	3.660811E-08	1.000
1287	o446 conc	3.766020E-08	2.445111E-09	3.521509E-08	1.000
1288	o447 conc	3.563300E-08	2.412584E-09	3.322042E-08	1.000
1289	o448 conc	3.341490E-08	2.380506E-09	3.103439E-08	1.000
1290	o449 conc	3.141630E-08	2.348869E-09	2.906743E-08	1.000
1291	o450 conc	3.004740E-08	2.317668E-09	2.772973E-08	1.000
1292	o451 conc	2.971880E-08	2.286895E-09	2.743191E-08	1.000
1293	o452 conc	3.075270E-08	2.256544E-09	2.849616E-08	1.000
1294	o453 conc	3.286720E-08	2.226609E-09	3.064059E-08	1.000
1295	o454 conc	3.552650E-08	2.197084E-09	3.332942E-08	1.000
1296	o455 conc	3.819390E-08	2.167962E-09	3.602594E-08	1.000
1297	o456	4.033280E-08	2.139238E-09	3.819356E-08	1.000

						conc
1298	o457	4.140650E-08	2.110905E-09	3.929559E-08	1.000	conc
1299	o458	4.087830E-08	2.082958E-09	3.879534E-08	1.000	conc
1300	o459	3.826280E-08	2.055392E-09	3.620741E-08	1.000	conc
1301	o460	3.372330E-08	2.028200E-09	3.169510E-08	1.000	conc
1302	o461	2.786850E-08	2.001377E-09	2.586712E-08	1.000	conc
1303	o462	2.131620E-08	1.974919E-09	1.934128E-08	1.000	conc
1304	o463	1.468380E-08	1.948818E-09	1.273498E-08	1.000	conc
1305	o464	8.588990E-09	1.923071E-09	6.665919E-09	1.000	conc
1306	o465	3.649360E-09	1.897672E-09	1.751688E-09	1.000	conc
1307	o466	4.677260E-10	1.872616E-09	-1.404890E-09	1.000	conc
1308	o467	0.00000	1.847899E-09	-1.847899E-09	1.000	conc
1309	o468	0.00000	1.823515E-09	-1.823515E-09	1.000	conc
1310	o469	1.626740E-09	1.799459E-09	-1.727189E-10	1.000	conc
1311	o470	4.693200E-09	1.775727E-09	2.917473E-09	1.000	conc
1312	o471	8.644330E-09	1.752314E-09	6.892016E-09	1.000	conc
1313	o472	1.320690E-08	1.729216E-09	1.147768E-08	1.000	conc
1314	o473	1.810750E-08	1.706429E-09	1.640107E-08	1.000	conc
1315	o474	2.306840E-08	1.683947E-09	2.138445E-08	1.000	conc
1316	o475	2.780330E-08	1.661766E-09	2.614153E-08	1.000	conc
1317	o476	3.202510E-08	1.639883E-09	3.038522E-08	1.000	conc
1318	o477	3.544690E-08	1.618292E-09	3.382861E-08	1.000	conc
1319	o478	3.778170E-08	1.596990E-09	3.618471E-08	1.000	conc
1320	o479	3.874230E-08	1.575974E-09	3.716633E-08	1.000	conc
1321	o480	3.804200E-08	1.555237E-09	3.648676E-08	1.000	conc
1322	o481	3.556310E-08	1.534778E-09	3.402832E-08	1.000	conc
1323	o482	3.168540E-08	1.514592E-09	3.017081E-08	1.000	conc
1324	o483	2.688000E-08	1.494675E-09	2.538533E-08	1.000	conc
1325	o484	2.161760E-08	1.475023E-09	2.014258E-08	1.000	conc
1326	o485	1.636920E-08	1.455633E-09	1.491357E-08	1.000	conc
1327	o486	1.160560E-08	1.436500E-09	1.016910E-08	1.000	conc
1328	o487	7.797780E-09	1.417623E-09	6.380157E-09	1.000	conc
1329	o488	5.321320E-09	1.398996E-09	3.922324E-09	1.000	conc
1330	o489	4.072160E-09	1.380617E-09	2.691543E-09	1.000	conc
1331	o490	3.795720E-09	1.362481E-09	2.433239E-09	1.000	conc
1332	o491	4.237380E-09	1.344587E-09	2.892793E-09	1.000	conc
1333	o492	5.142490E-09	1.326929E-09	3.815561E-09	1.000	conc

```
o493                 6.256440E-09   1.309506E-09   4.946934E-09    1.000
conc
o494                 7.324600E-09   1.292314E-09   6.032286E-09    1.000
conc
o495                 8.113290E-09   1.275349E-09   6.837941E-09    1.000
conc
o496                 8.575080E-09   1.258609E-09   7.316471E-09    1.000
conc
o497                 8.760030E-09   1.242090E-09   7.517940E-09    1.000
conc
o498                 8.719080E-09   1.225790E-09   7.493290E-09    1.000
conc
o499                 8.503150E-09   1.209705E-09   7.293445E-09    1.000
conc
o500                 8.163170E-09   1.193833E-09   6.969337E-09    1.000
conc

See file mfit.res for more details of residuals in graph-ready format.

See file mfit.seo for composite observation sensitivities.


Objective function ----->

  Sum of squared weighted residuals (ie phi)                =  1.9587E-13


Correlation Coefficient ----->

  Correlation coefficient                                   =  0.98041


Analysis of residuals ----->

  All residuals:-
     Number of residuals with non-zero weight                       =   447
     Mean value of non-zero weighted residuals                      =  1.2032E-08
     Maximum weighted residual [observation "o193"]                 =  6.2256E-08
     Minimum weighted residual [observation "o202"]                 = -4.1444E-08
     Standard variance of weighted residuals                        =  4.5029E-16
     Standard error of weighted residuals                           =  2.1220E-08

     Note: the above variance was obtained by dividing the objective
     function by the number of system degrees of freedom (ie. number of
     observations with non-zero weight plus number of prior information
     articles with non-zero weight minus the number of adjustable parameters.)
     If the degrees of freedom is negative the divisor becomes
     the number of observations with non-zero weight plus the number of
     prior information items with non-zero weight.


Covariance and other statistical matrices cannot be determined:-
Memory conservation is operative so that covariance matrix is not calculated.
```
