# Supplementary material for: Comparing the Fate and Transport of MS2 Bacteriophage and Sodium Fluorescein in a Karstic Chalk Aquifer
Source: Pathogens. 2024 Feb 13;13(2):168. doi: 10.3390/pathogens13020168 (PMC10891704; doi:10.3390/pathogens13020168)
Supplement: Supplementary file 1 [file pathogens-13-00168-s001.zip › ABH2_phage_mfit.pdf]

PEST RUN RECORD: CASE mfit

PEST Version: 16.1

PEST run mode:-

Parameter estimation mode

Case dimensions:-

Number of parameters	:	12
Number of adjustable parameters	:	12
Number of parameter groups	:	1
Number of observations	:	500
Number of prior estimates	:	0

Model command line(s):-

MDP_2RNE

Jacobian command line:-

MDP_2RNE /d
Jacobian read from file Deriv.txt
This is an ASCII file.

Model interface files:-

Templates:
MFIT.tpl
for model input files:
Input.txt

(Parameter values written using single precision protocol.)
(Decimal point always included.)

Instruction files:
MFIT.ins
for reading model output files:
Output.txt

PEST-to-model message file:-

na

Singular value decomposition:-

Perform SVD on XtQX or Q^(1/2)X	:	Q^(1/2)X
Max. number of singular values to employ	:	12
Ratio of lowest/highest singular value	:	5.000000E-07
Record eigenvectors in SVD file	:	yes

Derivatives calculation:-

Param group	Increment type	Increment	Increment low bound	Forward or central	Multiplier (central)	Method (central)
pgnam	relative	1.0000E-02	none	switch	1.500	parabolic

Parameter definitions:-

Name	Trans-formation	Change limit	Initial value	Lower bound	Upper bound

h1	log	factor	80.0000	80.0000	110.000
s1	log	factor	2500.00	1.00000	5000.00
p1	log	factor	0.850000	0.100000	0.999000
o1	log	factor	3.300000E-04	1.000000E-10	1.000000E-02
h2	log	factor	130.000	120.000	140.000
s2	log	factor	750.000	1.00000	5000.00
p2	log	factor	0.780000	0.100000	0.999000
o2	log	factor	1.600000E-05	1.000000E-10	1.000000E-02
h3	log	factor	170.000	160.000	180.000
s3	log	factor	800.000	1.00000	5000.00
p3	log	factor	0.300000	0.100000	0.999000
o3	log	factor	3.500000E-05	1.000000E-10	1.000000E-02

Name	Group	Scale	Offset	Model command number
h1	pgnam	1.00000	0.00000	1
s1	pgnam	1.00000	0.00000	0
p1	pgnam	1.00000	0.00000	1
o1	pgnam	1.00000	0.00000	0
h2	pgnam	1.00000	0.00000	1
s2	pgnam	1.00000	0.00000	0
p2	pgnam	1.00000	0.00000	1
o2	pgnam	1.00000	0.00000	0
h3	pgnam	1.00000	0.00000	1
s3	pgnam	1.00000	0.00000	0
p3	pgnam	1.00000	0.00000	1
o3	pgnam	1.00000	0.00000	0

Prior information:-

No prior information supplied

Observations:-

Observation name	Observation	Weight	Group
o1	1.762070E-39	1.000	conc
o2	2.325900E-39	1.000	conc
o3	0.00000	1.000	conc
o4	0.00000	1.000	conc
o5	3.238130E-38	1.000	conc
o6	0.00000	1.000	conc
o7	0.00000	1.000	conc
o8	1.655480E-37	1.000	conc
o9	4.515590E-37	1.000	conc
o10	0.00000	1.000	conc
o11	0.00000	1.000	conc
o12	5.727360E-36	1.000	conc
o13	3.627010E-37	1.000	conc
o14	0.00000	1.000	conc
o15	2.050830E-35	1.000	conc
o16	9.343950E-35	1.000	conc
o17	0.00000	1.000	conc
o18	0.00000	1.000	conc
o19	9.801770E-34	1.000	conc
o20	4.660000E-34	1.000	conc
o21	0.00000	1.000	conc
o22	1.878980E-33	1.000	conc
o23	1.850280E-32	1.000	conc
o24	0.00000	1.000	conc
o25	0.00000	1.000	conc
o26	1.611970E-31	1.000	conc
o27	1.509350E-31	1.000	conc
o28	0.00000	1.000	conc
o29	4.918830E-33	1.000	conc
o30	3.533770E-30	1.000	conc
o31	0.00000	1.000	conc
o32	0.00000	1.000	conc
o33	2.521790E-29	1.000	conc
o34	3.813090E-29	1.000	conc
o35	0.00000	1.000	conc
o36	0.00000	1.000	conc
o37	0.00000	1.000	conc

o38	0.00000	1.000	conc
o39	0.00000	1.000	conc
o40	2.530210E-28	1.000	conc
o41	9.860960E-28	1.000	conc
o42	0.00000	1.000	conc
o43	0.00000	1.000	conc
o44	9.379650E-27	1.000	conc
o45	4.815690E-27	1.000	conc
o46	0.00000	1.000	conc
o47	1.648990E-26	1.000	conc
o48	1.792110E-25	1.000	conc
o49	0.00000	1.000	conc
o50	0.00000	1.000	conc
o51	1.535060E-24	1.000	conc
o52	1.506930E-24	1.000	conc
o53	0.00000	1.000	conc
o54	0.00000	1.000	conc
o55	3.412410E-23	1.000	conc
o56	0.00000	1.000	conc
o57	0.00000	1.000	conc
o58	2.389050E-22	1.000	conc
o59	3.756800E-22	1.000	conc
o60	0.00000	1.000	conc
o61	0.00000	1.000	conc
o62	6.290950E-21	1.000	conc
o63	0.00000	1.000	conc
o64	0.00000	1.000	conc
o65	3.461990E-20	1.000	conc
o66	8.413140E-20	1.000	conc
o67	0.00000	1.000	conc
o68	0.00000	1.000	conc
o69	1.123570E-18	1.000	conc
o70	0.00000	1.000	conc
o71	0.00000	1.000	conc
o72	4.475000E-18	1.000	conc
o73	1.764230E-17	1.000	conc
o74	0.00000	1.000	conc
o75	0.00000	1.000	conc
o76	1.940590E-16	1.000	conc
o77	7.127540E-17	1.000	conc
o78	0.00000	1.000	conc
o79	4.578440E-16	1.000	conc
o80	3.529640E-15	1.000	conc
o81	0.00000	1.000	conc
o82	0.00000	1.000	conc
o83	5.591460E-15	1.000	conc
o84	5.097810E-14	1.000	conc
o85	0.00000	1.000	conc
o86	0.00000	1.000	conc
o87	4.356530E-13	1.000	conc
o88	4.052020E-13	1.000	conc
o89	0.00000	1.000	conc
o90	2.400000E-14	1.000	conc
o91	9.530250E-12	1.000	conc
o92	0.00000	1.000	conc
o93	0.00000	1.000	conc
o94	6.818530E-11	1.000	conc
o95	1.025520E-10	1.000	conc
o96	0.00000	1.000	conc
o97	0.00000	1.000	conc
o98	1.762580E-09	1.000	conc
o99	0.00000	1.000	conc
o100	0.00000	1.000	conc
o101	9.966190E-09	1.000	conc
o102	2.315860E-08	1.000	conc
o103	0.00000	1.000	conc
o104	0.00000	1.000	conc
o105	3.158230E-07	1.000	conc
o106	0.00000	1.000	conc
o107	0.00000	1.000	conc
o108	1.307490E-06	1.000	conc
o109	4.883490E-06	1.000	conc
o110	0.00000	1.000	conc

o111	0.00000	1.000	conc
o112	5.474160E-05	1.000	conc
o113	1.780180E-05	1.000	conc
o114	0.00000	1.000	conc
o115	1.385330E-04	1.000	conc
o116	9.811610E-04	1.000	conc
o117	0.00000	1.000	conc
o118	0.00000	1.000	conc
o119	9.139440E-03	1.000	conc
o120	6.972520E-03	1.000	conc
o121	0.00000	1.000	conc
o122	0.166320	1.000	conc
o123	0.00000	1.000	conc
o124	0.00000	1.000	conc
o125	0.776139	1.000	conc
o126	2.73776	1.000	conc
o127	0.00000	1.000	conc
o128	0.00000	1.000	conc
o129	31.3458	1.000	conc
o130	8.75085	1.000	conc
o131	0.00000	1.000	conc
o132	86.1408	1.000	conc
o133	963.476	1.000	conc
o134	1747.51	1.000	conc
o135	3591.21	1.000	conc
o136	9918.59	1.000	conc
o137	15848.6	1.000	conc
o138	15510.6	1.000	conc
o139	40579.2	1.000	conc
o140	107955.	1.000	conc
o141	130437.	1.000	conc
o142	87393.5	1.000	conc
o143	113899.	1.000	conc
o144	212482.	1.000	conc
o145	250810.	1.000	conc
o146	238335.	1.000	conc
o147	246662.	1.000	conc
o148	253421.	1.000	conc
o149	228386.	1.000	conc
o150	189898.	1.000	conc
o151	171060.	1.000	conc
o152	209249.	1.000	conc
o153	263462.	1.000	conc
o154	253026.	1.000	conc
o155	213009.	1.000	conc
o156	206588.	1.000	conc
o157	237117.	1.000	conc
o158	281729.	1.000	conc
o159	295557.	1.000	conc
o160	280600.	1.000	conc
o161	280690.	1.000	conc
o162	292713.	1.000	conc
o163	292851.	1.000	conc
o164	279875.	1.000	conc
o165	258901.	1.000	conc
o166	235046.	1.000	conc
o167	213428.	1.000	conc
o168	199164.	1.000	conc
o169	197371.	1.000	conc
o170	203306.	1.000	conc
o171	170565.	1.000	conc
o172	94276.1	1.000	conc
o173	82388.0	1.000	conc
o174	144291.	1.000	conc
o175	161528.	1.000	conc
o176	128019.	1.000	conc
o177	108437.	1.000	conc
o178	110262.	1.000	conc
o179	121310.	1.000	conc
o180	133760.	1.000	conc
o181	139392.	1.000	conc
o182	129247.	1.000	conc
o183	116109.	1.000	conc

o184	126217.	1.000	conc
o185	139498.	1.000	conc
o186	126431.	1.000	conc
o187	110561.	1.000	conc
o188	110899.	1.000	conc
o189	109252.	1.000	conc
o190	99126.8	1.000	conc
o191	92048.0	1.000	conc
o192	89688.8	1.000	conc
o193	88557.5	1.000	conc
o194	94958.0	1.000	conc
o195	107079.	1.000	conc
o196	91369.6	1.000	conc
o197	46576.9	1.000	conc
o198	25748.9	1.000	conc
o199	33799.4	1.000	conc
o200	35631.0	1.000	conc
o201	28383.7	1.000	conc
o202	23252.8	1.000	conc
o203	26145.9	1.000	conc
o204	36201.1	1.000	conc
o205	41993.2	1.000	conc
o206	42503.4	1.000	conc
o207	49159.4	1.000	conc
o208	59113.1	1.000	conc
o209	53550.1	1.000	conc
o210	42313.1	1.000	conc
o211	49127.3	1.000	conc
o212	59932.4	1.000	conc
o213	57765.5	1.000	conc
o214	59051.1	1.000	conc
o215	71289.7	1.000	conc
o216	78099.1	1.000	conc
o217	74866.7	1.000	conc
o218	69517.8	1.000	conc
o219	70436.8	1.000	conc
o220	82025.2	1.000	conc
o221	92322.6	1.000	conc
o222	93098.2	1.000	conc
o223	93041.5	1.000	conc
o224	100680.	1.000	conc
o225	120120.	1.000	conc
o226	135730.	1.000	conc
o227	127404.	1.000	conc
o228	121217.	1.000	conc
o229	136763.	1.000	conc
o230	129342.	1.000	conc
o231	88468.1	1.000	conc
o232	70850.2	1.000	conc
o233	80996.3	1.000	conc
o234	79578.4	1.000	conc
o235	71476.0	1.000	conc
o236	74543.8	1.000	conc
o237	73858.3	1.000	conc
o238	60598.7	1.000	conc
o239	54071.0	1.000	conc
o240	58191.7	1.000	conc
o241	57334.7	1.000	conc
o242	52277.3	1.000	conc
o243	52213.6	1.000	conc
o244	53457.5	1.000	conc
o245	51425.7	1.000	conc
o246	50764.7	1.000	conc
o247	52332.4	1.000	conc
o248	49494.8	1.000	conc
o249	41204.9	1.000	conc
o250	31483.7	1.000	conc
o251	24486.3	1.000	conc
o252	21590.2	1.000	conc
o253	18193.9	1.000	conc
o254	13790.9	1.000	conc
o255	13243.4	1.000	conc
o256	14937.6	1.000	conc

o257	13632.5	1.000	conc
o258	9520.91	1.000	conc
o259	5061.07	1.000	conc
o260	3493.82	1.000	conc
o261	5447.37	1.000	conc
o262	7290.70	1.000	conc
o263	7251.73	1.000	conc
o264	5666.69	1.000	conc
o265	4002.47	1.000	conc
o266	3705.62	1.000	conc
o267	4079.18	1.000	conc
o268	4342.79	1.000	conc
o269	4657.45	1.000	conc
o270	4709.12	1.000	conc
o271	3662.07	1.000	conc
o272	2533.16	1.000	conc
o273	2971.71	1.000	conc
o274	3480.80	1.000	conc
o275	2837.98	1.000	conc
o276	3585.37	1.000	conc
o277	5772.33	1.000	conc
o278	5009.78	1.000	conc
o279	2281.37	1.000	conc
o280	2563.73	1.000	conc
o281	4521.34	1.000	conc
o282	4986.85	1.000	conc
o283	5110.52	1.000	conc
o284	6018.91	1.000	conc
o285	6769.64	1.000	conc
o286	7514.82	1.000	conc
o287	9606.69	1.000	conc
o288	11461.7	1.000	conc
o289	10743.1	1.000	conc
o290	10720.9	1.000	conc
o291	13733.1	1.000	conc
o292	14813.8	1.000	conc
o293	11710.4	1.000	conc
o294	7841.36	1.000	conc
o295	6105.19	1.000	conc
o296	6112.11	1.000	conc
o297	6769.24	1.000	conc
o298	7018.63	1.000	conc
o299	6603.01	1.000	conc
o300	6064.89	1.000	conc
o301	5981.44	1.000	conc
o302	6769.91	1.000	conc
o303	8101.42	1.000	conc
o304	9429.78	1.000	conc
o305	10216.6	1.000	conc
o306	10232.1	1.000	conc
o307	9650.45	1.000	conc
o308	8673.18	1.000	conc
o309	7502.49	1.000	conc
o310	6345.57	1.000	conc
o311	5411.55	1.000	conc
o312	4907.69	1.000	conc
o313	4888.55	1.000	conc
o314	5145.72	1.000	conc
o315	5444.77	1.000	conc
o316	5566.72	1.000	conc
o317	5430.42	1.000	conc
o318	5027.17	1.000	conc
o319	4349.18	1.000	conc
o320	3446.13	1.000	conc
o321	2499.34	1.000	conc
o322	1708.47	1.000	conc
o323	1259.21	1.000	conc
o324	1157.75	1.000	conc
o325	1285.64	1.000	conc
o326	1521.98	1.000	conc
o327	1760.85	1.000	conc
o328	1942.61	1.000	conc
o329	2016.51	1.000	conc

o330	1934.51	1.000	conc
o331	1702.19	1.000	conc
o332	1373.90	1.000	conc
o333	1005.74	1.000	conc
o334	649.533	1.000	conc
o335	339.055	1.000	conc
o336	103.355	1.000	conc
o337	0.00000	1.000	conc
o338	0.00000	1.000	conc
o339	0.00000	1.000	conc
o340	0.00000	1.000	conc
o341	0.00000	1.000	conc
o342	0.00000	1.000	conc
o343	0.00000	1.000	conc
o344	24.6039	1.000	conc
o345	189.070	1.000	conc
o346	371.669	1.000	conc
o347	489.454	1.000	conc
o348	467.298	1.000	conc
o349	323.288	1.000	conc
o350	136.783	1.000	conc
o351	0.00000	1.000	conc
o352	0.00000	1.000	conc
o353	0.00000	1.000	conc
o354	0.00000	1.000	conc
o355	15.9872	1.000	conc
o356	27.6263	1.000	conc
o357	20.8621	1.000	conc
o358	0.00000	1.000	conc
o359	0.00000	1.000	conc
o360	0.00000	1.000	conc
o361	0.00000	1.000	conc
o362	38.6360	1.000	conc
o363	161.098	1.000	conc
o364	316.812	1.000	conc
o365	483.800	1.000	conc
o366	642.739	1.000	conc
o367	788.596	1.000	conc
o368	921.125	1.000	conc
o369	1039.77	1.000	conc
o370	1127.28	1.000	conc
o371	1141.63	1.000	conc
o372	1038.82	1.000	conc
o373	787.834	1.000	conc
o374	454.309	1.000	conc
o375	147.371	1.000	conc
o376	0.00000	1.000	conc
o377	0.00000	1.000	conc
o378	178.614	1.000	conc
o379	464.396	1.000	conc
o380	806.740	1.000	conc
o381	1155.84	1.000	conc
o382	1461.89	1.000	conc
o383	1676.50	1.000	conc
o384	1760.11	1.000	conc
o385	1676.55	1.000	conc
o386	1390.50	1.000	conc
o387	931.442	1.000	conc
o388	437.972	1.000	conc
o389	59.1618	1.000	conc
o390	0.00000	1.000	conc
o391	26.7570	1.000	conc
o392	267.289	1.000	conc
o393	557.398	1.000	conc
o394	817.031	1.000	conc
o395	991.351	1.000	conc
o396	1028.94	1.000	conc
o397	885.073	1.000	conc
o398	598.276	1.000	conc
o399	263.623	1.000	conc
o400	0.00000	1.000	conc
o401	0.00000	1.000	conc
o402	0.00000	1.000	conc

o403	0.00000	1.000	conc
o404	152.637	1.000	conc
o405	486.728	1.000	conc
o406	803.196	1.000	conc
o407	999.271	1.000	conc
o408	1001.11	1.000	conc
o409	853.094	1.000	conc
o410	629.831	1.000	conc
o411	404.922	1.000	conc
o412	221.358	1.000	conc
o413	86.4305	1.000	conc
o414	5.41412	1.000	conc
o415	0.00000	1.000	conc
o416	0.00000	1.000	conc
o417	1.83637	1.000	conc
o418	0.00000	1.000	conc
o419	20.0022	1.000	conc
o420	167.357	1.000	conc
o421	356.022	1.000	conc
o422	487.104	1.000	conc
o423	472.761	1.000	conc
o424	331.322	1.000	conc
o425	140.303	1.000	conc
o426	0.00000	1.000	conc
o427	0.00000	1.000	conc
o428	0.00000	1.000	conc
o429	0.00000	1.000	conc
o430	77.2770	1.000	conc
o431	224.980	1.000	conc
o432	377.267	1.000	conc
o433	507.381	1.000	conc
o434	591.128	1.000	conc
o435	612.737	1.000	conc
o436	558.166	1.000	conc
o437	415.570	1.000	conc
o438	220.447	1.000	conc
o439	53.8837	1.000	conc
o440	0.00000	1.000	conc
o441	108.455	1.000	conc
o442	303.139	1.000	conc
o443	466.067	1.000	conc
o444	483.141	1.000	conc
o445	338.699	1.000	conc
o446	141.219	1.000	conc
o447	7.10519	1.000	conc
o448	31.8670	1.000	conc
o449	183.043	1.000	conc
o450	379.758	1.000	conc
o451	541.471	1.000	conc
o452	618.586	1.000	conc
o453	612.838	1.000	conc
o454	530.791	1.000	conc
o455	382.484	1.000	conc
o456	207.541	1.000	conc
o457	60.5508	1.000	conc
o458	0.00000	1.000	conc
o459	46.1642	1.000	conc
o460	192.871	1.000	conc
o461	411.737	1.000	conc
o462	675.194	1.000	conc
o463	918.134	1.000	conc
o464	1050.34	1.000	conc
o465	981.197	1.000	conc
o466	681.595	1.000	conc
o467	304.360	1.000	conc
o468	35.7788	1.000	conc
o469	51.8214	1.000	conc
o470	338.819	1.000	conc
o471	716.697	1.000	conc
o472	999.732	1.000	conc
o473	1047.59	1.000	conc
o474	902.303	1.000	conc
o475	651.723	1.000	conc

```
 o476                    382.911          1.000       conc
 o477                    159.502          1.000       conc
 o478                    18.2148          1.000       conc
 o479                    0.00000          1.000       conc
 o480                    107.393          1.000       conc
 o481                    291.838          1.000       conc
 o482                    452.788          1.000       conc
 o483                    496.542          1.000       conc
 o484                    393.985          1.000       conc
 o485                    213.383          1.000       conc
 o486                    31.0702          1.000       conc
 o487                    0.00000          1.000       conc
 o488                    0.00000          1.000       conc
 o489                    0.00000          1.000       conc
 o490                    30.4946          1.000       conc
 o491                    180.699          1.000       conc
 o492                    340.231          1.000       conc
 o493                    471.098          1.000       conc
 o494                    538.884          1.000       conc
 o495                    547.549          1.000       conc
 o496                    524.384          1.000       conc
 o497                    496.995          1.000       conc
 o498                    484.919          1.000       conc
 o499                    486.180          1.000       conc
 o500                    495.254          1.000       conc


Control settings:-

   Initial lambda                                                  :  10.000
   Lambda adjustment factor                                        :  2.0000
   Sufficient new/old phi ratio per optimisation iteration         : 0.30000
   Limiting relative phi reduction between lambdas                 : 1.00000E-02
   Maximum trial lambdas per iteration                             :   8
   Forgive model run failure during lamda testing                  : yes
   Forgive model run failure during Jacobian runs                  : yes

   Perform Broyden's update of Jacobian matrix                     : no
   Undertake observation re-referencing                            : no

   Maximum  factor  parameter change (factor-limited changes)      :  10.000
   Maximum relative parameter change (relative-limited changes)    : na
   Fraction of initial parameter values used in computing
   change limit for near-zero parameters                           : 1.00000E-03
   Allow bending of parameter upgrade vector                       : no
   Allow parameters to stick to their bounds                       : no

   Relative phi reduction below which to begin use of
   central derivatives                                             : 0.10000
   Iteration at which to first consider derivatives switch         :   1

   Relative phi reduction indicating convergence                   : 0.50000E-02
   Number of phi values required within this range                 :   4
   Maximum number of consecutive failures to lower phi             :   4
   Minimal relative parameter change indicating convergence        : 0.50000E-02
   Number of consecutive iterations with minimal param change      :   4
   Maximum number of optimisation iterations                       : 200

   Attempt automatic user intervention                             : no

   Attempt reuse of parameter sensitivities                        : no

   Scale parameters by their bounds                                : yes


File saving options: -

   Save best JCO file                                              : yes
   Save multiple JCO files                                         : no
   Save multiple REI files                                         : no
   Save multiple PAR files                                         : yes
```

```
                              OPTIMISATION RECORD


INITIAL CONDITIONS:
   Sum of squared weighted residuals (ie phi) =  1.02166E+11

      Current parameter values
      h1                80.0000
      s1                2500.00
      p1               0.850000
      o1               3.300000E-04
      h2                130.000
      s2                750.000
      p2               0.780000
      o2               1.600000E-05
      h3                170.000
      s3                800.000
      p3               0.300000
      o3               3.500000E-05


OPTIMISATION ITERATION NO.        : 1
   Model calls so far             : 1
   Derivative model calls so far  : 0
   Starting phi for this iteration:  1.02166E+11

       Lambda =   10.000     ----->
          Phi =  8.52511E+10  (  0.834 of starting phi)

       Lambda =   5.0000     ----->
          Phi =  8.52511E+10  (  0.834 of starting phi)

   No more lambdas: relative phi reduction between lambdas less than 0.0100
   Lowest phi this iteration:  8.52511E+10

      Current parameter values                 Previous parameter values
      h1                80.5239                h1                80.0000
      s1                5000.00                s1                2500.00
      p1               0.852430                p1               0.850000
      o1               3.556670E-04            o1               3.300000E-04
      h2                129.535                h2                130.000
      s2                710.842                s2                750.000
      p2               0.800274                p2               0.780000
      o2               1.532551E-05            o2               1.600000E-05
      h3                167.684                h3                170.000
      s3                782.079                s3                800.000
      p3               0.299536                p3               0.300000
      o3               3.762602E-05            o3               3.500000E-05
   Maximum   factor change:  2.000     ["s1"]
   Maximum relative change:  1.000     ["s1"]


OPTIMISATION ITERATION NO.        : 2
   Model calls so far             : 9
   Derivative model calls so far  : 1
   Starting phi for this iteration:  8.52511E+10
       param "s1" frozen: gradient and update vectors out of bounds

       Lambda =   5.0000     ----->
          Phi =  8.46248E+10  (  0.993 of starting phi)

       Lambda =   2.5000     ----->
          Phi =  8.46248E+10  (  0.993 of starting phi)

   No more lambdas: relative phi reduction between lambdas less than 0.0100
   Lowest phi this iteration:  8.46248E+10
   Relative phi reduction between optimisation iterations less than 0.1000
   Switch to higher order derivatives calculation

      Current parameter values                 Previous parameter values
      h1                80.8050                h1                80.5239
      s1                5000.00                s1                5000.00
      p1               0.855389                p1               0.852430
```

```
        o1              3.357590E-04            o1                3.556670E-04
        h2               129.564                h2                 129.535
        s2               737.552                s2                 710.842
        p2              0.777974                p2                0.800274
        o2              1.624590E-05            o2                1.532551E-05
        h3               167.733                h3                 167.684
        s3               806.636                s3                 782.079
        p3              0.299353                p3                0.299536
        o3              3.474980E-05            o3                3.762602E-05
     Maximum   factor change:  1.083     ["o3"]
     Maximum relative change: 7.6442E-02 ["o3"]


  OPTIMISATION ITERATION NO.        : 3
     Model calls so far             : 17
     Derivative model calls so far  : 2
     Starting phi for this iteration:  8.46248E+10
     All frozen parameters freed.

         Lambda =   2.5000     ----->
            Phi =  8.46678E+10  (  1.001 times starting phi)

         Lambda =   1.2500     ----->
            Phi =  8.46678E+10  (  1.001 times starting phi)

     No more lambdas: relative phi reduction between lambdas less than 0.0100
     Lowest phi this iteration:  8.46678E+10

        Current parameter values                Previous parameter values
        h1               80.9645                h1                 80.8050
        s1               4675.20                s1                 5000.00
        p1              0.856556                p1                0.855389
        o1              3.258279E-04            o1                3.357590E-04
        h2               129.573                h2                 129.564
        s2               743.722                s2                 737.552
        p2              0.764592                p2                0.777974
        o2              1.670360E-05            o2                1.624590E-05
        h3               167.726                h3                 167.733
        s3               805.880                s3                 806.636
        p3              0.299232                p3                0.299353
        o3              3.459782E-05            o3                3.474980E-05
     Maximum   factor change:  1.069     ["s1"]
     Maximum relative change: 6.4960E-02 ["s1"]


  OPTIMISATION ITERATION NO.        : 4
     Model calls so far             : 31
     Derivative model calls so far  : 3
     Starting phi for this iteration:  8.46678E+10

         Lambda =   1.2500     ----->
            Phi =  8.46335E+10  (  1.000 of starting phi)

         Lambda =  0.62500     ----->
            Phi =  8.46335E+10  (  1.000 of starting phi)

     No more lambdas: relative phi reduction between lambdas less than 0.0100
     Lowest phi this iteration:  8.46335E+10

        Current parameter values                Previous parameter values
        h1               80.9315                h1                 80.9645
        s1               4834.87                s1                 4675.20
        p1              0.856226                p1                0.856556
        o1              3.276094E-04            o1                3.258279E-04
        h2               129.570                h2                 129.573
        s2               743.251                s2                 743.722
        p2              0.764466                p2                0.764592
        o2              1.664407E-05            o2                1.670360E-05
        h3               167.726                h3                 167.726
        s3               804.159                s3                 805.880
        p3              0.299229                p3                0.299232
        o3              3.444588E-05            o3                3.459782E-05
     Maximum   factor change:  1.034     ["s1"]
```

```
   Maximum relative change: 3.4153E-02 ["s1"]


OPTIMISATION ITERATION NO.        : 5
   Model calls so far             : 45
   Derivative model calls so far  : 4
   Starting phi for this iteration:  8.46335E+10

       Lambda =  0.62500     ----->
          Phi =  8.46235E+10  (  1.000 of starting phi)

       Lambda =  0.31250     ----->
          Phi =  8.46235E+10  (  1.000 of starting phi)

   No more lambdas: relative phi reduction between lambdas less than 0.0100
   Lowest phi this iteration:  8.46235E+10

      Current parameter values                 Previous parameter values
      h1               80.9145                 h1               80.9315
      s1               4974.34                 s1               4834.87
      p1              0.856013                 p1              0.856226
      o1              3.285186E-04             o1              3.276094E-04
      h2               129.568                 h2               129.570
      s2               743.390                 s2               743.251
      p2              0.761307                 p2              0.764466
      o2              1.663139E-05             o2              1.664407E-05
      h3               167.725                 h3               167.726
      s3               799.611                 s3               804.159
      p3              0.299194                 p3              0.299229
      o3              3.407141E-05             o3              3.444588E-05
   Maximum   factor change:  1.029     ["s1"]
   Maximum relative change: 2.8846E-02 ["s1"]

   Optimisation complete: the  4 lowest phi's are within a relative distance
                          of eachother of 5.000E-03
   Total model calls:     59

   The model has been run one final time using best parameters.
   Thus all model input files contain best parameter values, and model
   output files contain model results based on these parameters.


                            OPTIMISATION RESULTS

Covariance matrix and parameter confidence intervals cannot be determined:-
Memory conservation is operative so that covariance matrix is not calculated.


Parameters ----->

Parameter      Estimated value
 h1              80.9145
 s1              4974.34
 p1             0.856013
 o1             3.285186E-04
 h2              129.568
 s2              743.390
 p2             0.761307
 o2             1.663139E-05
 h3              167.725
 s3              799.611
 p3             0.299194
 o3             3.407141E-05

See file mfit.sen for parameter sensitivities.


Observations ----->

Observation            Measured       Calculated     Residual       Weight     Group
                       value          value
o1                    1.762070E-39    0.00000       1.762070E-39    1.000
conc
```

o2 conc	2.325900E-39	0.00000	2.325900E-39	1.000
o3 conc	0.00000	0.00000	0.00000	1.000
o4 conc	0.00000	0.00000	0.00000	1.000
o5 conc	3.238130E-38	0.00000	3.238130E-38	1.000
o6 conc	0.00000	0.00000	0.00000	1.000
o7 conc	0.00000	0.00000	0.00000	1.000
o8 conc	1.655480E-37	0.00000	1.655480E-37	1.000
o9 conc	4.515590E-37	0.00000	4.515590E-37	1.000
o10 conc	0.00000	0.00000	0.00000	1.000
o11 conc	0.00000	0.00000	0.00000	1.000
o12 conc	5.727360E-36	0.00000	5.727360E-36	1.000
o13 conc	3.627010E-37	0.00000	3.627010E-37	1.000
o14 conc	0.00000	0.00000	0.00000	1.000
o15 conc	2.050830E-35	0.00000	2.050830E-35	1.000
o16 conc	9.343950E-35	0.00000	9.343950E-35	1.000
o17 conc	0.00000	0.00000	0.00000	1.000
o18 conc	0.00000	0.00000	0.00000	1.000
o19 conc	9.801770E-34	0.00000	9.801770E-34	1.000
o20 conc	4.660000E-34	0.00000	4.660000E-34	1.000
o21 conc	0.00000	0.00000	0.00000	1.000
o22 conc	1.878980E-33	0.00000	1.878980E-33	1.000
o23 conc	1.850280E-32	0.00000	1.850280E-32	1.000
o24 conc	0.00000	0.00000	0.00000	1.000
o25 conc	0.00000	0.00000	0.00000	1.000
o26 conc	1.611970E-31	0.00000	1.611970E-31	1.000
o27 conc	1.509350E-31	0.00000	1.509350E-31	1.000
o28 conc	0.00000	0.00000	0.00000	1.000
o29 conc	4.918830E-33	0.00000	4.918830E-33	1.000
o30 conc	3.533770E-30	0.00000	3.533770E-30	1.000
o31 conc	0.00000	0.00000	0.00000	1.000
o32 conc	0.00000	0.00000	0.00000	1.000
o33 conc	2.521790E-29	0.00000	2.521790E-29	1.000
o34 conc	3.813090E-29	0.00000	3.813090E-29	1.000
o35 conc	0.00000	0.00000	0.00000	1.000
o36 conc	0.00000	0.00000	0.00000	1.000
o37 conc	0.00000	0.00000	0.00000	1.000
o38	0.00000	0.00000	0.00000	1.000

	conc				
913	o39 conc	0.00000	6.035659-314	-6.035659-314	1.000
914	o40 conc	2.530210E-28	7.305196-303	2.530210E-28	1.000
915	o41 conc	9.860960E-28	2.504729-292	9.860960E-28	1.000
916	o42 conc	0.00000	2.657874-282	-2.657874-282	1.000
917	o43 conc	0.00000	9.459494-273	-9.459494-273	1.000
918	o44 conc	9.379650E-27	1.214945-263	9.379650E-27	1.000
919	o45 conc	4.815690E-27	6.020295-255	4.815690E-27	1.000
920	o46 conc	0.00000	1.223449-246	-1.223449-246	1.000
921	o47 conc	1.648990E-26	1.078387-238	1.648990E-26	1.000
922	o48 conc	1.792110E-25	4.340129-231	1.792110E-25	1.000
923	o49 conc	0.00000	8.361718-224	-8.361718-224	1.000
924	o50 conc	0.00000	8.054920-217	-8.054920-217	1.000
925	o51 conc	1.535060E-24	4.038750-210	1.535060E-24	1.000
926	o52 conc	1.506930E-24	1.093899-203	1.506930E-24	1.000
927	o53 conc	0.00000	1.656445-197	-1.656445-197	1.000
928	o54 conc	0.00000	1.447710-191	-1.447710-191	1.000
929	o55 conc	3.412410E-23	7.521997-186	3.412410E-23	1.000
930	o56 conc	0.00000	2.388194-180	-2.388194-180	1.000
931	o57 conc	0.00000	4.753374-175	-4.753374-175	1.000
932	o58 conc	2.389050E-22	6.074144-170	2.389050E-22	1.000
933	o59 conc	3.756800E-22	5.095428-165	3.756800E-22	1.000
934	o60 conc	0.00000	2.864933-160	-2.864933-160	1.000
935	o61 conc	0.00000	1.100849-155	-1.100849-155	1.000
936	o62 conc	6.290950E-21	2.943906-151	6.290950E-21	1.000
937	o63 conc	0.00000	5.573274-147	-5.573274-147	1.000
938	o64 conc	0.00000	7.589918-143	-7.589918-143	1.000
939	o65 conc	3.461990E-20	7.547990-139	3.461990E-20	1.000
940	o66 conc	8.413140E-20	5.559419-135	8.413140E-20	1.000
941	o67 conc	0.00000	3.073301-131	-3.073301-131	1.000
942	o68 conc	0.00000	1.291210-127	-1.291210-127	1.000
943	o69 conc	1.123570E-18	4.171870-124	1.123570E-18	1.000
944	o70 conc	0.00000	1.048198-120	-1.048198-120	1.000
945	o71 conc	0.00000	2.069672-117	-2.069672-117	1.000
946	o72 conc	4.475000E-18	3.243555-114	4.475000E-18	1.000
947	o73 conc	1.764230E-17	4.072719-111	1.764230E-17	1.000
948	o74 conc	0.00000	4.133847-108	-4.133847-108	1.000

949	o75 conc	0.00000	3.420508-105	-3.420508-105	1.000
950	o76 conc	1.940590E-16	2.325743-102	1.940590E-16	1.000
951	o77 conc	7.127540E-17	1.309358E-99	7.127540E-17	1.000
952	o78 conc	0.00000	6.147568E-97	-6.147568E-97	1.000
953	o79 conc	4.578440E-16	2.423614E-94	4.578440E-16	1.000
954	o80 conc	3.529640E-15	8.075294E-92	3.529640E-15	1.000
955	o81 conc	0.00000	2.288072E-89	-2.288072E-89	1.000
956	o82 conc	0.00000	5.545611E-87	-5.545611E-87	1.000
957	o83 conc	5.591460E-15	1.156186E-84	5.591460E-15	1.000
958	o84 conc	5.097810E-14	2.084589E-82	5.097810E-14	1.000
959	o85 conc	0.00000	3.266892E-80	-3.266892E-80	1.000
960	o86 conc	0.00000	4.471739E-78	-4.471739E-78	1.000
961	o87 conc	4.356530E-13	5.371000E-76	4.356530E-13	1.000
962	o88 conc	4.052020E-13	5.685782E-74	4.052020E-13	1.000
963	o89 conc	0.00000	5.327397E-72	-5.327397E-72	1.000
964	o90 conc	2.400000E-14	4.435909E-70	2.400000E-14	1.000
965	o91 conc	9.530250E-12	3.295105E-68	9.530250E-12	1.000
966	o92 conc	0.00000	2.191690E-66	-2.191690E-66	1.000
967	o93 conc	0.00000	1.309929E-64	-1.309929E-64	1.000
968	o94 conc	6.818530E-11	7.059044E-63	6.818530E-11	1.000
969	o95 conc	1.025520E-10	3.440988E-61	1.025520E-10	1.000
970	o96 conc	0.00000	1.521986E-59	-1.521986E-59	1.000
971	o97 conc	0.00000	6.126662E-58	-6.126662E-58	1.000
972	o98 conc	1.762580E-09	2.250954E-56	1.762580E-09	1.000
973	o99 conc	0.00000	7.568886E-55	-7.568886E-55	1.000
974	o100 conc	0.00000	2.335426E-53	-2.335426E-53	1.000
975	o101 conc	9.966190E-09	6.629340E-52	9.966190E-09	1.000
976	o102 conc	2.315860E-08	1.735413E-50	2.315860E-08	1.000
977	o103 conc	0.00000	4.199768E-49	-4.199768E-49	1.000
978	o104 conc	0.00000	9.515132E-48	-9.515132E-48	1.000
979	o105 conc	3.158230E-07	3.666833E-46	3.158230E-07	1.000
980	o106 conc	0.00000	2.172860E-43	-2.172860E-43	1.000
981	o107 conc	0.00000	1.991626E-40	-1.991626E-40	1.000
982	o108 conc	1.307490E-06	1.395963E-37	1.307490E-06	1.000
983	o109 conc	4.883490E-06	7.416672E-35	4.883490E-06	1.000
984	o110 conc	0.00000	3.008470E-32	-3.008470E-32	1.000
985	o111	0.00000	9.385026E-30	-9.385026E-30	1.000

conc

o112	5.474160E-05	2.267373E-27	5.474160E-05	1.000	conc
o113	1.780180E-05	4.271168E-25	1.780180E-05	1.000	conc
o114	0.00000	6.314578E-23	-6.314578E-23	1.000	conc
o115	1.385330E-04	7.373218E-21	1.385330E-04	1.000	conc
o116	9.811610E-04	6.841230E-19	9.811610E-04	1.000	conc
o117	0.00000	5.073846E-17	-5.073846E-17	1.000	conc
o118	0.00000	3.025131E-15	-3.025131E-15	1.000	conc
o119	9.139440E-03	1.457985E-13	9.139440E-03	1.000	conc
o120	6.972520E-03	5.710664E-12	6.972520E-03	1.000	conc
o121	0.00000	1.827237E-10	-1.827237E-10	1.000	conc
o122	0.166320	4.800216E-09	0.166320	1.000	conc
o123	0.00000	1.040404E-07	-1.040404E-07	1.000	conc
o124	0.00000	1.869291E-06	-1.869291E-06	1.000	conc
o125	0.776139	2.796963E-05	0.776111	1.000	conc
o126	2.73776	3.500918E-04	2.73741	1.000	conc
o127	0.00000	3.681848E-03	-3.681848E-03	1.000	conc
o128	0.00000	3.267388E-02	-3.267388E-02	1.000	conc
o129	31.3458	0.245705	31.1001	1.000	conc
o130	8.75085	1.57220	7.17865	1.000	conc
o131	0.00000	8.59533	-8.59533	1.000	conc
o132	86.1408	40.3144	45.8264	1.000	conc
o133	963.476	162.888	800.588	1.000	conc
o134	1747.51	569.344	1178.17	1.000	conc
o135	3591.21	1729.02	1862.19	1.000	conc
o136	9918.59	4583.00	5335.59	1.000	conc
o137	15848.6	10655.4	5193.20	1.000	conc
o138	15510.6	21849.1	-6338.54	1.000	conc
o139	40579.2	39760.4	818.777	1.000	conc
o140	107955.	64683.6	43271.4	1.000	conc
o141	130437.	94899.5	35537.5	1.000	conc
o142	87393.5	126898.	-39504.6	1.000	conc
o143	113899.	156630.	-42731.4	1.000	conc
o144	212482.	181097.	31384.9	1.000	conc
o145	250810.	199296.	51513.7	1.000	conc
o146	238335.	212057.	26277.8	1.000	conc
o147	246662.	221096.	25566.5	1.000	conc

1022	o148 conc	253421.	228026.	25395.4	1.000
1023	o149 conc	228386.	233845.	-5458.91	1.000
1024	o150 conc	189898.	238929.	-49031.5	1.000
1025	o151 conc	171060.	243285.	-72225.3	1.000
1026	o152 conc	209249.	246799.	-37550.3	1.000
1027	o153 conc	263462.	249380.	14082.2	1.000
1028	o154 conc	253026.	250996.	2030.04	1.000
1029	o155 conc	213009.	251668.	-38658.8	1.000
1030	o156 conc	206588.	251445.	-44857.2	1.000
1031	o157 conc	237117.	250392.	-13274.6	1.000
1032	o158 conc	281729.	248575.	33154.0	1.000
1033	o159 conc	295557.	246064.	49493.0	1.000
1034	o160 conc	280600.	242926.	37673.9	1.000
1035	o161 conc	280690.	239226.	41463.7	1.000
1036	o162 conc	292713.	235028.	57685.3	1.000
1037	o163 conc	292851.	230390.	62460.9	1.000
1038	o164 conc	279875.	225371.	54504.2	1.000
1039	o165 conc	258901.	220024.	38877.4	1.000
1040	o166 conc	235046.	214400.	20646.4	1.000
1041	o167 conc	213428.	208546.	4881.77	1.000
1042	o168 conc	199164.	202508.	-3344.04	1.000
1043	o169 conc	197371.	196326.	1044.77	1.000
1044	o170 conc	203306.	190039.	13267.1	1.000
1045	o171 conc	170565.	183681.	-13116.1	1.000
1046	o172 conc	94276.1	177285.	-83008.8	1.000
1047	o173 conc	82388.0	170879.	-88491.4	1.000
1048	o174 conc	144291.	164491.	-20200.1	1.000
1049	o175 conc	161528.	158144.	3384.35	1.000
1050	o176 conc	128019.	151858.	-23839.4	1.000
1051	o177 conc	108437.	145654.	-37217.3	1.000
1052	o178 conc	110262.	139548.	-29286.0	1.000
1053	o179 conc	121310.	133554.	-12244.2	1.000
1054	o180 conc	133760.	127685.	6074.57	1.000
1055	o181 conc	139392.	121953.	17439.2	1.000
1056	o182 conc	129247.	116366.	12881.4	1.000
1057	o183 conc	116109.	110932.	5177.12	1.000
1058	o184	126217.	105658.	20558.7	1.000

	conc					
1059	o185	139498.	100550.	38947.5	1.000	conc
1060	o186	126431.	95613.4	30817.6	1.000	conc
1061	o187	110561.	90851.3	19709.7	1.000	conc
1062	o188	110899.	86268.3	24630.7	1.000	conc
1063	o189	109252.	81868.0	27384.0	1.000	conc
1064	o190	99126.8	77654.8	21472.0	1.000	conc
1065	o191	92048.0	73633.4	18414.6	1.000	conc
1066	o192	89688.8	69809.3	19879.5	1.000	conc
1067	o193	88557.5	66189.3	22368.2	1.000	conc
1068	o194	94958.0	62781.8	32176.2	1.000	conc
1069	o195	107079.	59597.0	47482.0	1.000	conc
1070	o196	91369.6	56647.1	34722.5	1.000	conc
1071	o197	46576.9	53946.5	-7369.56	1.000	conc
1072	o198	25748.9	51511.9	-25763.0	1.000	conc
1073	o199	33799.4	49362.4	-15563.0	1.000	conc
1074	o200	35631.0	47519.0	-11888.0	1.000	conc
1075	o201	28383.7	46004.2	-17620.5	1.000	conc
1076	o202	23252.8	44841.7	-21588.9	1.000	conc
1077	o203	26145.9	44055.3	-17909.4	1.000	conc
1078	o204	36201.1	43668.2	-7467.12	1.000	conc
1079	o205	41993.2	43701.6	-1708.43	1.000	conc
1080	o206	42503.4	44173.6	-1670.19	1.000	conc
1081	o207	49159.4	45097.5	4061.86	1.000	conc
1082	o208	59113.1	46480.9	12632.2	1.000	conc
1083	o209	53550.1	48323.7	5226.42	1.000	conc
1084	o210	42313.1	50617.2	-8304.11	1.000	conc
1085	o211	49127.3	53343.1	-4215.77	1.000	conc
1086	o212	59932.4	56472.3	3460.11	1.000	conc
1087	o213	57765.5	59964.9	-2199.44	1.000	conc
1088	o214	59051.1	63770.1	-4718.98	1.000	conc
1089	o215	71289.7	67826.2	3463.47	1.000	conc
1090	o216	78099.1	72062.3	6036.84	1.000	conc
1091	o217	74866.7	76398.8	-1532.08	1.000	conc
1092	o218	69517.8	80749.9	-11232.1	1.000	conc
1093	o219	70436.8	85025.6	-14588.8	1.000	conc
1094	o220	82025.2	89133.7	-7108.51	1.000	conc

1095	o221 conc	92322.6	92983.2	-660.646	1.000
1096	o222 conc	93098.2	96486.6	-3388.37	1.000
1097	o223 conc	93041.5	99562.3	-6520.77	1.000
1098	o224 conc	100680.	102138.	-1457.67	1.000
1099	o225 conc	120120.	104151.	15969.1	1.000
1100	o226 conc	135730.	105553.	30177.0	1.000
1101	o227 conc	127404.	106309.	21095.2	1.000
1102	o228 conc	121217.	106398.	14818.8	1.000
1103	o229 conc	136763.	105816.	30946.6	1.000
1104	o230 conc	129342.	104573.	24768.6	1.000
1105	o231 conc	88468.1	102694.	-14225.4	1.000
1106	o232 conc	70850.2	100214.	-29364.0	1.000
1107	o233 conc	80996.3	97184.2	-16187.9	1.000
1108	o234 conc	79578.4	93661.8	-14083.4	1.000
1109	o235 conc	71476.0	89712.8	-18236.8	1.000
1110	o236 conc	74543.8	85408.2	-10864.4	1.000
1111	o237 conc	73858.3	80821.7	-6963.44	1.000
1112	o238 conc	60598.7	76028.3	-15429.6	1.000
1113	o239 conc	54071.0	71101.3	-17030.3	1.000
1114	o240 conc	58191.7	66111.2	-7919.53	1.000
1115	o241 conc	57334.7	61124.0	-3789.34	1.000
1116	o242 conc	52277.3	56199.9	-3922.55	1.000
1117	o243 conc	52213.6	51392.1	821.543	1.000
1118	o244 conc	53457.5	46746.7	6710.81	1.000
1119	o245 conc	51425.7	42302.1	9123.59	1.000
1120	o246 conc	50764.7	38089.0	12675.7	1.000
1121	o247 conc	52332.4	34130.3	18202.1	1.000
1122	o248 conc	49494.8	30442.1	19052.7	1.000
1123	o249 conc	41204.9	27033.6	14171.3	1.000
1124	o250 conc	31483.7	23908.4	7575.33	1.000
1125	o251 conc	24486.3	21064.6	3421.69	1.000
1126	o252 conc	21590.2	18496.2	3093.97	1.000
1127	o253 conc	18193.9	16193.6	2000.32	1.000
1128	o254 conc	13790.9	14144.2	-353.305	1.000
1129	o255 conc	13243.4	12333.6	909.761	1.000
1130	o256 conc	14937.6	10746.0	4191.56	1.000
1131	o257	13632.5	9364.79	4267.71	1.000

	conc				
1132	o258 conc	9520.91	8173.04	1347.87	1.000
1133	o259 conc	5061.07	7154.14	-2093.07	1.000
1134	o260 conc	3493.82	6291.94	-2798.12	1.000
1135	o261 conc	5447.37	5571.14	-123.769	1.000
1136	o262 conc	7290.70	4977.40	2313.30	1.000
1137	o263 conc	7251.73	4497.52	2754.21	1.000
1138	o264 conc	5666.69	4119.46	1547.23	1.000
1139	o265 conc	4002.47	3832.40	170.074	1.000
1140	o266 conc	3705.62	3626.64	78.9758	1.000
1141	o267 conc	4079.18	3493.62	585.560	1.000
1142	o268 conc	4342.79	3425.74	917.050	1.000
1143	o269 conc	4657.45	3416.31	1241.14	1.000
1144	o270 conc	4709.12	3459.41	1249.71	1.000
1145	o271 conc	3662.07	3549.75	112.317	1.000
1146	o272 conc	2533.16	3682.59	-1149.43	1.000
1147	o273 conc	2971.71	3853.54	-881.827	1.000
1148	o274 conc	3480.80	4058.53	-577.727	1.000
1149	o275 conc	2837.98	4293.65	-1455.67	1.000
1150	o276 conc	3585.37	4555.12	-969.747	1.000
1151	o277 conc	5772.33	4839.13	933.195	1.000
1152	o278 conc	5009.78	5141.90	-132.117	1.000
1153	o279 conc	2281.37	5459.53	-3178.16	1.000
1154	o280 conc	2563.73	5788.06	-3224.33	1.000
1155	o281 conc	4521.34	6123.45	-1602.11	1.000
1156	o282 conc	4986.85	6461.54	-1474.69	1.000
1157	o283 conc	5110.52	6798.16	-1687.64	1.000
1158	o284 conc	6018.91	7129.10	-1110.19	1.000
1159	o285 conc	6769.64	7450.19	-680.553	1.000
1160	o286 conc	7514.82	7757.34	-242.524	1.000
1161	o287 conc	9606.69	8046.62	1560.07	1.000
1162	o288 conc	11461.7	8314.29	3147.41	1.000
1163	o289 conc	10743.1	8556.91	2186.19	1.000
1164	o290 conc	10720.9	8771.33	1949.57	1.000
1165	o291 conc	13733.1	8954.84	4778.26	1.000
1166	o292 conc	14813.8	9105.11	5708.69	1.000
1167	o293 conc	11710.4	9220.33	2490.07	1.000

1168	o294 conc	7841.36	9299.16	-1457.80	1.000
1169	o295 conc	6105.19	9340.79	-3235.60	1.000
1170	o296 conc	6112.11	9344.95	-3232.84	1.000
1171	o297 conc	6769.24	9311.87	-2542.63	1.000
1172	o298 conc	7018.63	9242.30	-2223.67	1.000
1173	o299 conc	6603.01	9137.47	-2534.46	1.000
1174	o300 conc	6064.89	8999.04	-2934.15	1.000
1175	o301 conc	5981.44	8829.10	-2847.66	1.000
1176	o302 conc	6769.91	8630.06	-1860.15	1.000
1177	o303 conc	8101.42	8404.63	-303.209	1.000
1178	o304 conc	9429.78	8155.74	1274.04	1.000
1179	o305 conc	10216.6	7886.52	2330.08	1.000
1180	o306 conc	10232.1	7600.16	2631.94	1.000
1181	o307 conc	9650.45	7299.94	2350.51	1.000
1182	o308 conc	8673.18	6989.11	1684.07	1.000
1183	o309 conc	7502.49	6670.86	831.634	1.000
1184	o310 conc	6345.57	6348.24	-2.67393	1.000
1185	o311 conc	5411.55	6024.19	-612.644	1.000
1186	o312 conc	4907.69	5701.43	-793.739	1.000
1187	o313 conc	4888.55	5382.45	-493.902	1.000
1188	o314 conc	5145.72	5069.52	76.2003	1.000
1189	o315 conc	5444.77	4764.63	680.138	1.000
1190	o316 conc	5566.72	4469.52	1097.20	1.000
1191	o317 conc	5430.42	4185.64	1244.78	1.000
1192	o318 conc	5027.17	3914.19	1112.98	1.000
1193	o319 conc	4349.18	3656.10	693.077	1.000
1194	o320 conc	3446.13	3412.06	34.0730	1.000
1195	o321 conc	2499.34	3182.51	-683.166	1.000
1196	o322 conc	1708.47	2967.69	-1259.22	1.000
1197	o323 conc	1259.21	2767.65	-1508.44	1.000
1198	o324 conc	1157.75	2582.25	-1424.50	1.000
1199	o325 conc	1285.64	2411.23	-1125.59	1.000
1200	o326 conc	1521.98	2254.17	-732.193	1.000
1201	o327 conc	1760.85	2110.57	-349.716	1.000
1202	o328 conc	1942.61	1979.81	-37.2040	1.000
1203	o329 conc	2016.51	1861.25	155.256	1.000
1204	o330	1934.51	1754.18	180.332	1.000

	conc				
1205	o331 conc	1702.19	1657.85	44.3446	1.000
1206	o332 conc	1373.90	1571.50	-197.602	1.000
1207	o333 conc	1005.74	1494.39	-488.651	1.000
1208	o334 conc	649.533	1425.76	-776.230	1.000
1209	o335 conc	339.055	1364.89	-1025.83	1.000
1210	o336 conc	103.355	1311.06	-1207.70	1.000
1211	o337 conc	0.00000	1263.60	-1263.60	1.000
1212	o338 conc	0.00000	1221.89	-1221.89	1.000
1213	o339 conc	0.00000	1185.31	-1185.31	1.000
1214	o340 conc	0.00000	1153.31	-1153.31	1.000
1215	o341 conc	0.00000	1125.37	-1125.37	1.000
1216	o342 conc	0.00000	1101.03	-1101.03	1.000
1217	o343 conc	0.00000	1079.85	-1079.85	1.000
1218	o344 conc	24.6039	1061.44	-1036.83	1.000
1219	o345 conc	189.070	1045.44	-856.374	1.000
1220	o346 conc	371.669	1031.55	-659.883	1.000
1221	o347 conc	489.454	1019.48	-530.024	1.000
1222	o348 conc	467.298	1008.97	-541.676	1.000
1223	o349 conc	323.288	999.819	-676.531	1.000
1224	o350 conc	136.783	991.819	-855.036	1.000
1225	o351 conc	0.00000	984.804	-984.804	1.000
1226	o352 conc	0.00000	978.628	-978.628	1.000
1227	o353 conc	0.00000	973.162	-973.162	1.000
1228	o354 conc	0.00000	968.296	-968.296	1.000
1229	o355 conc	15.9872	963.936	-947.949	1.000
1230	o356 conc	27.6263	960.000	-932.374	1.000
1231	o357 conc	20.8621	956.419	-935.557	1.000
1232	o358 conc	0.00000	953.135	-953.135	1.000
1233	o359 conc	0.00000	950.098	-950.098	1.000
1234	o360 conc	0.00000	947.266	-947.266	1.000
1235	o361 conc	0.00000	944.604	-944.604	1.000
1236	o362 conc	38.6360	942.083	-903.447	1.000
1237	o363 conc	161.098	939.677	-778.579	1.000
1238	o364 conc	316.812	937.367	-620.555	1.000
1239	o365 conc	483.800	935.136	-451.336	1.000
1240	o366 conc	642.739	932.969	-290.230	1.000

1241	o367	conc	788.596	930.856	-142.260	1.000
1242	o368	conc	921.125	928.787	-7.66157	1.000
1243	o369	conc	1039.77	926.753	113.017	1.000
1244	o370	conc	1127.28	924.750	202.530	1.000
1245	o371	conc	1141.63	922.771	218.859	1.000
1246	o372	conc	1038.82	920.813	118.007	1.000
1247	o373	conc	787.834	918.872	-131.038	1.000
1248	o374	conc	454.309	916.946	-462.637	1.000
1249	o375	conc	147.371	915.032	-767.661	1.000
1250	o376	conc	0.00000	913.129	-913.129	1.000
1251	o377	conc	0.00000	911.235	-911.235	1.000
1252	o378	conc	178.614	909.350	-730.736	1.000
1253	o379	conc	464.396	907.471	-443.075	1.000
1254	o380	conc	806.740	905.599	-98.8592	1.000
1255	o381	conc	1155.84	903.733	252.107	1.000
1256	o382	conc	1461.89	901.873	560.017	1.000
1257	o383	conc	1676.50	900.018	776.482	1.000
1258	o384	conc	1760.11	898.167	861.943	1.000
1259	o385	conc	1676.55	896.322	780.228	1.000
1260	o386	conc	1390.50	894.480	496.020	1.000
1261	o387	conc	931.442	892.644	38.7983	1.000
1262	o388	conc	437.972	890.811	-452.839	1.000
1263	o389	conc	59.1618	888.983	-829.821	1.000
1264	o390	conc	0.00000	887.159	-887.159	1.000
1265	o391	conc	26.7570	885.339	-858.582	1.000
1266	o392	conc	267.289	883.523	-616.234	1.000
1267	o393	conc	557.398	881.710	-324.312	1.000
1268	o394	conc	817.031	879.902	-62.8713	1.000
1269	o395	conc	991.351	878.098	113.253	1.000
1270	o396	conc	1028.94	876.298	152.642	1.000
1271	o397	conc	885.073	874.501	10.5719	1.000
1272	o398	conc	598.276	872.708	-274.432	1.000
1273	o399	conc	263.623	870.920	-607.297	1.000
1274	o400	conc	0.00000	869.135	-869.135	1.000
1275	o401	conc	0.00000	867.353	-867.353	1.000
1276	o402	conc	0.00000	865.576	-865.576	1.000
1277	o403		0.00000	863.803	-863.803	1.000

conc

o404	152.637	862.033	-709.396	1.000	conc
o405	486.728	860.267	-373.539	1.000	conc
o406	803.196	858.504	-55.3085	1.000	conc
o407	999.271	856.746	142.525	1.000	conc
o408	1001.11	854.991	146.119	1.000	conc
o409	853.094	853.240	-0.146196	1.000	conc
o410	629.831	851.493	-221.662	1.000	conc
o411	404.922	849.749	-444.827	1.000	conc
o412	221.358	848.009	-626.651	1.000	conc
o413	86.4305	846.273	-759.843	1.000	conc
o414	5.41412	844.541	-839.127	1.000	conc
o415	0.00000	842.812	-842.812	1.000	conc
o416	0.00000	841.087	-841.087	1.000	conc
o417	1.83637	839.365	-837.529	1.000	conc
o418	0.00000	837.648	-837.648	1.000	conc
o419	20.0022	835.934	-815.931	1.000	conc
o420	167.357	834.223	-666.866	1.000	conc
o421	356.022	832.516	-476.494	1.000	conc
o422	487.104	830.813	-343.709	1.000	conc
o423	472.761	829.113	-356.352	1.000	conc
o424	331.322	827.417	-496.095	1.000	conc
o425	140.303	825.725	-685.422	1.000	conc
o426	0.00000	824.036	-824.036	1.000	conc
o427	0.00000	822.351	-822.351	1.000	conc
o428	0.00000	820.669	-820.669	1.000	conc
o429	0.00000	818.991	-818.991	1.000	conc
o430	77.2770	817.317	-740.040	1.000	conc
o431	224.980	815.646	-590.666	1.000	conc
o432	377.267	813.978	-436.711	1.000	conc
o433	507.381	812.315	-304.934	1.000	conc
o434	591.128	810.654	-219.526	1.000	conc
o435	612.737	808.998	-196.261	1.000	conc
o436	558.166	807.344	-249.178	1.000	conc
o437	415.570	805.695	-390.125	1.000	conc
o438	220.447	804.048	-583.601	1.000	conc
o439	53.8837	802.406	-748.522	1.000	conc

1314	o440 conc	0.00000	800.766	-800.766	1.000
1315	o441 conc	108.455	799.131	-690.676	1.000
1316	o442 conc	303.139	797.498	-494.359	1.000
1317	o443 conc	466.067	795.869	-329.802	1.000
1318	o444 conc	483.141	794.244	-311.103	1.000
1319	o445 conc	338.699	792.622	-453.923	1.000
1320	o446 conc	141.219	791.004	-649.785	1.000
1321	o447 conc	7.10519	789.389	-782.283	1.000
1322	o448 conc	31.8670	787.777	-755.910	1.000
1323	o449 conc	183.043	786.169	-603.126	1.000
1324	o450 conc	379.758	784.564	-404.806	1.000
1325	o451 conc	541.471	782.963	-241.492	1.000
1326	o452 conc	618.586	781.365	-162.779	1.000
1327	o453 conc	612.838	779.770	-166.932	1.000
1328	o454 conc	530.791	778.179	-247.388	1.000
1329	o455 conc	382.484	776.591	-394.107	1.000
1330	o456 conc	207.541	775.007	-567.466	1.000
1331	o457 conc	60.5508	773.426	-712.875	1.000
1332	o458 conc	0.00000	771.848	-771.848	1.000
1333	o459 conc	46.1642	770.274	-724.109	1.000
1334	o460 conc	192.871	768.703	-575.832	1.000
1335	o461 conc	411.737	767.135	-355.398	1.000
1336	o462 conc	675.194	765.571	-90.3766	1.000
1337	o463 conc	918.134	764.010	154.124	1.000
1338	o464 conc	1050.34	762.452	287.888	1.000
1339	o465 conc	981.197	760.897	220.300	1.000
1340	o466 conc	681.595	759.346	-77.7513	1.000
1341	o467 conc	304.360	757.798	-453.438	1.000
1342	o468 conc	35.7788	756.254	-720.475	1.000
1343	o469 conc	51.8214	754.713	-702.891	1.000
1344	o470 conc	338.819	753.175	-414.356	1.000
1345	o471 conc	716.697	751.640	-34.9430	1.000
1346	o472 conc	999.732	750.109	249.623	1.000
1347	o473 conc	1047.59	748.580	299.010	1.000
1348	o474 conc	902.303	747.055	155.248	1.000
1349	o475 conc	651.723	745.534	-93.8107	1.000
1350	o476	382.911	744.015	-361.104	1.000

```
conc
o477                  159.502       742.500      -582.998       1.000
conc
o478                  18.2148       740.988      -722.773       1.000
conc
o479                  0.00000       739.479      -739.479       1.000
conc
o480                  107.393       737.973      -630.580       1.000
conc
o481                  291.838       736.471      -444.633       1.000
conc
o482                  452.788       734.972      -282.184       1.000
conc
o483                  496.542       733.476      -236.934       1.000
conc
o484                  393.985       731.983      -337.998       1.000
conc
o485                  213.383       730.493      -517.110       1.000
conc
o486                  31.0702       729.007      -697.936       1.000
conc
o487                  0.00000       727.523      -727.523       1.000
conc
o488                  0.00000       726.043      -726.043       1.000
conc
o489                  0.00000       724.566      -724.566       1.000
conc
o490                  30.4946       723.092      -692.597       1.000
conc
o491                  180.699       721.621      -540.922       1.000
conc
o492                  340.231       720.153      -379.922       1.000
conc
o493                  471.098       718.689      -247.591       1.000
conc
o494                  538.884       717.227      -178.343       1.000
conc
o495                  547.549       715.769      -168.220       1.000
conc
o496                  524.384       714.313      -189.929       1.000
conc
o497                  496.995       712.861      -215.866       1.000
conc
o498                  484.919       711.412      -226.493       1.000
conc
o499                  486.180       709.966      -223.786       1.000
conc
o500                  495.254       708.523      -213.269       1.000
conc

See file mfit.res for more details of residuals in graph-ready format.

See file mfit.seo for composite observation sensitivities.


Objective function ----->

  Sum of squared weighted residuals (ie phi)              =  8.4623E+10


Correlation Coefficient ----->

  Correlation coefficient                                 =  0.97756


Analysis of residuals ----->

  All residuals:-
     Number of residuals with non-zero weight                     =   500
     Mean value of non-zero weighted residuals                    =   539.2
     Maximum weighted residual [observation "o163"]               =  6.2461E+04
     Minimum weighted residual [observation "o173"]               = -8.8491E+04
     Standard variance of weighted residuals                      =  1.7341E+08
```

```
    Standard error of weighted residuals                          =  1.3168E+04

    Note: the above variance was obtained by dividing the objective
    function by the number of system degrees of freedom (ie. number of
    observations with non-zero weight plus number of prior information
    articles with non-zero weight minus the number of adjustable parameters.)
    If the degrees of freedom is negative the divisor becomes
    the number of observations with non-zero weight plus the number of
    prior information items with non-zero weight.


Covariance and other statistical matrices cannot be determined:-
Memory conservation is operative so that covariance matrix is not calculated.
```
