# Supplementary figures and images for: Comparing the Fate and Transport of MS2 Bacteriophage and Sodium Fluorescein in a Karstic Chalk Aquifer
Source: Pathogens. 2024 Feb 13;13(2):168. doi: 10.3390/pathogens13020168 (PMC10891704; doi:10.3390/pathogens13020168)

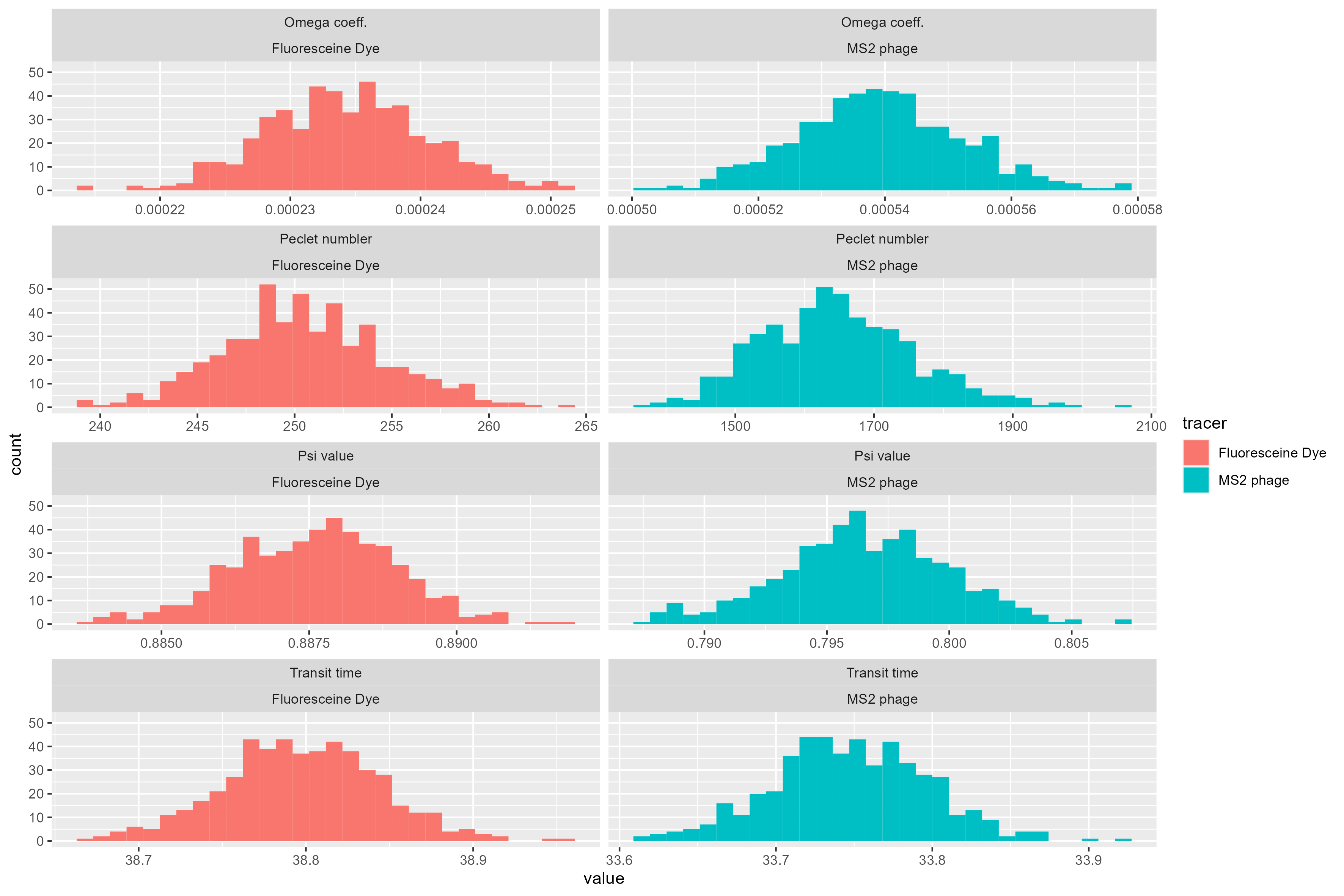

Supplement: Supplementary file 1 [file pathogens-13-00168-s001.zip › ABH1_PCUA.png]

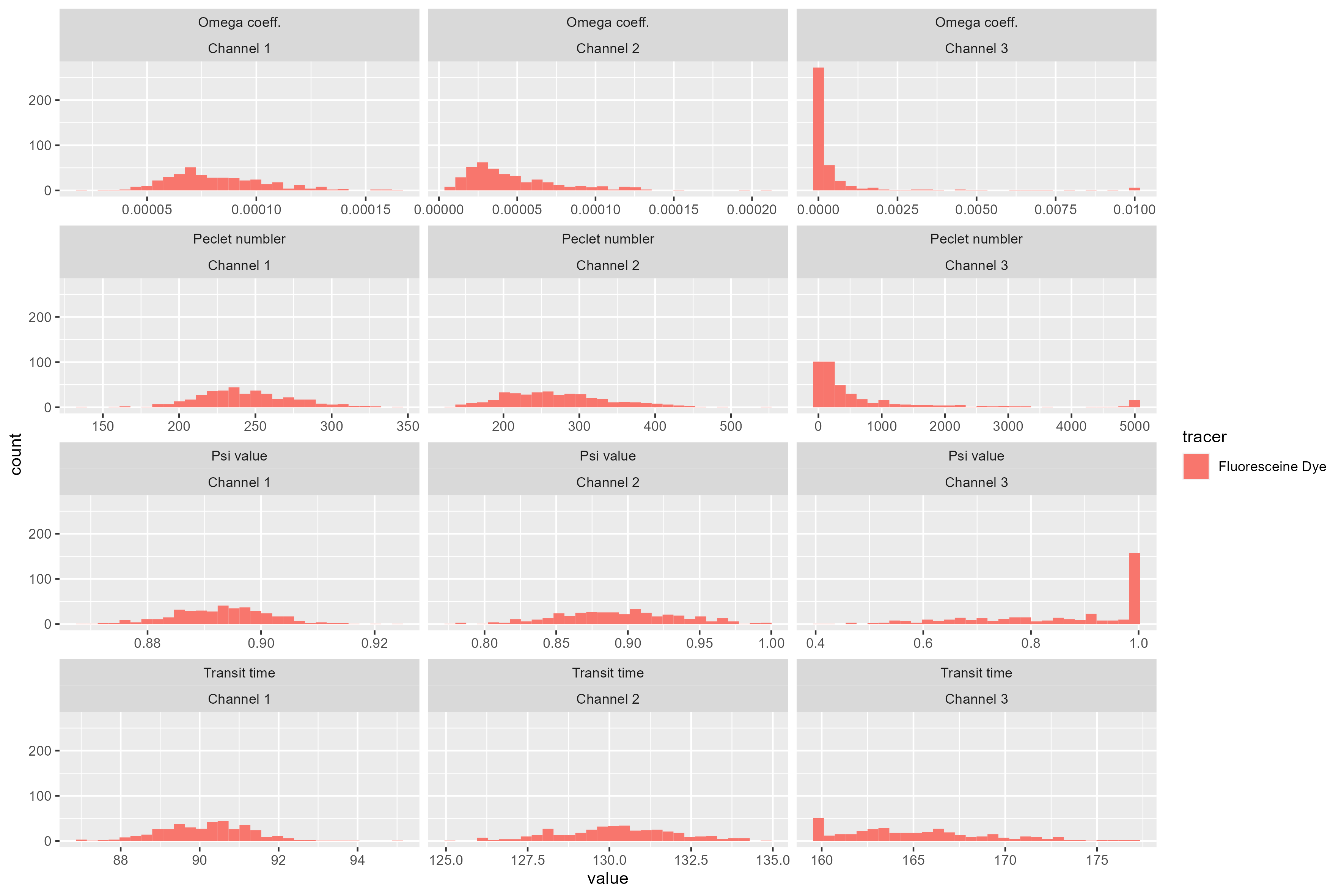

Supplement: Supplementary file 1 [file pathogens-13-00168-s001.zip › ABH2_PCUA_DYE.png]

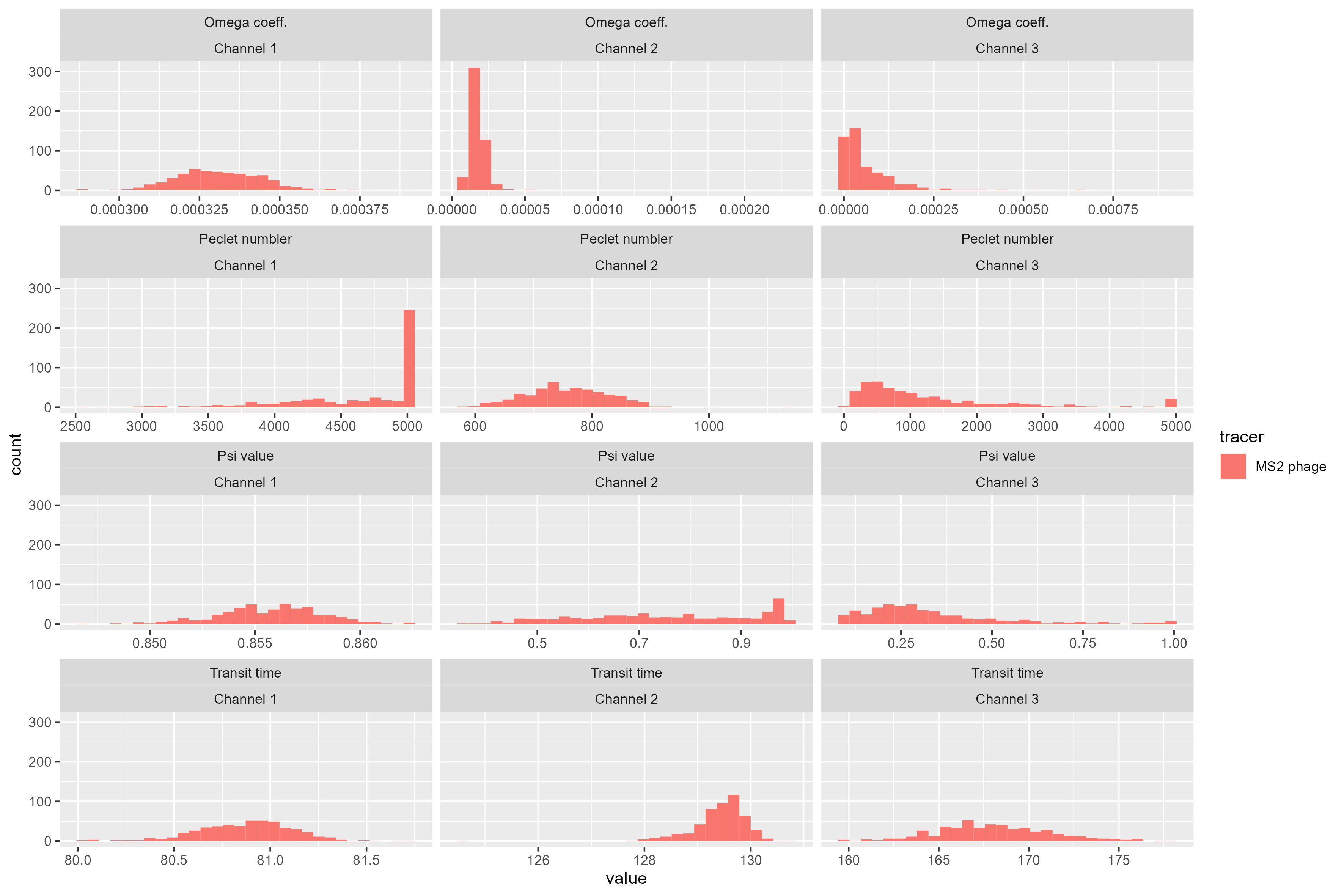

Supplement: Supplementary file 1 [file pathogens-13-00168-s001.zip › ABH2_PCUA_PHAGE.png]
